# Bayesian analysis and prediction of hybrid performance

**DOI:** 10.1186/s13007-019-0388-x

**Published:** 2019-02-07

**Authors:** Filipe Couto Alves, Ítalo Stefanine Correa Granato, Giovanni Galli, Danilo Hottis Lyra, Roberto Fritsche-Neto, Gustavo de los Campos

**Affiliations:** 1 Departments of Epidemiology and Biostatistics, Statistics and Probability and Institute of Quantitative Health Science and Engineering, Michigan State University, 775 Woodlot Dr. Office 1311, East Lansing, USA; 2 Department of Epidemiology and Biostatistics, Michigan State University, 775 Woodlot Dr. Office 1315, East Lansing, USA; 3 Department of Genetics, “Luiz de Queiroz” College of Agriculture, University of São Paulo, Avenida Pádua Dias, No 11, Piracicaba, São Paulo Brazil; 4 Department of Computational and Analytical Sciences, Rothamsted Research, Harpenden, UK

**Keywords:** Bayesian models, Genomic prediction, Hybrid prediction, Convergent populations, Tropical maize, BGLR, Semi-parametric models, RKHS, Dominance, Epistasis, Non-additive effects, Specific combining ability, Nitrogen, Stress

## Abstract

**Background:**

The selection of hybrids is an essential step in maize breeding. However, evaluating a large number of hybrids in field trials can be extremely costly. However, genomic models can be used to predict the expected performance of un-tested genotypes. Bayesian models offer a very flexible framework for hybrid prediction. The Bayesian methodology can be used with parametric and semi-parametric assumptions for additive and non-additive effects. Furthermore, samples from the posterior distribution of Bayesian models can be used to estimate the variance due to general and specific combining abilities even in cases where additive and non-additive effects are not mutually orthogonal. Also, the use of Bayesian models for analysis and prediction of hybrid performance has remained fairly limited.

**Results:**

We provided an overview of Bayesian parametric and semi-parametric genomic models for prediction of agronomic traits in maize hybrids and discussed how these models can be used to decompose the genotypic variance into components due to general and specific combining ability. We applied the methodology to data from 906 single cross tropical maize hybrids derived from a convergent population. Our results show that: (1) non-additive effects make a sizable contribution to the genetic variance of grain yield; however, the relative importance of non-additive effects was much smaller for ear and plant height; (2) genomic prediction can achieve relatively high accuracy in predicting phenotypes of un-tested hybrids and in pre-screening.

**Conclusions:**

Genomic prediction can be a useful tool in pre-screening of hybrids and could contribute to the improvement of the efficiency and efficacy of maize hybrids breeding programs. The Bayesian framework offers a great deal of flexibility in modeling hybrid performance. The methodology can be used to estimate important genetic parameters and render predictions of the expected hybrid performance as well measures of uncertainty about such predictions.

**Electronic supplementary material:**

The online version of this article (10.1186/s13007-019-0388-x) contains supplementary material, which is available to authorized users.

## Background

Most commercial maize breeding programs perform selection on inbred lines and then select optimal crosses among elite materials (often from divergent heterotic groups) to produce commercial hybrids. Single crosses are highly homogeneous, can express heterosis, have greater yield stability in marginal environments, and are a convenient way to stack traits controlled by large-effect dominant genes [1]. Furthermore, hybrids are appealing for seed companies because they can generate sustained demand for seeds. These biological and commercial advantages prompted the adoption of hybrids in many crops, being maize the most prominent one.

Selecting optimal matings is a critical aspect of any maize hybrid breeding program. Ideally, one would choose crosses based on the observed agronomic performance in field trials. However, evaluating all possible crosses can be extremely expensive, especially in the early stages of a breeding program when the number of candidate lines is often large. In this situation, only a small fraction of all the possible crosses can be evaluated in field experiments [2]. Genomic models can be used to predict the performance of un-tested hybrids; therefore, genomic prediction (GP, e.g. [3]), a methodology initially developed for selection and breeding, also arises as a promising approach in hybrid prediction and mate selection.

Most of the theoretical and applied GP studies have focused on prediction of traits and diseases in outbreed materials from either animal [4–7] and plant [8–12] breeding populations. Genomic models are also often used for prediction of agronomic traits in inbred lines [13–17]. More recently, some authors have considered using genomic models for prediction of hybrid performance [18–25]; these studies have shown that genomic models can yield reasonably accurate predictions of the agronomic performance of hybrids.

Bayesian models offer great flexibility for the study and prediction of hybrid performance. The framework allows modeling hybrid performance using parametric and semi-parametric methods. Samples from the posterior distributions from these models can be used to infer important parameters, such as the variance due to general and specific combining abilities, that are difficult to estimate when the mating design does not allow for an orthogonal decomposition of the genetic variance into those components. Furthermore, in addition to the prediction of expected hybrid performance, samples from the posterior distribution can be used to quantify the uncertainty of the predicted performance while accounting for uncertainty about other model parameters. These features make the Bayesian approach particularly well-suited for analysis and prediction of hybrid performance. In this manuscript, we present *an overview of Bayesian genomic models for prediction of agronomic traits* in maize hybrids and use the described models to evaluate the contribution of additive and non-additive effects for prediction of agronomic traits in tropical maize.

Most of the literature on the genomic analysis of hybrid performance in maize has focused on the study of materials produced by crossing lines from divergent heterotic groups. Crosses from such groups are expected to express less specific-combining ability than the one expected among crosses of lines showing a small degree of divergence among heterotic groups [26, 27]. Here, we focus *on the evaluation of additive and non*-*additive effects models when applied to predict crosses of inbred lines from a convergent population*.

In the hybrid prediction literature, the genetic variance is often decomposed into the general and specific combining ability variance (GCA and SCA, respectively, [28]) components. The GCA variance represents the amount of variance that can be explained by the differences between the average performance of the parental lines in crosses and the overall population mean, while the SCA variance quantifies the amount of variance on the genotypic values that cannot be explained by parental means. This component is often attributable to deviations from additivity due to dominance and epistasis [29]. Unfortunately, in genomic analyses, additive and non-additive contrasts are often not mutually orthogonal. For this reason, the variance parameters entering in genomic models (e.g., the additive and dominance variance) cannot be directly used to decompose the total genetic variance into GCA and SCA components. Here, following ideas presented by Lehermeier et al. [30] *we discuss how GCA and SCA variance components can be estimated in Bayesian models including additive and several types of non*-*additive effects*, regardless of the orthogonality of contrasts used to accommodate those effects.

Several studies in the prediction of hybrid performance are based on parametric models for additive and dominance effects modeling [18, 23, 25, 31], and a few studies have considered the inclusion of epistatic interactions (e.g., [10, 12, 21, 32]). However, the additive-by-additive epistatic relationship matrices often used (which relies on Hadamard products of additive relationship matrices) do not allow for a clear distinction of the contribution dominance and epistasis [33]. W*e used ideas earlier presented by Martini* et al. [33] *to build kernels that enable an explicit distinction between dominance and additive*-*by*-*additive epistatic interaction*. Furthermore, we consider semi-parametric alternatives (Reproducing Kernel Hilbert Spaces, RKHS, [34, 35]) that can capture both additive and non-additive effects.

What remains of the manuscript is organized as follows: the next section describes a general Bayesian framework for the hybrid prediction that encompasses parametric and semi-parametric methods in a unified setting. In this section, we also discuss methods to estimate variance due to general and specific combining ability. Subsequently, we applied the methods described to a data set of hybrids from a convergent population and reported both variance components and predictive performance.

### Genomic models for analysis of hybrid data

**Fig. 1:**
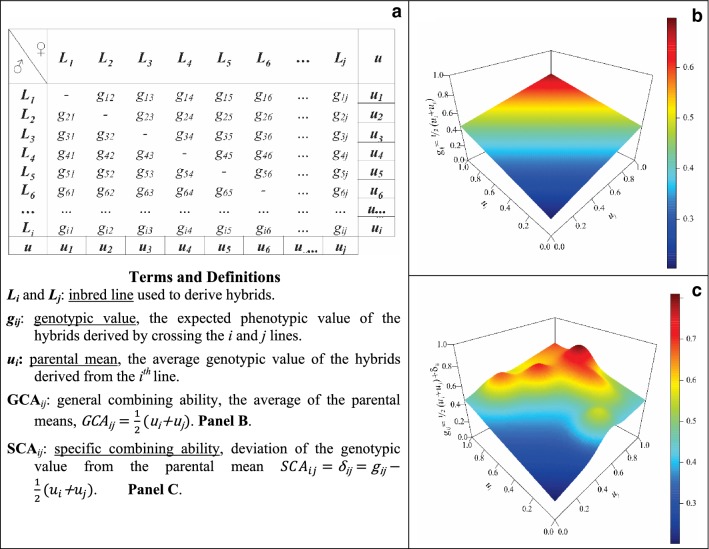
Prediction of hybrid performance using genomic regression models. **a** The grid shows all possible crosses between *n* lines ($i = j$) in a diallel mating design. **b** Hyper-plane generated by the general combining abilities of females and males. **c** Hypothetical hybrid performance surface influenced by both additive and non-additive effects (module)

The problem of predicting the hybrid performance for all the possible crosses that can be generated from *n* lines can be viewed as one of smoothing phenotypic data (e.g., yield observed on hybrids) over a grid of crosses (Fig. 1). In Fig. 1 the left panel represents all possible crosses from a group of inbred lines, and the right plot describes surfaces with different degree of genetic complexity. The phenotype of the *k*th replicate of the progeny of lines *i* and *j* (${{y}}_{{ijk}}$) can be decomposed into a genetic component (${{g}}_{{ij}}$) plus a residual effect ($\upvarepsilon_{{ijk}}$) that is ${{y}}_{{ijk}} = {{g}}_{{ij}} +\upvarepsilon_{{ijk}}$, in which $\varepsilon_{ijk} {}_{\sim }^{iid} N\left( {0,\sigma_{\varepsilon }^{2} } \right)$. Here, ${{g}}_{{ij}}$ represents the expected phenotypic performance (average over replicates) of the progeny of lines *i* and *j* that is ${{g}}_{{ij}} = {{E(y}}_{{ijk}} )$. Ideally, we would like to predict ${{g}}_{{ij}}$ for all possible crosses (i.e., all possible (*i, j*) pairs for *i* ≠ *j*). This task can be achieved by smoothing phenotypic data with respect to genotypes. The surface’s smoothness (right panel of Fig. 1) depends on the relationship among the inbred lines and on the types of modeled genetics effects in ${{g}}_{{ij}}$. The additive model gives the smoothest pattern (a hyperplane), whereas accounting for dominance and epistasis make this surfaces more irregular (compare the top and lower right plots of Fig. 1).

### General and specific combining abilities

The expected hybrid performance ($${{g}}_{{ij}}$$) is often represented as the sum of general and specific combining abilities (GCA and SCA, respectively, [28]).^1^ The GCA-portion of a hybrid’s genotypic value ($${{GCA}}_{{ij}}$$) is the average of the parental means, $${{GCA}}_{{ij}} { = }\frac{ 1}{ 2}\left( {u_{i} { + }u_{j} } \right)$$; here,$$u_{{i}} = E_{j|i} \left( {{{g}}_{{ij}} } \right)$$ represent the average genotypic value of the progeny of *i*th parental line in respect to the second parents (likewise, $$u_{{j}} = E_{i|j} \left( {{{g}}_{{ij}} } \right)$$). Traditionally, the general combining ability of a line ($$u$$) is obtained by the (deviation of the) marginal mean of the parental line (relative to the population mean) in all crosses/hybrids in which it appears (Fig. 1a, $$u$$). The SCA portion of the hybrid’s genotypic value accounts for deviations of the hybrid mean ($${{g}}_{{ij}} )$$ relative to the average of the parental means, that is $$\updelta_{{ij}} = {{g}}_{{ij}} { - }\frac{ 1}{ 2}\left( {u_{i} { + }u_{j} } \right)$$. Thus, from the perspective of Analysis of Variance, the GCA represent the main effects of the parental lines and SCA represent interactions between those lines. The total genetic variance can ten be decomposed into two orthogonal components, that is $$\sigma_{G}^{2} = \sigma_{GCA}^{2} + \sigma_{SCA}^{2}$$ where $$\sigma_{G}^{2} = Var\left( {{\text{g}}_{\text{ij}} } \right)$$, $$\sigma_{GGA}^{2} = Var\left( {GCA_{ij} } \right)$$ and $$\sigma_{SCA}^{2} = Var\left( {SCA_{ij} } \right)$$.

The GCA of a line depends on the allele substitution effects, thus the general combining ability involves not only additive but also non-additive effects [29, 36, 37]. In genomic models, the average allele substitution effects can be inferred by fitting a pure additive model: $u_{i} = \mathop \sum \nolimits_{k = 1}^{p} x_{ik} \alpha_{k}$ where $x_{ik} \in \left\{ {0,2} \right\}$ is the genotype of the *i*th line at the *k*th SNP and $\alpha_{k}$ is the additive effect of the *k*th SNP. For crosses derived from very divergent populations (different heterotic groups), accounting for group-specific effects is often necesary [18, 22, 24, 38, 39]. However, this is not needed in cases where crosses involve lines from convergent populations. In this case, the general combining ability can be expressed as:

1 $$Additive \, model \, \left( A \right){:}\;{\text{g}}_{\text{ij}} = \frac{1}{2}\left( {u_{i} + u_{j} } \right) = \mathop \sum \limits_{k = 1}^{p} x_{ijk} \alpha_{k}$$

where $x_{ijk} = \frac{1}{2}\left( {x_{ik} + x_{jk} } \right)$ is the hybrid genotype at the *k*th loci which is simply the average of the parental genotypes. The additive model [expression (1)] defines a hyperplane with respect to the general combining abilities (Fig. 1b). Deviations from the hyperplane (SCA effects) can be introduced by adding dominance and epistatic interactions in the prediction model.

Dominance effects (i.e., within locus interaction of alleles, $\beta_{k}$) can be accommodated by adding dummy-variables for heterozygous loci that is

2 $$Additive + Dominance \, \left( {A + D} \right){:}\;{\text{g}}_{\text{ij}} = \mathop \sum \limits_{k = 1}^{p} x_{ijk} \alpha_{k} + \mathop \sum \limits_{k = 1}^{p} \Delta_{ijk} \beta_{k} ;$$

where $\Delta_{ijk} = 1(x_{ijk} = 1)$ is an indicator variable for the *k*th loci that takes value 1 for heterozygous and 0 for homozygous loci.

Epistatic interactions can take various forms (additive-by-additive, additive-by-dominance, dominance by dominance, additive-by-additive-by-additive, etc. [29, 40]); for simplicity, in parametric models, we focus on first order interaction of alleles between loci involving additive and dominance effects, that is additive-by-additive and additive-by-dominance interactions. With *p* markers, we can form $\frac{{p\left( {p - 1} \right)}}{2}$ additive-by-additive (*A by A)* interactions; a prediction model including additive, dominance, and additive-by-additive interactions ($\gamma_{kl}$) effects takes the form

3a $$Additive + Dominance \, + A - by - A\left( {A + D + AA} \right){:}\;{\text{g}}_{\text{ij}} = \mathop \sum \limits_{k = 1}^{p} x_{ijk} \alpha_{k} + \mathop \sum \limits_{k = 1}^{p} \Delta_{ijk} \beta_{k} + \mathop \sum \limits_{k = 1}^{p} \mathop \sum \limits_{l > k}^{p} x_{ijk} x_{ijl} \gamma_{kl}$$

Likewise, one can have a total of $\frac{{p\left( {p - 1} \right)}}{2}$ additive-by-dominance interactions (*A by D,* $\omega_{kl}$) which can be combined with additive and dominance effects to give rise to the following model:

4a $$Additive + Dominance + A \, by \, D\left( {A + D + AD} \right){:}\;{\text{g}}_{\text{ij}} = \mathop \sum \limits_{k = 1}^{p} x_{ijk} \alpha_{k} + \mathop \sum \limits_{k = 1}^{p} \Delta_{ijk} \beta_{k} + \mathop \sum \limits_{k = 1}^{p} \mathop \sum \limits_{l > k}^{p} x_{ijk} \Delta_{ijl} \omega_{kl}$$

In models involving additive and non-additive effects, the coefficients associated to the additive portion of the model (the $\alpha_{k}$’s) no longer represent allele substitution effects. For this reason, the variance parameters associated to this coefficients cannot be directly used to estimate the variance due to GCA. We address the estimation of GCA and SCA variance in models involving nonadditive effects later in this section.

### Parametric kernels for additive and non-additive effects

The number of effects entering on [1–4] can be extremely large. Therefore, in genomic models, effects are usually treated as random draws from some distribution, being the most common the Normal distribution.

The terms on the right side of equations [1–4] are linear combinations of effects. Therefore, if effects follow normal distributions, $\alpha_{k} {}_{\sim }^{iid} N\left( {0,\sigma_{a}^{2} } \right)$, $\beta_{k} {}_{\sim }^{iid} N\left( {0,\sigma_{d}^{2} } \right)$, $\gamma_{kl} {}_{\sim }^{iid} N\left( {0,\sigma_{aa}^{2} } \right)$ and $\omega_{kl} {}_{\sim }^{iid} N\left( {0,\sigma_{ad}^{2} } \right)$, then, vectors containing additive $\varvec{a} = \left\{ {a_{ij} = \mathop \sum \nolimits_{k = 1}^{p} x_{ijk} \alpha_{k} } \right\}$, dominance $\varvec{d} = \left\{ {d_{ij} = \mathop \sum \nolimits_{k = 1}^{p} \Delta_{ijk} \beta_{k} } \right\}$, additive-by-additive $\varvec{aa} = \left\{ {aa_{ij} = \mathop \sum \nolimits_{k = 1}^{p} \mathop \sum \nolimits_{l > k}^{p} x_{ijk} x_{ijl} \gamma_{kl} } \right\}$, and additive-by-dominance epistatic interactions $\varvec{ad} = \left\{ {ad_{ij} = \mathop \sum \nolimits_{k = 1}^{p} \mathop \sum \nolimits_{l > k}^{p} x_{ijk} \Delta_{ijl} \omega_{kl} } \right\}$ will follow multivariate normal distributions: $\varvec{a\sim}MVN\left( {0,\varvec{K}_{a} \sigma_{a}^{2} } \right)$, $\varvec{d\sim}MVN\left( {0,\varvec{K}_{d} \sigma_{d}^{2} } \right)$, $\varvec{aa\sim}MVN\left( {0,\varvec{K}_{aa} \sigma_{aa}^{2} } \right)$ and $\varvec{ad\sim}MVN\left( {0,\varvec{K}_{ad} \sigma_{ad}^{2} } \right)$ where $\varvec{K}_{a}$, $\varvec{K}_{d}$, $\varvec{K}_{aa}$ and $\varvec{K}_{ad}$ are co-variance matrices for additive, dominance, additive-by-additive, and additive-by-dominance effects, respectively.

The covariance matrices for additive and dominance effects ($\varvec{K}_{a} \varvec{ }$ and $\varvec{K}_{d}$**)** are well established (e.g., [41–43]), and can be computed using cross-products of genotypes codes: $\varvec{K}_{a} = \frac{{\varvec{XX^{\prime}}}}{{tr\left( {\varvec{XX^{\prime}}} \right)/n}}$ where $\varvec{X} = \left\{ {x_{ijk} - 2\theta_{jk} } \right\}$ is a matrix of centered hybrid genotypes (here $\theta_{jk}$ is the frequency of the allele counted at the *k*th loci) and $\varvec{K}_{d} = \frac{{\varvec{DD^{\prime}}}}{{tr\left( {\varvec{DD^{\prime}}} \right)/n}}$ where $\varvec{D} = \left\{ {1(x_{ijk} = 1) - 2\theta_{jk} \left( {1 - \theta_{jk} } \right)} \right\}$ is a matrix whose columns contain dummy variables for heterozygous genotypes centered around their respective means.

Computing the covariance structure for additive-by-additive and additive-by-dominance effects is more challenging because the number of contrasts involved can be substantial. However, these covariance matrices can be computed using Hadamard products [44]. For instance, the covariance matrix for additive-by-dominance effects can be computed using the Hadamard product (denoted by “$\odot$”) between $\varvec{K}_{a}$ and $\varvec{K}_{d}$ (see [33, 44]); hence, $\varvec{K}_{ad} = \frac{{\varvec{K}_{{\mathbf{a}}} \odot \varvec{K}_{{\mathbf{d}}} }}{{tr\left( {\varvec{K}_{{\mathbf{a}}} \odot \varvec{K}_{{\mathbf{d}}} } \right)/n}}$. Martini et al. [33] showed that the Hadamard product $\varvec{K}_{a} \odot \varvec{K}_{a}$ includes cross-products of contrasts for additive-by-additive effects and also cross-products of contrasts for dominance (see also Additional file 1: Supplementary Methods 1). Therefore, the correct covariance structure for additive-by-additive effects can be obtained by subtracting the contribution of dominance, that is: $\varvec{K}_{aa} = \frac{{\left( {\varvec{XX^{\prime}}} \right) \odot \left( {\varvec{XX^{\prime}}} \right)^{\varvec{'}} - \left( {\varvec{X} \odot \varvec{X}} \right)\left( {\varvec{X} \odot \varvec{X}} \right)^{\varvec{'}} }}{{tr\left( {\left( {\varvec{XX^{\prime}}} \right) \odot \left( {\varvec{XX^{\prime}}} \right)^{\varvec{'}} - \left( {\varvec{X} \odot \varvec{X}} \right)\left( {\varvec{X} \odot \varvec{X}} \right)^{\varvec{'}} } \right)/n}}$.

The covariance structures discussed in the previous section can be used in Bayesian multivariate normal distributions to model the hybrid’s genetic/genotypic values. For instance, for expression (3), we have ${\text{g}} = \varvec{a} + \varvec{d} + \varvec{aa}$ and thus

3b $${\text{g }} \sim MVN\left( {0,\varvec{K}_{a} \sigma_{a}^{2} + \varvec{K}_{d} \sigma_{d}^{2} + \varvec{K}_{aa} \sigma_{aa}^{2} } \right)$$

Likewise, for expression (4), we have ${\text{g}} = \varvec{a} + \varvec{d} + \varvec{ad}$, thus

4b $${\text{g}} \sim MVN\left( {0,\varvec{K}_{a} \sigma_{a}^{2} + \varvec{K}_{d} \sigma_{d}^{2} + \varvec{K}_{ad} \sigma_{ad}^{2} } \right)$$

### Semi-parametric procedures

The models of expressions (3b) and (4b) can be viewed as Bayesian multi-kernel models where different kernels are used to accommodate different types of effects. Each of these kernels also defines a different degree of smoothness of genetic values with respect to genotypes, with $\varvec{K}_{a}$ usually giving higher smoothness (i.e., more covariance) than dominance or epistatic kernels. In such multi-kernel models, the variance parameters act as weights which end up defining the smoothness of **g** [35]. For semi-parametric smoothing, we can replace the parametric kernels with, for example, Gaussian kernels indexed with different bandwidth parameters ($h$). For instance, one can assume

5 $${\text{g}} \sim MVN\left( {0,\varvec{K}_{{h_{1} }} \sigma_{1}^{2} + \varvec{K}_{{h_{2} }} \sigma_{2}^{2} + \varvec{K}_{{h_{3} }} \sigma_{3}^{2} } \right)$$

This approach referred as “kernel averaging” in de los Campos et al. [35], can be used to infer smooth functions without making parametric assumptions. Recently, Lyra et al. [23] and Sousa et al. [45] used a single-kernel regression to predict hybrid performance. Here we consider multi-kernel methods based on three Gaussian kernels derived from an additive relationship matrix. The proposed approach derives a matrix of genetic distances from $\varvec{K}_{a}$. These distances are then used as inputs in three Gaussian kernels with values of the bandwidth parameters chosen so that one of the kernels gives higher covariance than additive effects, another one gives lower covariances than additive effects, and the last one gives covariances smaller than the two former kernels. The proposed approach has a built-in standardization such that the values of the bandwidth parameters do not depend on the number of markers used. Further details are given in Additional file 1: Supplementary Methods 2.

### Estimating total genomic variance and its components in multi-kernel models

Traditionally, in the literature of hybrid prediction, the total genetic variance and its components (GCA and SCA) have been derived directly from variance parameters [18, 22, 38]. For instance, for multi-kernel models, such those in equations [3a] and [3b], the total genetic variance is inferred using the sum of each of the variance parameters (e.g., $\sigma_{G}^{2} = \sigma_{a}^{2} + \sigma_{d}^{2} + \sigma_{aa}^{2} )$. Likewise, commonly, the GCA is equated to the variance associated with the additive term (e.g., $\sigma_{GCA}^{2} = \sigma_{a}^{2}$, [18, 22, 38]). This approach has at least two potential problems. First, as noted before, in models involving non-additive effects, the additive component ($\sigma_{a}^{2} )$ does not represent the variance due to the average effect of allele substitution. Thus, to fully account for allele substitution effects, $\sigma_{GCA}^{2}$ needs to be estimated from a purely additive model. Second, in multi-kernel models, the total genetic variance cannot be estimated using the sum of the variance parameters because that approach ignores covariances between terms. For instance, in the model of expressions (3a) and (3b), the total variance is $Var\left( G \right) = Var\left( a \right) + Var\left( d \right) + Var\left( {aa} \right) + 2\left[ {Cov\left( {a, d} \right) + Cov\left( {a, aa} \right) + Cov\left( {d,aa} \right)} \right]$ (see [30] for a more in-depth discussion of the topic).

As noted by Lehermeier et al. [30], in a Bayesian setting, samples from the posterior distribution of the total genetic variance that account for covariances between terms can be obtained from samples (from the posterior distribution) of effects. For instance, suppose that one has vectors containing realizations from the posterior distribution of vectors of additive ($\varvec{a}_{s}$), dominance ($\varvec{d}_{s}$) and additive-by-additive effects ($\varvec{aa}_{s}$), all obtained at the *s*th iteration of a sampler from an A + D+AA model [3b]. The total genetic value in this model is $\varvec{g}_{s} = \varvec{a}_{s} + \varvec{d}_{s} + \varvec{aa}_{s}$. At this iteration, the total genomic variance is $\sigma_{{G_{s} }}^{2} = Var\left( {\varvec{g}_{s} } \right) = Var\left( {\varvec{a}_{s} + \varvec{d}_{s} + \varvec{aa}_{s} } \right)$, where $Var()$ is the sample variance operator, $\sigma_{{G_{s} }}^{2} = \left( {n - 1} \right)^{ - 1} \mathop \sum \nolimits_{i = 1}^{n} \left( {g_{is} - \bar{g}_{s} } \right)^{2}$ where $\bar{g}_{s} = n^{ - 1} \mathop \sum \nolimits_{i = 1}^{n} g_{is}$ is the average genomic value in the sample at the *s*th iteration. Likewise, the covariance between additive and non-additive terms can be computed using the sample covariance operator, $Cov\left( {\varvec{a}_{s} ,\varvec{d}_{s} } \right) = \left( {n - 1} \right)^{ - 1} \mathop \sum \nolimits_{i = 1}^{n} \left( {a_{is} - \bar{a}_{s} } \right)\left( {d_{is} - \bar{d}_{s} } \right)$. The supplemental scripts (Additional File 2) provided with the manuscript illustrate how variance and covariance components were estimated using the BGLR package [46].

Therefore, to fully account for allele substitution effects covariances between additive and non-additive effects we estimate the total genetic variance and its GCA and SCA components as follows: (1) we estimated the **general combining ability variance** ($\sigma_{GCA}^{2}$) by evaluating, at each iteration of the sampler, the sample variance of the additive effects collected using a pure additive model (Eq. [1]). (2) We estimate the total genetic variance by evaluating at each iteration of the sampler the total variance explained by a model including additive and non-additive effects ($\sigma_{{G_{s} }}^{2}$). (3) The $\sigma_{SCA}^{2}$ is estimated as the difference between the total genomic variance (estimated using a model that includes additive and non-additive effects) and the additive variance derived from a purely additive model ($\sigma_{GCA}^{2}$). Finally, the proportion of the total genomic variance attributable to SCA can be estimated using $D^{2} = \frac{{\sigma_{SCA}^{2} }}{{\sigma_{G}^{2} }}$.

### Application to a dataset of tropical maize hybrids derived from a convergent population

We used the models described above to study the performance of hybrids obtained by crossing lines from a convergent population.

*Data* were available for a total of 906 maize single-crosses derived from forty-nine inbred lines crossed in an unbalanced diallel mating design. The average (min/max) number of times that each inbred line appeared as parental in our dataset was 37 (18/48, see Additional file 3: Fig. S1). The hybrids were evaluated during the second growing season (January to May), of 2016 and 2017, in two locations, Piracicaba (PI; rainfed; 22°42′23″S, 47°38′14″W, 535 m) and Anhembi (AN; irrigated; 22°50′51″S, 48°01′06″W, 466 m), São Paulo State, Brazil. At each site, the material was evaluated under two nitrogen (N) regimes, ideal N (IN; 100 ${\text{kg}}\,{\text{N}}\,{\text{ha}}^{ - 1}$, 70 ${\text{kg}}\,{\text{N}}\,{\text{ha}}^{ - 1}$ at sowing and 30 ${\text{kg}}\,{\text{N}}\,{\text{ha}}^{ - 1}$ on the V8 plant stage) and low N (LN; 30 ${\text{kg}}\,{\text{N}}\,{\text{ha}}^{ - 1}$ being the totality applied at sowing). These two treatments, in combination with the two locations, were used to define four distinct environments (PI.IN, PI.LN, AN.IN, AN.LN).

*Field trials* were organized in an unreplicated augmented block design consisting of 47 (year 1) or 50 (year 2) blocks with 16 hybrids and two commercial checks evaluated in each block. **Three traits** were evaluated in each environment: grain yield (GY, ${\text{ton}}\, {\text{ha}}^{ - 1}$), plant height (PH, $m$), and ear height (EH,$m$). Plots were manually harvested and GY was corrected to 13% moisture. EH and PH were measured from the soil surface until the insertion of the first ear and the flag leaf collar on five representative plants within each plot, respectively.

*Phenotypes were pre*-*adjusted* using a mixed model with an intercept, the fixed effect of the check, and the random effect of the block. We used this model to derive an adjusted phenotype for each trait, which consisted of the measured phenotype minus the estimated intercept minus the block effect. Finally, we averaged the adjusted phenotype of each hybrid from years 1 and 2 to carry out the genomic analyses.

*Genotypes* for each one of the forty-nine parental inbred lines were obtained using the Affymetrix^®^ Axiom^®^ Maize Genotyping Array of 616 K SNPs [47]. Markers with call rate lower than 0.90, heterozygous loci in at least one parental line, and all non-mapped SNPs were removed. Hybrids genotypes were derived from the parental genotypes. Allele frequencies and pairwise linkage disequilibrium (*r*^*2*^) statistics were computed using hybrid genotypes. SNPs with minor allele frequency smaller than 0.05 were excluded. Afterward, the hybrids genotypes matrix was pruned to guarantee a maximum *r*^*2*^ between SNPs smaller than 0.9. All quality control procedures were made using the R package *synbreed* [48], and LD pruning was carried out using the *SNPRelate R* package [49]. After all quality control and LD pruning process, 34,571 high-quality SNPs were available to further analysis. The pairwise linkage disequilibrium between loci (*r*^*2*^) in the parental inbred lines for each one of the ten chromosomes are shown in Additional file 4: Fig. S2.

*For genomic analyses,* we used the multi-kernel regressions described above to a defined sequence of models of increasing complexity: from strictly additive models to semi-parametric regressions. Models were fitted using the BGLR R-package [46]. Variance components estimates were obtained (using the methods described in the previous section) by fitting each model to data from each environment separately (full-data analysis). For each model, inferences were based on 30,000 samples collected after discarding 5000 samples for burn-in and thinning of 5. The convergence of the Markov chains and Monte Carlo Error was assessed using the coda R-package [50]. We inspected trace plots for each of the variance parameters to be sure if the burn-in period was sufficient. Moreover, for each of the variance parameters, we checked that the effective number of independent samples was greater than 100 and the estimated Monte Carlo Error smaller than 1% of the estimated posterior mean.

Subsequently, we estimated prediction accuracy by fitting models using training–testing partitions (TRN-TST). In each TRN-TST partition, 75% of the data (approximately 680 hybrids) was randomly selected to constitute the training set (TRN), whereas the remaining 25% of the hybrids formed the testing set (TST) and were employed to evaluate the model’s predictive ability. Since all data from each hybrid was assigned to the same fold, our evaluation of prediction accuracy is similar to the method labeled as CV1 in Burgueño et al. [51] and also comparable to the T2 method presented by Technow et al. [22, 38]. The same training–testing partitions were used to fit each of the models; this allowed us to compute the proportion of times that one model achieved higher prediction accuracy than the other ones.

Predictive performance was measured using the Pearson’s product moment correlation between adjusted phenotypes and genomic estimated genetic values ($r_{{y\hat{y}}}$) in each of the TST sets. For each model/trait/environment, we carried out a total of 100 TRN-TST partitions, totaling 100 correlations estimates. The models’ predictive abilities were compared by the Tuckey’s Honest Significance Difference test at 5% significance.

## Results

**Fig. 2:**
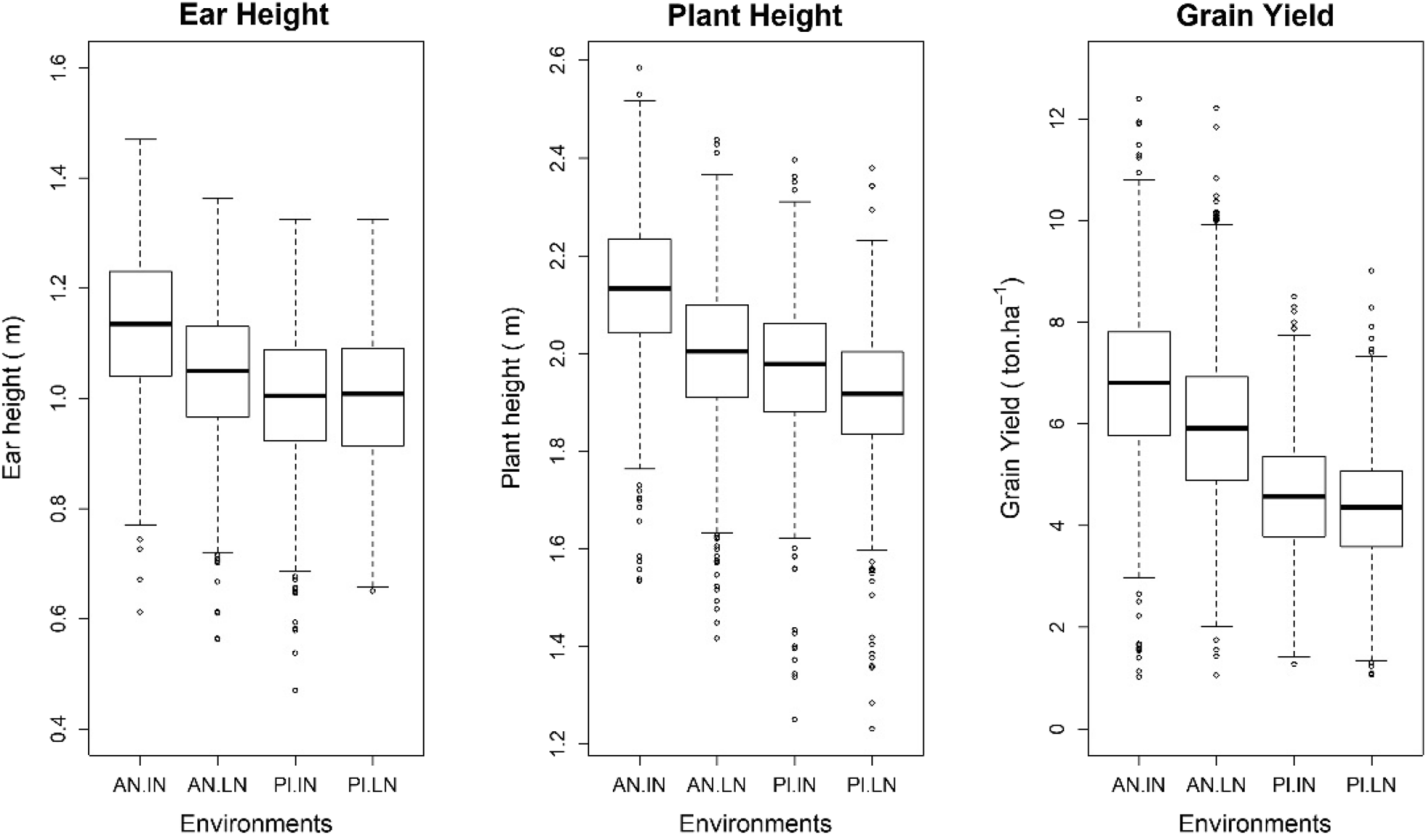
Boxplot of phenotypes by trait and environments. AN.IN: Anhembi ideal nitrogen regime; AN.LN: Anhembi low nitrogen regime; PI.IN: Piracicaba ideal nitrogen regime; PI.LN: Piracicaba low nitrogen regime

The average ear height, plant height, and grain yield were higher in the irrigated environment (Anhembi) than in the rainfed one (Piracicaba). These traits also had higher average values under ideal nitrogen (especially in well-watered conditions) than with low-nitrogen availability (Fig. 2). For all the traits and environments, the observed distributions of phenotypes were seemingly symmetric, and there were no significant differences in variances (except for grain yield, for this trait the variance of phenotypes was higher in well-watered conditions).

**Fig. 3:**
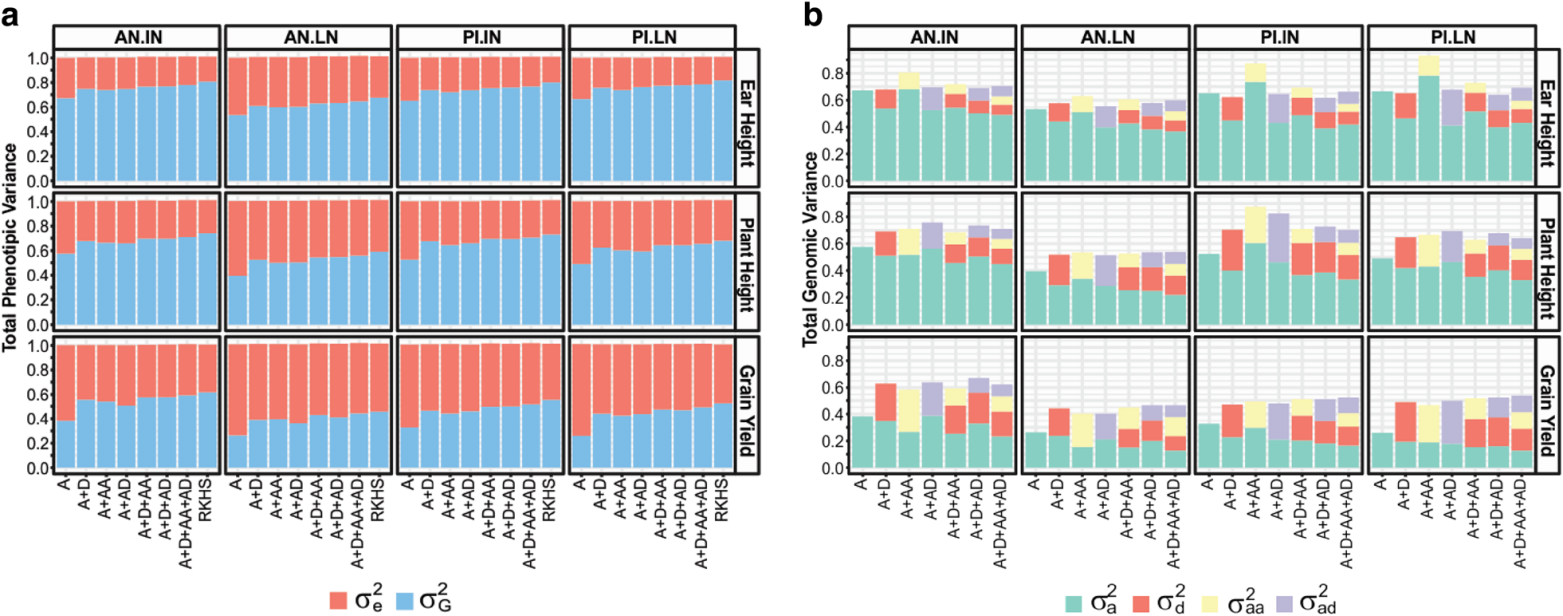
Variance components (**a**) and variance parameters (**b**). **a** Estimated genetic variance explained by the model ($\sigma_{G}^{2} )$, and estimated error variance ($\sigma_{e}^{2} )$. **b** Individual variance parameters. AN.IN: Anhembi ideal nitrogen regime; AN.LN: Anhembi low nitrogen regime; PI.IN: Piracicaba ideal nitrogen regime; PI.LN: Piracicaba low nitrogen regime. A: Additive, D: Dominance, AA: Additive × additive, and AD: Additive × dominance effects. RKHS: Reproducing Kernel Hilbert Spaces model). $\sigma_{a}^{2}$, $\sigma_{d}^{2}$, $\sigma_{aa}^{2}$,and $\sigma_{ad}^{2}$, additive, dominance, additive by additive, additive by dominance genetic parameters, respectively

*Genomic variance and broad*-*sense genomic heritability (H*^*2*^*)* The proportion of variance of phenotypes explained by the model ($H^{2} = \frac{{\sigma _{G}^{2} }}{{\sigma _{G}^{2} + \sigma _{\varepsilon }^{2} ) }}$) was highest for EH (ranging from 0.7 to ~ 0.8, depending on the environment and model, Fig. 3a), intermediate for PH and lowest for GY (for this trait values ranged from ~ 0.3 to ~ 0.6). The comparison across environments shows that the proportion of variance that can be explained by genetic factors was highest in the best environmental conditions (AN.IN) and lowest in AN.LN and with either low or ideal N in Piracicaba, where trials were not irrigated. As one would expect, the proportion of variance explained by the model increased when terms accounting for non-additive effects were included in the model (Fig. 3).

In general, there was a sizable increase in the proportion of variance explained when dominance was included in the model and relatively small increases in $\sigma _{G}^{2}$ when other effects were added to the A + D model. The difference in $\sigma _{G}^{2}$ between the A and A + D models was smaller for EH and sizable for GY (Fig. 3a). The RKHS model showed the highest estimates of the $H^{2}$. However, in general, this model did not explain much more variance than the A + D model.

As one would expect the inclusion of non-additive effects reduced the estimate of $\sigma_{a}^{2}$; this happens because in models involving non-additive effects $\sigma_{a}^{2}$ no longer represents the total variance explained by allele substitution effects (Fig. 3b, Additional file 5: Tables S1–S5).

**Fig. 4:**
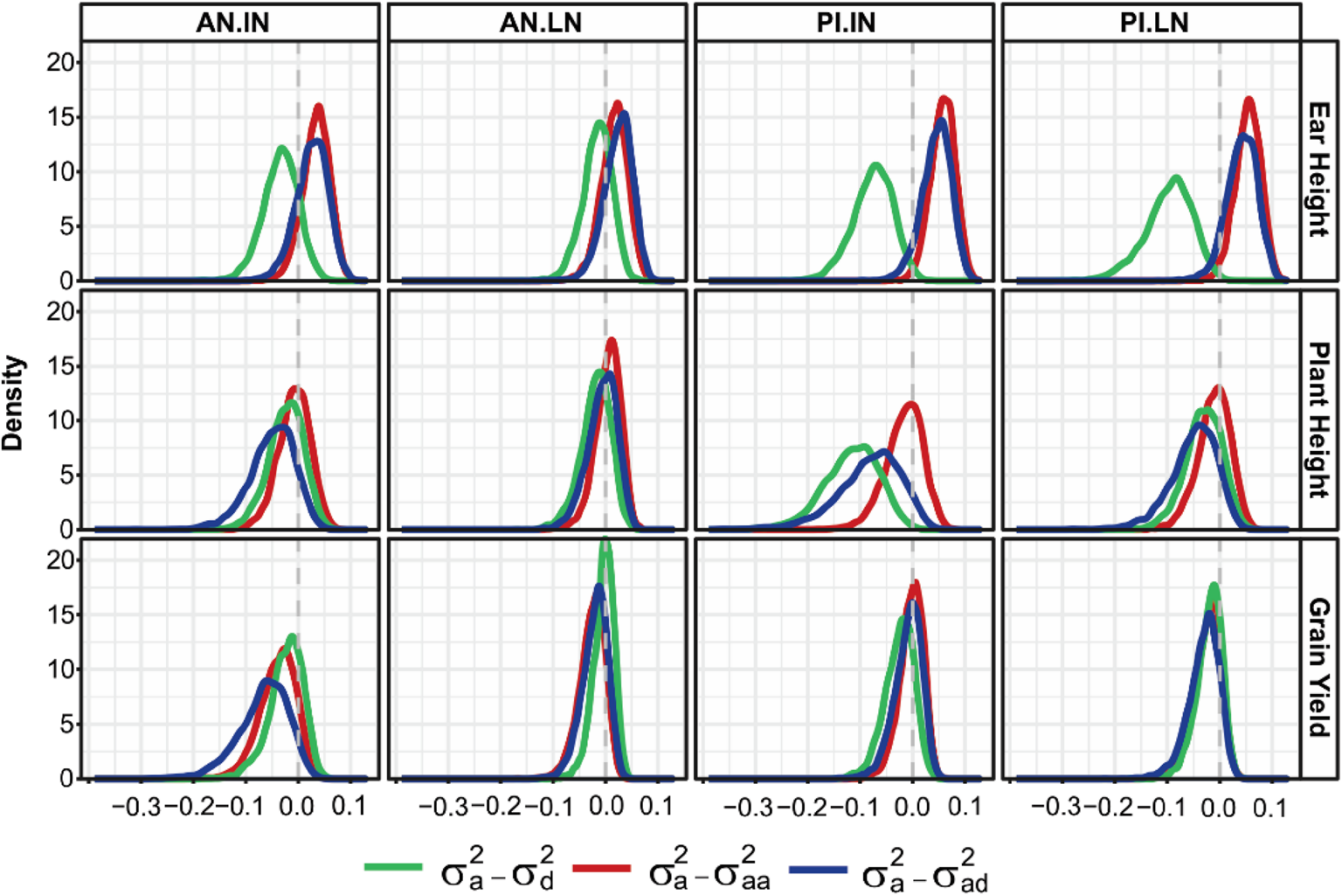
Posterior density of the covariance between the additive and non-additive genetic components of models including two genetic terms by environment and traits. AN and PI: Anhembi and Piracicaba. IN and LN: ideal and low nitrogen availability. Covariance between effects in the A + D model is represented in green ($\sigma_{a}^{2}$–$\sigma_{d}^{2}$); Covariance between effects in the A + AA model is represented in red ($\sigma_{a}^{2}$–$\sigma_{aa}^{2}$); Covariance between effects in the A + AD model is represented in blue ($\sigma_{a}^{2}$–$\sigma_{ad}^{2}$). AN: Anhembi, PI: Piracicaba, IN: Ideal nitrogen, and LN: Low nitrogen. A: Additive effect, and D: Dominance effects. A: Additive effect, D: Dominance, AA: Additive-additive, and AD: Additive-dominance effects

Using samples from the posterior distribution, we evaluated covariances between additive and non-additive effects. We found different covariance patterns for the different traits (Fig. 4 and Additional file 6: Fig. S3). Among them, EH was the trait that appeared to be mostly additive (Fig. 3a), and the average covariance between additive and additive-by-additive variances and those among additive and additive-by-dominance were slightly positive. On the other hand, for PH and GY most of the covariances were close to zero, with a few exceptions (e.g., PH in PI.IN).

*The estimated variance components* ($\sigma _{G}^{2}$, $\sigma _{GCA}^{2}$ and $\sigma _{SCA}^{2}$) were used to compute the *proportion of variance explained by non*-*additive effects* ($D^{2} = \frac{{\sigma _{SCA}^{2} }}{{\sigma_{G}^{2} }}$, Table 1). *D*^*2*^ was highest for grain yield (*D*^*2*^ values ranging from ~ 0.236 to ~ 0.47) and lowest for EH (*D*^*2*^ estimates ranged from ~ 0.08 to ~ 0.17). PH represented an intermediate case with *D*^*2*^ ranging from ~ 0.12 to ~ 0.29 (Table 1, see Additional file 7: Table S6 for estimates of $SCA_{ratio} = \frac{{\sigma_{SCA}^{2} }}{{\sigma_{GCA}^{2} }}$). For all traits, non-additive effects contributed more to the variance under low N conditions relative to high N availability. The optimal environment (AN.IN, ideal nitrogen regime and irrigated conditions) showed the smallest contribution of non-additive effects for the three traits.

**Table 1:** Posterior mean (posterior SD) of the proportion of genetic variance explained by non-additive effects (D^2^), by trait, model, and environment

Trait	Env	Model						
		A + D	A + AA	A + AD	A + D+AA	A + D+AD	A + D+AA + AD	Mean
EH	AN.IN	0.099^(0.057)^	0.088^(0.058)^	0.097^(0.057)^	0.120^(0.053)^	0.123^(0.053)^	0.135^(0.052)^	0.110
	AN.LN	0.117^(0.077)^	0.104^(0.078)^	0.111^(0.078)^	0.150^(0.071)^	0.153^(0.070)^	0.172^(0.068)^	0.135
	PI.IN	0.115^(0.059)^	0.094^(0.060)^	0.114^(0.060)^	0.134^(0.056)^	0.140^(0.056)^	0.150^(0.054)^	0.124
	PI.LN	0.119^(0.056)^	0.097^(0.058)^	0.125^(0.055)^	0.138^(0.054)^	0.143^(0.054)^	0.154^(0.052)^	0.129
PH	AN.IN	0.150^(0.065)^	0.130^(0.069)^	0.123^(0.070)^	0.172^(0.063)^	0.171^(0.063)^	0.186^(0.061)^	0.155
	AN.LN	0.238^(0.086)^	0.204^(0.092)^	0.210^(0.091)^	0.269^(0.082)^	0.273^(0.079)^	0.292^(0.076)^	0.248
	PI.IN	0.220^(0.065)^	0.184^(0.070)^	0.202^(0.071)^	0.243^(0.062)^	0.243^(0.063)^	0.254^(0.061)^	0.224
	PI.LN	0.213^(0.071)^	0.183^(0.077)^	0.172^(0.079)^	0.236^(0.068)^	0.236^(0.068)^	0.249^(0.067)^	0.215
GY	AN.IN	0.302^(0.078)^	0.284^(0.085)^	0.236^(0.094)^	0.331^(0.073)^	0.333^(0.074)^	0.352^(0.071)^	0.306
	AN.LN	0.307^(0.110)^	0.326^(0.105)^	0.260^(0.119)^	0.379^(0.095)^	0.352^(0.096)^	0.399^(0.088)^	0.337
	PI.IN	0.286^(0.099)^	0.253^(0.103)^	0.282^(0.102)^	0.335^(0.088)^	0.339^(0.088)^	0.361^(0.082)^	0.309
	PI.LN	0.405^(0.093)^	0.380^(0.098)^	0.395^(0.102)^	0.446^(0.082)^	0.440^(0.086)^	0.466^(0.079)^	0.422

*A* additive, *D* Dominance, *AA* additive × additive, *AD* additive × dominance effects. *AN.IN* Anhembi ideal nitrogen regime; *AN.LN* Anhembi low nitrogen regime; *PI.IN* Piracicaba ideal nitrogen regime; *PI.LN* Piracicaba low nitrogen regime. *EH*, *GY*, and *PH*: ear height, grain yield, and plant height, respectively

### Prediction accuracy

The cross-validation analyses yielded moderately high prediction correlations ($r_{{y\hat{y}}}$), ranging from ~ 0.46 to ~ 0.81 (Table 2). Prediction accuracy was highest for EH, smaller but still high for PH, and moderate for GY. The lowest mean correlations were obtained in AN.LN. For EH the predictive performance was very similar in the other three environments. On the other hand, for GY and PH, the prediction accuracies were smaller in stressed conditions (low nitrogen availability) than in the “ideal” ones (Table 2).

**Table 2:** Prediction accuracy of models by environments and traits

Models	Environment: AN.IN			Environment: AN.LN		
	EH	PH	GY	EH	PH	GY
A	0.805^a^	0.74^ab^	0.581^d^	0.710^a^	0.595^a^	0.462^a^
A + D	*0.809* ^a^	*0.750* ^a^	*0.618* ^a^	*0.711* ^a^	*0.611* ^a^	*0.481* ^a^
A + AA	0.805^a^	0.740^ab^	0.597^bcd^	0.708^a^	0.601^a^	0.478^a^
A + AD	0.804^a^	0.740^ab^	0.579^d^	0.708^a^	0.594^a^	0.463^a^
A + D+AA	0.806^a^	0.745^ab^	0.612^ab^	0.707^a^	0.607^a^	0.480^a^
A + D+AD	0.805^a^	0.738^ab^	0.583 ^cd^	0.708^a^	0.600^a^	0.466^a^
A + D+AA + AD	0.805^a^	0.742^ab^	0.607^ab^	0.705^a^	0.604^a^	0.475^a^
RKHS	0.802^a^	0.735^b^	0.602^abc^	0.699^a^	0.599^a^	0.470^a^

Models	Environment: PI.IN			Environment: PI.LN		
	EH	PH	GY	EH	PH	GY
A	0.791^a^	0.701^a^	0.527^a^	0.800^a^	0.676^b^	0.456^b^
A + D	*0.797* ^a^	*0.720* ^a^	*0.543* ^a^	*0.806* ^a^	*0.699* ^a^	*0.487* ^a^
A + AA	0.792^a^	0.706^a^	0.534^a^	0.799^a^	0.685^ab^	0.476^ab^
A + AD	0.790^a^	0.702^a^	0.526^a^	0.799^a^	0.677^b^	0.457^b^
A + D+AA	0.794^a^	0.714^a^	0.54^a^	0.803^a^	0.694^ab^	0.483^a^
A + D+AD	0.794^a^	0.705^a^	0.537^a^	0.803^a^	0.679^b^	0.475^ab^
A + D+AA + AD	0.793^a^	0.712^a^	0.539^a^	0.803^a^	0.690^ab^	0.483^a^
RKHS	0.791^a^	0.708^a^	0.539^a^	0.802^a^	0.683^ab^	0.484^a^

*A* additive, *D* dominance, *AA* additive × additive, *AD* additive × dominance effects, *RKHS* reproducing kernel Hilbert spaces model, *AN.IN* Anhembi ideal nitrogen regime, *AN.LN* Anhembi low nitrogen regime, *PI.IN* Piracicaba ideal nitrogen regime, *PI.LN* Piracicaba low nitrogen regime. *EH*, *GY*, and *PH* ear height, grain yield, and plant height, respectively. Different letters indicate statistically significant differences at 0.05 significance level according to Tuckey’s Honest Significance Difference test. Italics numbers indicates the highest estimates

Overall, the differences in the prediction accuracy achieved through different models were moderate. For instance, for EH almost no differences were observed in prediction accuracy between models. However, for GY and PH, there was a clear superiority of models including dominance and the RKHS regression relative to the additive model.

**Fig. 5:**
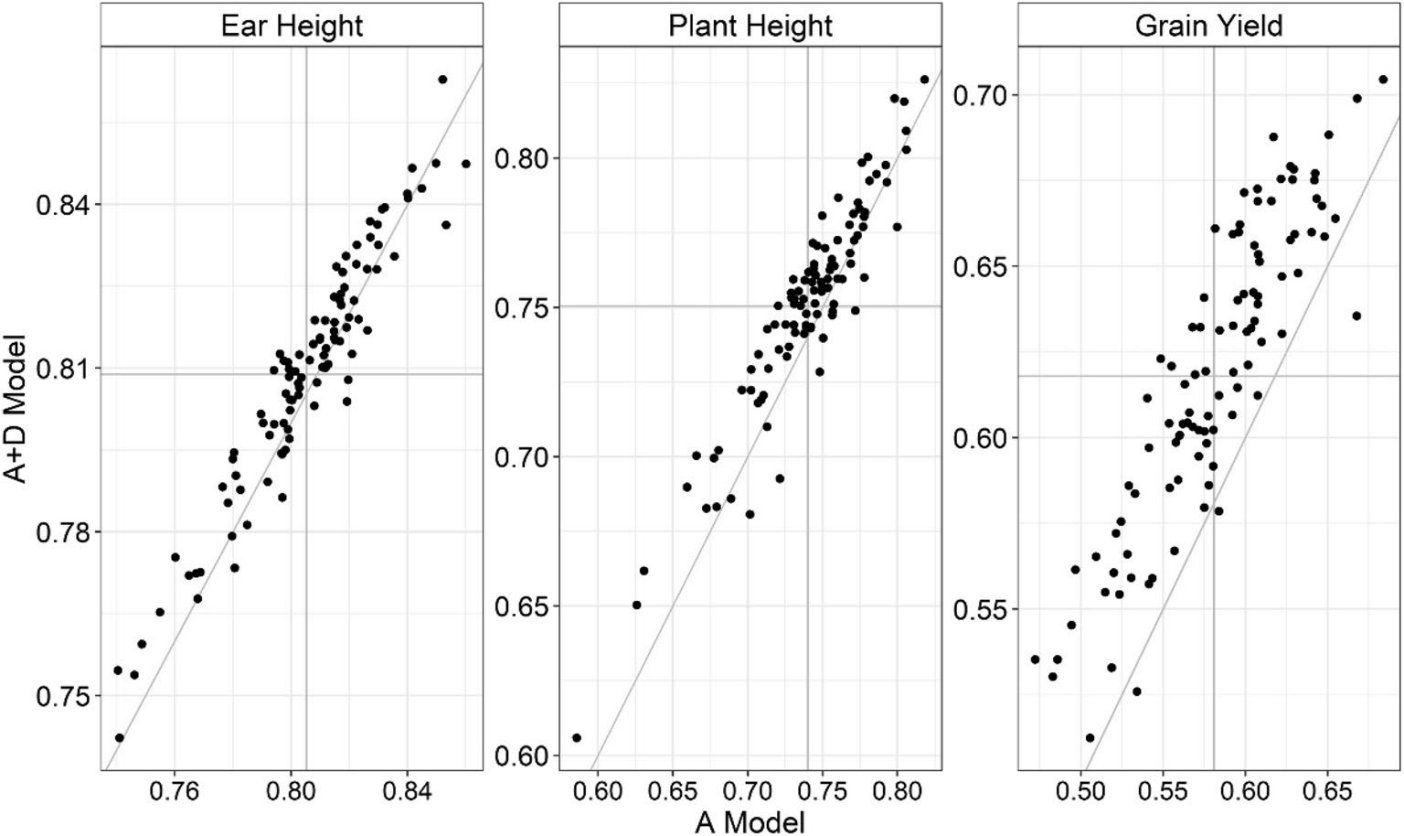
Scatter plot of the predictive accuracies obtained by the A + D model (Additive-dominance) and A (Additive) model by trait at Anhembi with ideal nitrogen availability (AN.IN). Each point represents one TRN-TST partition. The same population partitions were considered across models

For GY, the superiority of the A + D model over the A model was consistent across the validation sets in more than 95 of the 100 sets conducted the A + D model gave higher prediction correlation than the A model (Fig. 5). On the other hand, the proportion of times that the A + D model outperformed the A model was much more modest for EH. Plant height was in an intermediate situation, where the A + D model was in average better than the A model, but the superiority was not as consistent across CV as observed for GY. The same trend was observed in other environments (Additional file 8: Fig. S4).

### Predicting hybrid performance for observed and un-observed crosses

**Fig. 6:**
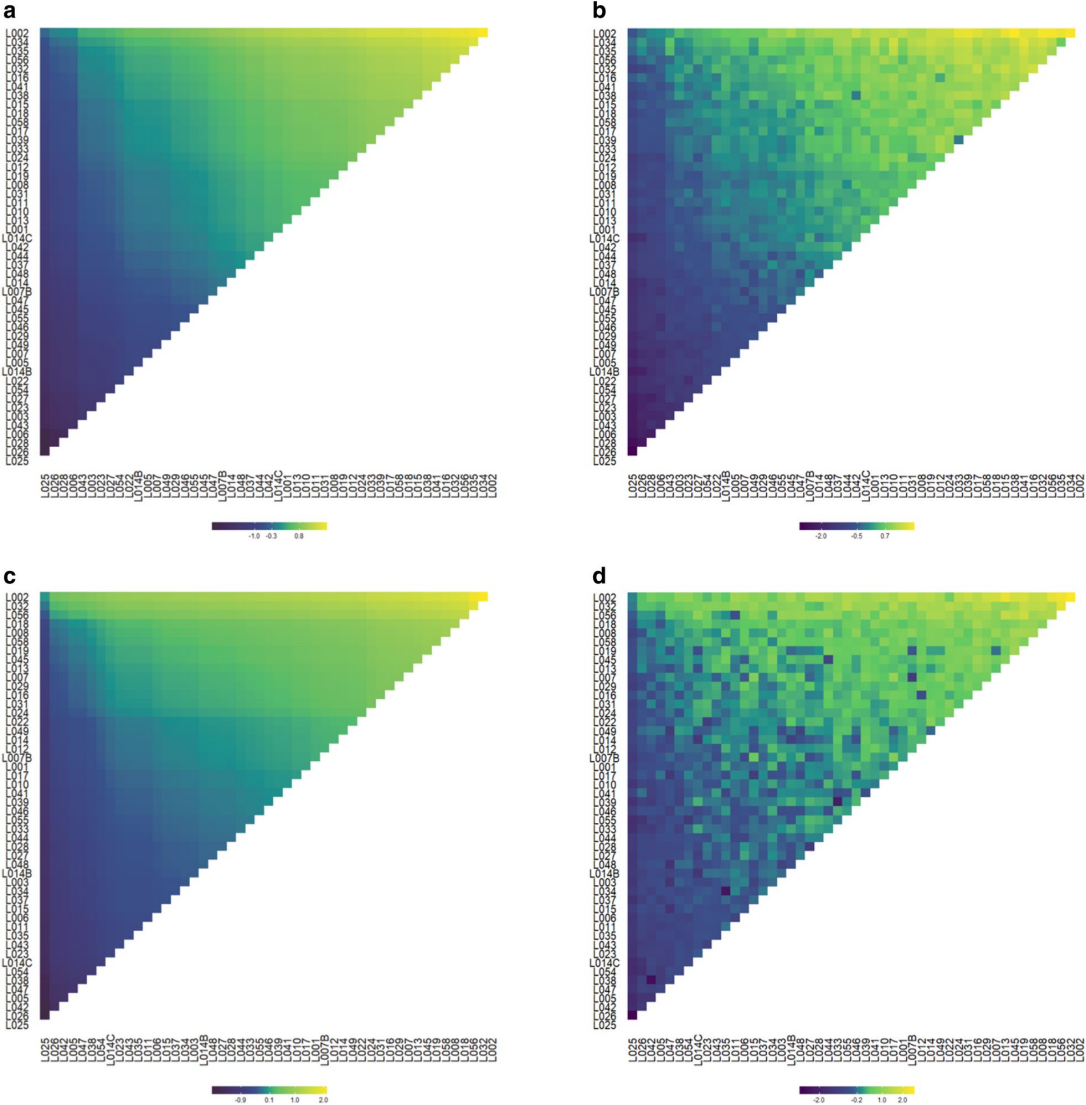
Heatmaps of genomic estimated genetic/genotypic values of all possible single-crosses at Anhembi with ideal nitrogen (AN.IN). **a**, **b** Ear height predicted using the additive and additive-dominance models; (**c**, **d**) Grain yield predicted using the additive and additive-dominance models. Lines and columns of each plot were sorted by the mean performance of parental inbred lines at all crosses considering the predicted values from the Additive model

We used the fitted models to predict the total genetic value of all possible hybrids that can be obtained from the 49 inbred lines available (Fig. 6, Additional files 9, 10 and 11: Figs. S5, S6, S7). In the heatmaps, the parental lines were sorted according to the mean predicted genetic values of its progeny (obtained from the additive model). Thus, variation on axis X (and Y) are due to general combining ability. This sorting of parental lines leads to a relatively smooth increase on predicted genotypic values along the diagonal of the heatmap (values increase in “top-right” direction). However, when dominance was included the patterns in the heatmaps were less smooth (this is particularly clear for grain yield, Fig. 6c, d). Overall, the best crosses that one would choose using an additive model (i.e., those in the top-right corner of each plot) are also predicted to have high genotypic value under the non-additive model. Nevertheless, for grain yield, there are also a few cases where the additive model predicts intermediate genetic values (points in the center of the heat maps) and the non-additive model predicts a higher genotypic value (this corresponds to yellow-green points in the center of the heat map).

## Discussion

In crops for which commercial genotypes are inbred lines (e.g., wheat, soybeans), F_1_ seeds (e.g., hybrids in maize, sunflower), or clones (e.g., potato, cassava, sugarcane, eucalyptus), advantageous gene combinations can be fixed and multiplied. In these cases, non-additive effects can be effectively exploited and maintained [32]. However, identifying the best genotypes requires extensive field evaluations, especially for F_1_ hybrids. Unfortunately, even for a small number of parental lines, evaluating all possible crosses in field trials becomes economically expensive and logistically complex.

Genetic similarity (derived from either pedigrees or molecular markers) can be leveraged to induce borrowing of information between crosses, and this can be used for prediction of performance of un-tested crosses [18, 24, 52]. For single crosses, hybrids’ genotypes can be derived from parental genotypes; thus, there is no additional genotyping cost involved when predicting yet-to-be developed hybrids. Moreover, the computational cost involved to predict the performance of untested crosses is minimal since most of the computational burden is on fitting the models and this is only done using data from tested hybrids.

In genomic models, the strength of borrowing information between hybrids depends on two main factors: the genetic similarity among the inbred lines and on the mode of gene action. Additive effects give rise to a smooth surface where the expected performance of a hybrid is the average of the general combining ability of the two parental lines (Fig. 1). Deviations from this plane can be accommodated using parametric models for non-additive effects (e.g., dominance or epistatic interactions) or using semi-parametric procedures. All these models can be formulated as multi-kernel regressions (e.g., [35, 53]), where different kernels are used to model different types of effects.

In this study, we demonstrate how Bayesian multi-kernel methods can be used to estimate the total genetic variance and its components (GCA and SCA) and to derive predictions of un-tested hybrids under parametric and semi-parametric assumptions. For simplicity, we fitted models within environments; however, the same methodology can be extended to multi-environments settings using models for marker-by-environment interactions such as those described in [54]. The application of these methods to hybrids generated by crossing inbred tropical maize lines from a convergent population lead to important conclusions that we highlight next.

*Additive effects dominate but “one*-*size*-*(does not)*-*fit*-*all” traits/populations* For the three traits analyzed, additive effects explained the majority of the genetic variance. The estimated variance components indicate that the analyzed traits have very different genetic architectures. EH showed the highest broad-sense heritability (~ 0.8), and a high proportion of genetic variance explained by additive effects ($D^{2}$ was only ~ 0.12). On the other hand, GY showed moderate broad-sense heritability (~ 0.5–0.6 for models including non-additive effects) and a sizable fraction of the total heritability accounted for non-additive effects ($D^{2}$ of 0.3–0.48). Regarding heritability and the relative importance of additive effects, PH represented an intermediate situation between EH and GY. In this respect, our results are in agreement with those reported by several authors [36, 55–57] who have indicated that additive effects explain a very large fraction of genetic variance for EH and PH and a smaller fraction of the genetic variance of GY.

Our estimates $\sigma _{GCA}^{2}$ and $\sigma _{SCA}^{2}$ estimates suggest that for PH and EH hybrid prediction/selection based only on additive effects should be effective [26, 27]. These results have important implications for the breeding process as they indicate that selection in early stages of the breeding process based on additive models should result in sizable changes in PH and EH at the hybrid level. In the case of GY accounting for non-additive effects appeared to be more critical, and our results indicate that using a model that accounts for additive and dominance can give as good, and sometimes higher, prediction accuracy than the one achieved by more complex models that accounted for epistatic interactions.

*Genetic diversity affects the ratio of SCA to GCA* Previous studies have shown that genetic divergence between inbred lines affects the $SCA_{ratio}$ [22, 29, 58]. Empirical evidence suggests that the $SCA_{ratio}$ is higher for hybrids originated by crossing materials from genetically homogeneous pools (i.e., sets of inbred lines with similar allele frequencies) than for very divergent goups [29, 59, 60]. Most of the published studies on maize hybrids are based on data originated by crossing lines from different heterotic groups [18, 22, 24, 31, 38]. Averaged across models and environments, our estimates of the $SCA_{ratio}$ were 0.14, 0.28, and 0.56 for EH, PH, and GY, respectively. These estimates are higher than what has been previously reported in genomic based studies for GY and PH [18, 25, 38]. We attribute these higher observed estimates to the genetic composition of our parental lines, all of which originated from the same convergent population that has very low levels of structure (see Additional file 12: Fig. S8).

*Analyses and prediction of more complex hybrids* Our results are entirely based on single cross hybrids. While the methodology described in this study can be used to analyze three- and four-way crosses, our empirical results cannot be extrapolated to more complex hybrids because the relative importance of additive and non-additive effects is expected to be different for more complex hybrids. Moreover, it is worth noting that the genotyping scheme needed to predict the performance of three- and four-way hybrids is more complex and expensive than the one used here.

Recently, Li et al. [61] developed a model that accounts for general and subpopulation-specific additive effects as well as dominance deviations for analysis and prediction of 3-way crosses. The ideas presented in this study for estimation of the total genetic variance and its components (GCA and SCA) can be easily applied to the model proposed by Li et al. [61].

*Non*-*additive effects appeared to be more important under nitrogen stress conditions* In all but one of the trait-by-environment combinations analyzed (PH in PI), the environments under low nitrogen regime showed higher $SCA_{ratio}$ than those under ideal nitrogen (Additional file 7: Table S6). Similar results were reported by [62] and [63], who concluded that for grain yield in maize non-additive variation appears to be more important in low nitrogen growing conditions. They also reported the higher importance of non-additive effects under drought stress. Our results agree with those findings, especially for GY. Therefore, overall, it seems that accounting for non-additive effects becomes paritcularly important under nitrogen stress conditions.

*Within model prediction accuracy was linearly related to heritability* Our results indicate that GP of hybrid performance can achieve a moderately high prediction accuracy. Also, for any given model, there was a direct relationship between the proportion of variance explained by the model (*H*^*2*^*)* and the prediction accuracy achieved in the prediction of un-tested hybrids. Interestingly, within the model, this relationship was very close to linear (Additional files 13 and 14: Figs. S9 and S10). For instance, prediction accuracy was highest (~ 0.8) for EH (the most heritable of the three traits analyzed), intermediate for PH (~ 0.7) and lowest (~ 0.5) for GY. Likewise, environments with lower heritability (those under stress conditions), were the one with lower prediction accuracy either. These results agree with the theoretical and empirical evidence, which support a direct relationship between trait heritability and prediction accuracy (e.g., [64, 65]).

*However, the models that fitted the data better were not always the ones that gave the highest prediction accuracy* Indeed, the relationship between the proportion of variance explained and prediction accuracy was not linear when comparing (within a trait or environment) results across models (Additional file 14: Fig. S10). For instance, while the RKHS model was in all cases the one that had the highest proportion of variance explained (Fig. 3a) the predictive performance of this model was not the highest one. *Overall, the best performing model across traits and environments was the A + D model* (Table 2 and Fig. 3). Models including two or more non-additive (e.g., A + D+AA + AD) terms fitted the data better but showed poorer predictive performance than the A + D model. It seems that the A + D model offers, at least for the sample size considered here, a good balance between goodness of fit and model complexity.

*Alternative parameterizations for the A *+ *D model did not improve the model performance* In the analysis of hybrids originated by crossing lines from two or more heterotic groups models often account for group-specific additive variances [18, 22, 24, 38, 52]. This was not needed in our case because all the inbreed lines originated from the same population. However, for sensitivity analyses we compared the performance of the A + D model used in our study with two other models: (1) one accounting for group-specific additive variance (A1 + A2 + D, where A1 and A2 are the female and male additive effects, respectively); and (2) one in which, as suggested by Martini et al. [66], we use three dummy variables per locus (one for each possible genotype, labeled as categorical model). The A1 + A2 + D and the categorical model use three effects per locus, while the A + D uses two effects per locus. From this perspective, the A1 + A2 + D and categorical models are more “flexible” than the A + D model. However, when the categorical model is implemented using a single variance parameter this may reduce the ability of the model to fit the data. In our study, this did not happen once both the A1 + A2 + D and the categorical model fitted the data slightly better than the A + D model (Additional file 15: Table S7). However, none of these models had better predictive performance than the simpler A + D model (Additional file 15: Table S7). Our results are similar to Technow et al. [22] findings, and suggest that for hybrids originated from crosses among lines of convergent populations the two degrees of freedom parameterization for A + D effects is sufficient to achieve high prediction accuracies.

**Fig. 7:**
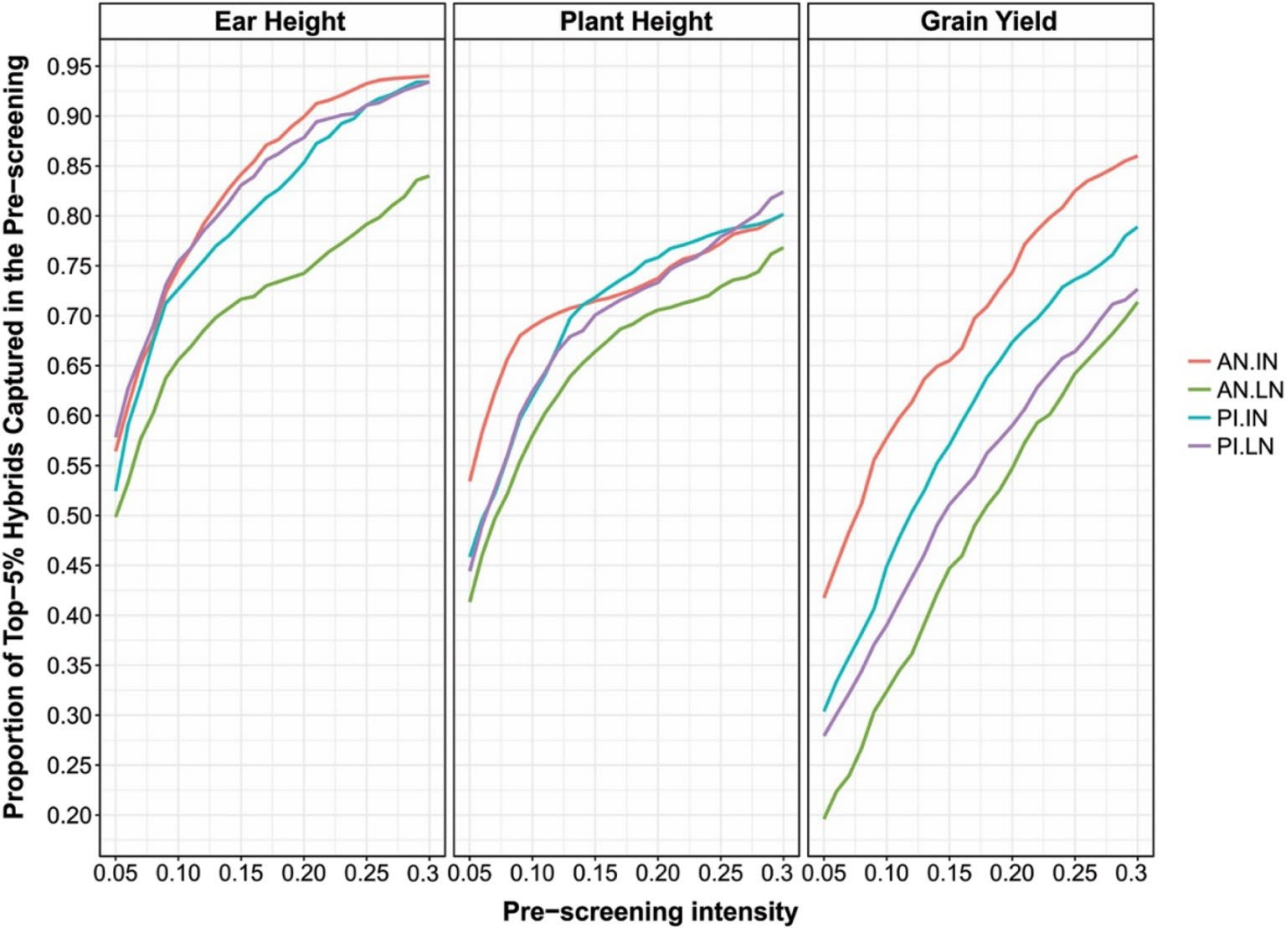
Proportion of the top-5% hybrids (according to phenotypic rank) that is identified by pre-screening based on (cross-validation) genomic prediction using the additive + dominance model at a different intensity of selection (x-axis). Each panel corresponds to an evaluated trait, lines within a plot represent different environments. AN: Anhembi; PI: Piracicaba; LN: Low nitrogen; IN: Ideal nitrogen

*Genomic prediction can be effectively used for pre-screening* GP could be used to select a subset of promising hybrids which can later be tested at field evaluations. This approach can significantly reduce the time and costs involved in generating hybrids and could reduce the probability that superior hybrids do not reach the field testing stage [18, 67, 68]. To assess the accuracy of pre-screening based on GP we estimated, using cross-validation predictions, the proportion of the top-5% of the hybrids (from the ranking based on the observed trait) that is captured within a set of hybrids selected using genomic prediction (Fig. 7). Selecting the best 30% of the crosses based on genomic prediction leads to a subset of hybrids that contained between 85 and 95% of the top-5% of hybrids with the highest ear height. For PH, the best 30% of the hybrids in the genomic screening contained between 70 and 80% of the top-5% best hybrids. Finally, the set containing the 30% of the hybrids with the highest genomic prediction values for GY included between 70 and 85% of the hybrids with highest GY in field evaluations. These results are in agreement with [69], who found high concordance among superior wheat lines selected by GS and phenotypic selection from multi-environment trials. We conclude that pre-screening using GP, coupled with field testing of top hybrids pre-selected using GP, can be an effective approach for incorporating GP into hybrid selection programs.

**Fig. 8:**
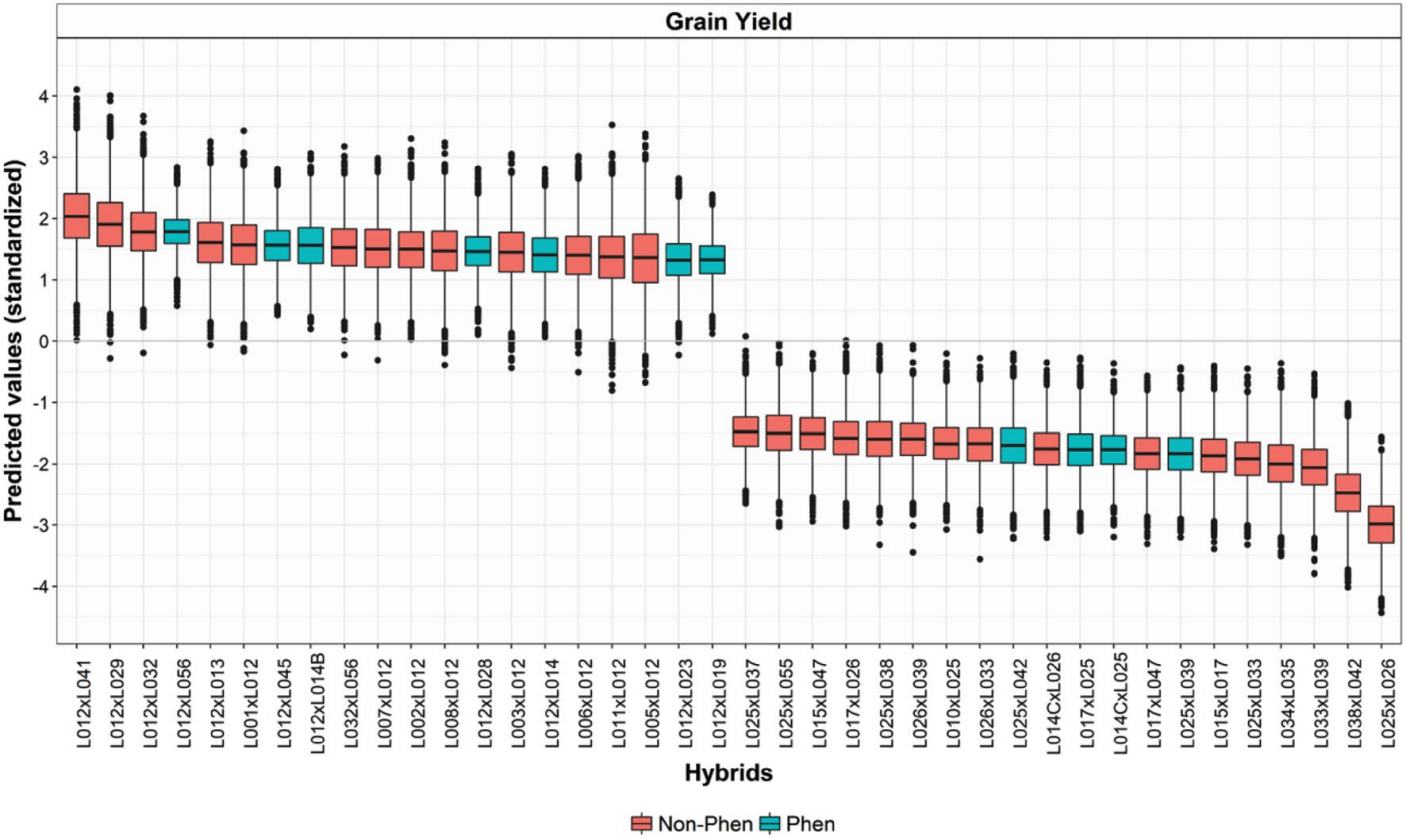
Boxplots of the posterior distribution of expected hybrid performance for the top- and lowest-20 ranked hybrids (left and right, respectively) for grain yield at Anhembi under an optimum nitrogen regime. The colors of the boxes indicate whether the hybrid was phenotyped or not (see legend), the label in the axis indicates the parental lines

*Using Posterior distributions to summarize uncertainty about hybrid predictions* All the results presented and discussed so far were based on the estimated posterior means of hybrid performance. However, the posterior distribution from Bayesian models can also be used to assess the uncertainty about predicted hybrid performances. Importantly, unlike likelihood methods, posterior distributions from Bayesian models fully accounts from all sources of uncertainty, including uncertainty about variance parameters [70]. Figure 8 display the estimated posterior distribution of hybrid performance for the top- and lowest-20 ranked hybrids (according to the posterior mean of hybrid performance from the A + D model) for GY in the Anhembi under ideal nitrogen regime environment (AN.IN, see Additional files 16, 17, 18, and 19: Figs. S11, S12, S13, and S14 for other traits and environments). In almost all cases the top-20 hybrids had a posterior distribution that contains at least 75% of the mass over one standard deviation of the mean performance. The blue boxplots correspond to hybrids that were tested in field trials, and the red ones are for untested hybrids. In all cases, the top-20 hybrids contained many untested genotypes. As one would expect, the spread of the posterior distribution was higher for un-tested hybrids.

## Conclusions

Genomic Prediction models can be used to predict the performance of tested and untested hybrids, and those predictions can be used to decide which hybrids should be further tested in field trials. We review Bayesian parametric and non-parametric models for additive and non-additive effects. All the methods discussed in this study can be cast as kernel regressions. We show how Gaussian kernels for non-parametric models can be derived from additive relationship matrices. All the kernel regressions discussed in this study can be implemented in either a Likelihood or Bayesian framework. However, Bayesian models offer a great deal of flexibility and a framework that allows evaluating uncertainty about variance components and hybrid performance that fully account for all sources of uncertainty. We discussed how samples from the posterior distribution can be used to estimate the total genetic variance and its components (SCA and GCA) while accounting for covariances between additive and non-additive effects. We also discuss how samples collected Monte Carlo Markov Chain algorithms can be used to fully assess the posterior distribution of predicted hybrid performance. Our results show that that non-additive effects play an important role in the expression of traits such as grain yield and suggest that the relative importance of non-additive effects is higher, under nitrogen stress conditions. In all the traits and environments considered, the A + D model achieve either the best or very close to the best, predictive performance.

## Additional files

**Additional file 1.** **Supplementary Methods 1 and 2:** In **Supplementary Method 1** we show that Hadamard products of additive relationship matrices provide a covariance structure that represents not only additive-by-additive contrasts but also dominance and provides a straightforward method to construct a kernel comprising only additive by additive epistasis effects. **Supplementary Method 2** shows how to compute Gaussian kernels based on additive relationship matrices.

**Additional file 2 .** **R-Scripts:** In this additional file we show (summary of the) R scripts used to generate the results presented in the study.

**Additional file 3.** **Fig. S1:** Number of crosses in which each one of the 49 inbred lines occurred.

**Additional file 4.** **Fig. S2:** Boxplots of the pairwise linkage disequilibrium (*r*^2^) by distance (Kbp) for chromosomes 1 to 10 in 49 inbred lines of tropical maize. Red dots and black traces represent means and medians, respectively. Each box represents the *r*^*2*^ estimates inside the first and third quartiles (25 and 75% percentiles, respectively).

**Additional file 5.** **Tables S1, S2, S3, S4, and S5:** Posterior means of variance parameters and broad-sense genomic heritability (SD) by traits and models in different environments.

**Additional file 6.** **Fig. S3:** Posterior density of the covariance between the additive and nonadditive genetic components by environment and traits. **a** A + D+AA model. **b** A + D+AD model. **c** A + D+AA + AD model. Covariance between A and D effects is represented in green ($\upsigma_{\text{a}}^{2}$–$\upsigma_{\text{d}}^{2}$); Covariance between A and AA is represented in red ($\upsigma_{\text{a}}^{2}$–$\upsigma_{\text{aa}}^{2}$); Covariance between A and AD is represented in blue ($\upsigma_{\text{a}}^{2}$–$\upsigma_{\text{ad}}^{2}$). Covariance between D and AA is represented in golden ($\upsigma_{\text{d}}^{2}$–$\upsigma_{\text{aa}}^{2}$); Covariance between D and AD is represented in blue ($\upsigma_{\text{d}}^{2}$–$\upsigma_{\text{ad}}^{2}$). AN: Anhembi, PI: Piracicaba, IN: Ideal nitrogen, and LN: Low nitrogen. A: Additive effect, and D: Dominance effects. A: Additive effect, D: Dominance, AA: Additive-additive, and AD: Additive-dominance effects.

**Additional file 7.** **Table S6:** Ratio between non-additive and additive variances ($SCA_{ratio}$) and standard deviation by trait, environment, and model.

**Additional file 8.** **Fig. S4:** Scatter plots of the prediction accuracies obtained by the A + D model (Additive-dominance) and A (Additive) model by trait. **a:** AN.LN: Anhembi low nitrogen availability; **b:** PI.IN: Piracicaba ideal nitrogen availability; **c:** PI.LN: Piracicaba low nitrogen availability. Each point represents one TRN-TST partition. The same population partitions across models were considered.

**Additional file 9.** **Fig. S5:** Heatmaps of predicted values of all possible single-crosses hybrids at Piracicaba with low nitrogen availability (AN.LN). **a, b** Ear height predicted using the additive and additive-dominance models; **c, d** Grain yield predicted using the additive and additive-dominance models. Lines and columns of each plot were sorted by the mean performance of parental inbred lines at all crosses considering the predicted values predicted in the Additive model.

**Additional file 10.** **Fig. S6:** Heatmaps of predicted values of all possible single-crosses hybrids at Piracicaba with ideal nitrogen availability (PI.IN). **a**, **b** Ear height predicted using the additive and additive-dominance models; **c**, **d** Grain yield predicted using the additive and additive-dominance models. Lines and columns of each plot were sorted by the mean performance of parental inbred lines at all crosses considering the predicted values predicted in the Additive model.

**Additional file 11.** **Fig. S7:** Heatmaps of predicted values of all possible single-crosses hybrids at Piracicaba with low nitrogen availability PI.LN. **a**, **b** Ear height predicted using the additive and additive-dominance models; **c**, **d** Grain yield predicted using the additive and additive-dominance models. Lines and columns of each plot were sorted by the mean performance of parental inbred lines at all crosses considering the predicted values predicted in the Additive model.

**Additional file 12.** **Fig. S8:** Population structure of 49 inbred lines in maize. **a** Cumulative proportion of variance explained by the principal components (PC). **b** PC2 versus PC1. **c** PC3 versus PC1. **d** PC4 versus PC1. **e** PC3 versus PC2.

**Additional file 13.** **Fig. S9:** Linearity among prediction accuracy ($r_{{y\hat{y}}}$) and broad-sense genomic heritability ($H^{2}$) by trait/environment within prediction model. EH: Ear height, PH: Plant height, and GY: Grain yield. AN: Anhembi, PI: Piracicaba, IN: Ideal nitrogen, and LN: Low nitrogen. A: Additive effect, and D: Dominance effects

**Additional file 14.** **Fig. S10:** Cross-validation prediction accuracy ($r_{yy}^{{}}$) versus broad-sense genomic heritability ($H^{2}$) by trait, environment, and model. A: Additive, D: Dominance, AA: Additive x additive, and AD: Additive x dominance effects. RKHS: Reproducing Kernel Hilbert Spaces model). AN: Anhembi, PI: Piracicaba, LN: Low nitrogen, IN: Ideal nitrogen.

**Additional file 15.** **Tables S7** and **S8**: Comparison of posterior means of variance parameters and broad-sense genomic heritability (SD), and prediction accuracies by trait, models (A + D, 3-DF, and A1 + A2 + D), and environments.

**Additional file 16.** **Fig. S11**: Boxplots of the posterior distribution of expected hybrid performance for the top- and lowest-20 ranked hybrids (left and right, respectively) for ear and plant height at AN.IN. The colors of the boxes indicate whether the hybrid was phenotyped or not (see legend), the label in the axis indicates the parental lines. AN: Anhembi; IN: Ideal nitrogen regime.

**Additional file 17.** **Fig. S12**: Boxplots of the posterior distribution of expected hybrid performance for the top- and lowest-20 ranked hybrids (left and right, respectively) for ear height, grain yield, and plant height at AN.LN. The colors of the boxes indicate whether the hybrid was phenotyped or not (see legend), the label in the axis indicates the parental lines. AN: Anhembi; LN: Low nitrogen regime.

**Additional file 18.** **Fig. S13**: Boxplots of the posterior distribution of expected hybrid performance for the top- and lowest-20 ranked hybrids (left and right, respectively) for ear height, grain yield, and plant height at PI.IN. The colors of the boxes indicate whether the hybrid was phenotyped or not (see legend), the label in the axis indicates the parental lines. PI: Piracicaba; IN: Ideal nitrogen regime.

**Additional file 19.** **Fig. S14**: Boxplots of the posterior distribution of expected hybrid performance for the top- and lowest-20 ranked hybrids (left and right, respectively) for ear height, grain yield, and plant height at PI.LN. The colors of the boxes indicate whether the hybrid was phenotyped or not (see legend), the label in the axis indicates the parental lines. PI: Piracicaba; LN: Low nitrogen regime.

## Abbreviations

GP: genomic prediction
EH: ear height
PH: plant height
GY: grain yield
A: additive
D: dominance
AA: additive × additive
AD: additive × dominance effects
RKHS: reproducing Kernel Hilbert Spaces model
AN.IN: anhembi ideal nitrogen regime
AN.LN: Anhembi low nitrogen regime
PI.IN: Piracicaba ideal nitrogen regime
PI.LN: Piracicaba low nitrogen regime

## Footnotes

1 For simplicity we assume that all the phenotypes are expressed as deviations from the overall mean. Thus, all the terms including g_*ij*_, *u*_*i*_ and *u*_*j*_ are assumed to have null mean.
