# Supplementary material for: Bayesian analysis and prediction of hybrid performance
Source: Plant Methods. 2019 Feb 7;15:14. doi: 10.1186/s13007-019-0388-x (PMC6366084; doi:10.1186/s13007-019-0388-x)
Supplement: Supplementary file 1 — Additional file 1. Supplementary Methods 1 and 2: In Supplementary Method 1 we show that Hadamard products of additive relationship matrices provide a covariance structure that represents not only additive-by-additive contrasts but also dominance and provides a straightforward method to construct a kernel comprising only additive by additive epistasis effects. Supplementary Method 2 shows how to compute Gaussian kernels based on additive relationship matrices. [file 13007_2019_388_MOESM1_ESM.docx]

**Supplementary Method 1: Constructing kernels for additive-by-additive epistatic interactions**

In this section, we show that Hadamard products of additive relationship matrices provide a covariance structure that represents not only additive-by-additive contrasts but also dominance. We then describe a simple way to remove the contribution to dominance. We use this approach in our study to model additive-by-additive epistasis.

**Hadamard products and covariance matrices for interactions: general case.**

Let $\boldsymbol{X}_{n\times p}$ and $\boldsymbol{Z}_{n\times q}$ represent two incidence matrices of effects and consider modelling all possible first order interactions between the columns of $\boldsymbol{X}$ and $\boldsymbol{Z}$. Here, $\boldsymbol{X}$ and $\boldsymbol{Z}$ may: (i) represent different information sets (e.g., $\boldsymbol{X}$ may be a matrix with SNP genotypes and $\boldsymbol{Z}$ may be a matrix with environmental covariates, as in Jarquin *et al.* [1]), (ii) be different types of contrasts derived from the same information set (e.g., $\boldsymbol{X}$ may code additive effects and $\boldsymbol{Z}$ may provide contrasts for dominance) or, (iii) $\boldsymbol{Z=X}$ (this will be the case when modeling additive-by-additive interactions).

The incidence matrix containing contrasts for all possible interactions between the columns of$\boldsymbol{X}$ and $\boldsymbol{Z}$ can be formed using Kronecker products of the rows of $\boldsymbol{X}$ and $\boldsymbol{Z}$. Specifically,

$\boldsymbol{W=}\left[ \begin{matrix} \boldsymbol{x}_{1}^{'}\bigotimes\boldsymbol{z}_{1}^{'} \\ \begin{matrix} \boldsymbol{x}_{2}^{'}\bigotimes\boldsymbol{z}_{2}^{'} \\ \vdots\end{matrix} \\ \boldsymbol{x}_{n}^{'}\bigotimes\boldsymbol{z}_{n}^{'} \end{matrix} \right]$, [1]

where $\boldsymbol{x}_{i}^{'}$ and $\boldsymbol{z}_{i}^{'}$ are the i^th^ rows of matrices $\boldsymbol{X}$ and $\boldsymbol{Z}$, respectively, is a matrix containing interactions. Indeed, the Kronecker products entering in the righ-hand side of [1], $\boldsymbol{x}_{i}^{'}\bigotimes\boldsymbol{z}_{i}^{'}=\left[ x_{i1}z_{i1},x_{i1}z_{i2},\ldots,x_{i1}z_{iq},x_{i2}z_{i1},\ldots x_{ip}z_{iq-1},x_{ip}z_{iq} \right]$**,** generate all possible interactions beteween the colums of $\boldsymbol{X}$ and $\boldsymbol{Z}$.

A covariance structure for a linear model for $\boldsymbol{W}$ takes the form

$\boldsymbol{K=}\boldsymbol{W}\boldsymbol{W}^{\boldsymbol{'}}\boldsymbol{=}\left[ \begin{matrix} \boldsymbol{x}_{1}^{'}\bigotimes\boldsymbol{z}_{1}^{'} \\ \begin{matrix} \boldsymbol{x}_{2}^{'}\bigotimes\boldsymbol{z}_{2}^{'} \\ \vdots\end{matrix} \\ \boldsymbol{x}_{n}^{'}\bigotimes\boldsymbol{z}_{n}^{'} \end{matrix} \right]\left[ \begin{matrix} \left( \boldsymbol{x}_{1}^{'}\bigotimes\boldsymbol{z}_{1}^{'} \right)^{'}, & \ldots& {,\left( \boldsymbol{x}_{n}^{'}\bigotimes\boldsymbol{z}_{n}^{'} \right)}^{'} \end{matrix} \right]$ [2]

The *ij^th^* element of this kernel is

$\boldsymbol{K}_{ij}\boldsymbol{=}\left( \boldsymbol{x}_{i}^{'}\bigotimes\boldsymbol{z}_{i}^{'} \right)\left( \boldsymbol{x}_{j}^{'}\bigotimes\boldsymbol{z}_{j}^{'} \right)^{'}=\left( \boldsymbol{x}_{i}^{'}\bigotimes\boldsymbol{z}_{i}^{'} \right)\left( \boldsymbol{x}_{j}\bigotimes\boldsymbol{z}_{j} \right)=\left( \boldsymbol{x}_{i}^{'}\boldsymbol{x}_{j}\bigotimes\boldsymbol{z}_{i}^{'}\boldsymbol{z}_{j} \right)\boldsymbol{=}\boldsymbol{x}_{i}^{'}\boldsymbol{x}_{j}\times\boldsymbol{z}_{i}^{'}\boldsymbol{z}_{j}$. [3]

This entry is equal to the *ij^th^* element of the Hadamard product $\boldsymbol{X}\boldsymbol{X}^{\boldsymbol{'}}\boldsymbol{\odot Z}\boldsymbol{Z}^{\boldsymbol{'}}$. Indeed the *ij^th^* element of this product is the product of the *ij^th^* element of $\boldsymbol{X}\boldsymbol{X}^{\boldsymbol{'}}$**,** $\boldsymbol{X}\boldsymbol{X}_{ij}^{\boldsymbol{'}}\boldsymbol{=}\boldsymbol{x}_{i}^{'}\boldsymbol{x}_{j}$, times the *ij^th^* element of $\boldsymbol{Z}\boldsymbol{Z}^{\boldsymbol{'}}$, $\boldsymbol{Z}\boldsymbol{Z}_{ij}^{\boldsymbol{'}}\boldsymbol{=}\boldsymbol{z}_{i}^{'}\boldsymbol{z}_{j}$. Therefore

$\boldsymbol{X}\boldsymbol{X}^{\boldsymbol{'}}\boldsymbol{\odot Z}\boldsymbol{Z}^{\boldsymbol{'}}\boldsymbol{=W}\boldsymbol{W}^{\boldsymbol{'}}$. [4]

The result in [4] shows that a covariance structure for all possible interactions between the predictors included in $\boldsymbol{X}$ and $\boldsymbol{Z}$ can be computed by first computing a kernel for each of these matrices, $\boldsymbol{K}_{X}\boldsymbol{=X}\boldsymbol{X}^{\boldsymbol{'}}$ and $\boldsymbol{K}_{Z}\boldsymbol{=Z}\boldsymbol{Z}^{\boldsymbol{'}}$**,** and then producing the Haddmard product between these two kernels $\boldsymbol{K}_{X}\boldsymbol{\odot}\boldsymbol{K}_{Z}$. This result was used, for example, by Jarquin *et al.* [1] to compute kernels for all possible interactions between SNPs and environmental covariates. The same result can be use to compute additive-by-dominance epistatic interactions, indeed, setting $\boldsymbol{X}$ and $\boldsymbol{Z}$ to be matrices with contrasts for additive and dominance effects (dentoed as ***D*** in our manuscript), respectively, and computing the expression in [4] renders a covariance structure additive-by-dominance epistatic interactions.

**Computing kernels for additive-by-additive epistatic interactions**

For additive-by-additive interactions, we need to set $\boldsymbol{Z}=\boldsymbol{X}$ with $\boldsymbol{X}$ being a matrix with contrasts for addtive effects. However, in this case, the interactions included in the columns of ***W*** (expression [1]) include not only interactions between loci but also interactions within loci, $x_{ij}x_{ij}=x_{ij}^{2}$, indeed, if $\boldsymbol{Z}=\boldsymbol{X}$,

$$\boldsymbol{x}_{i}^{'}\bigotimes\boldsymbol{x}_{i}^{'}\boldsymbol{=}\left[ x_{i1}^{2},x_{i1}x_{i2},\ldots,x_{i1}x_{ip},x_{i2}x_{1i},x_{i2}^{2},\ldots,x_{ip}^{2} \right]$$

The within-loci interactions correspond to dominance; therefore, computing the Hadamard product of additive relationship matrices, $\boldsymbol{X}\boldsymbol{X}^{\boldsymbol{'}}\boldsymbol{\odot X}\boldsymbol{X}^{\boldsymbol{'}}$ gives a kernel that accounts not only for additive-by-additive effects but also for dominance.

A simple way to remove the contribution to dominance is to set the kernel for additive by additive effects to be $\boldsymbol{K}_{aa}\boldsymbol{=X}\boldsymbol{X}^{\boldsymbol{'}}\boldsymbol{\odot X}\boldsymbol{X}^{\boldsymbol{'}}\boldsymbol{-}\left( \boldsymbol{X}\boldsymbol{\odot}\boldsymbol{X} \right)\left( \boldsymbol{X}\boldsymbol{\odot}\boldsymbol{X} \right)^{\boldsymbol{'}}$ where the second term represents a kernel for intra loci interactions. Substracting this from $\boldsymbol{X}\boldsymbol{X}^{\boldsymbol{'}}\boldsymbol{\odot X}\boldsymbol{X}^{\boldsymbol{'}}$ removes the contribution to dominance. In our application we further standardize this kernel to have an average diagonal value of one, therefore, for additive-by-additive effects we used:

$\boldsymbol{K}_{aa}=\frac{\left( \boldsymbol{X}\boldsymbol{X}^{\boldsymbol{'}} \right)\boldsymbol{\odot}\left( \boldsymbol{X}\boldsymbol{X}^{\boldsymbol{'}} \right)^{\boldsymbol{'}}\boldsymbol{-}\left( \boldsymbol{X}\boldsymbol{\odot}\boldsymbol{X} \right)\left( \boldsymbol{X}\boldsymbol{\odot}\boldsymbol{X} \right)^{\boldsymbol{'}}}{\frac{\boldsymbol{tr}\left( \left( \boldsymbol{X}\boldsymbol{X}^{\boldsymbol{'}} \right)\boldsymbol{\odot}\left( \boldsymbol{X}\boldsymbol{X}^{\boldsymbol{'}} \right)^{\boldsymbol{'}}\boldsymbol{-}\left( \boldsymbol{X}\boldsymbol{\odot}\boldsymbol{X} \right)\left( \boldsymbol{X}\boldsymbol{\odot}\boldsymbol{X} \right)^{\boldsymbol{'}} \right)}{\boldsymbol{n}}}$ [5]

**Supplementary Method 2: Computing gaussian kernels from additive relationship matrices**

Gaussian Kernels are often used in genomic kernel regressions. The *ij^th^* entry of a Gaussian kernel is computed as follows: $\boldsymbol{K}_{ij}=e^{-h\left\| \boldsymbol{x}_{i}-\boldsymbol{x}_{j} \right\|^{2}}$ . Here, $\left\| \boldsymbol{x}_{i}-\boldsymbol{x}_{j} \right\|=\sum_{k=1}^{p} \left( x_{ik}-x_{jk} \right)^{2}$ is the squared-Euclidean distance between the genotypes of the *i^th^* and *j^th^* individual and $h$ is a bandwidth parameter that controls how fast the kernel drop with the distance between genotypes. Determining the appropiate value of $h$ can be challenging and values of $h$ used in one study cannot be authomatically considered for other studies because the distance between genotypes depends on both how closely related the genotypes are but also on how many markers were used (because the distance is a sum over markers and it monotonically increases with the number of SNPs).

Additive relationship matrices can be used to derive squared-Euclidean distances and have an embedded standardization that makes the choice of the bandwidth parameter relatively straightforward. Therefore, Gaussian kernels can be directly computed from additive relationship matrices.

**Deriving squared-Euclidean distances from relationship matrices**

Kernels for linear models for additive effects are often computed using $\boldsymbol{K}_{a}=\boldsymbol{X}\boldsymbol{X}^{\boldsymbol{'}}$ where $\boldsymbol{X}$ is an incidence matrix for additive effect containing centered (and possibly-scaled) SNP genotypes. Additive relationship matrices provide a measure of genetic similarity between pairs of individuals. However, similarity matrices also define distances, more specifically, the distance betewen the *i* and *j* genotype is defined as

$D_{ij}=\boldsymbol{X}\boldsymbol{X}_{ii}^{\boldsymbol{'}}\boldsymbol{+X}\boldsymbol{X}_{jj}^{\boldsymbol{'}}\boldsymbol{-}2\boldsymbol{X}\boldsymbol{X}_{ij}^{\boldsymbol{'}}$. [6]

The *ij^th^* element of this kernel is $\boldsymbol{X}\boldsymbol{X}_{ij}^{\boldsymbol{'}}\boldsymbol{=}\boldsymbol{x}_{i}^{'}\boldsymbol{x}_{j}$; therefore, $D_{ij}=\boldsymbol{x}_{i}^{'}\boldsymbol{x}_{i}\boldsymbol{+}\boldsymbol{x}_{j}^{'}\boldsymbol{x}_{j}\boldsymbol{-}2\boldsymbol{x}_{i}^{'}\boldsymbol{x}_{j}=\sum_{k=1}^{p} x_{ik}^{2}+\sum_{k=1}^{p} x_{jk}^{2}-2\sum_{k=1}^{p} x_{ik}x_{jk}$. This quantidy is the squared-Euclidiean distance $\left\| \boldsymbol{x}_{i}-\boldsymbol{x}_{j} \right\|=\sum_{k=1}^{p} \left( x_{ik}-x_{jk} \right)^{2}=\sum_{k=1}^{p} x_{ik}^{2}+x_{jk}^{2}-2x_{ik}x_{jk}=\sum_{k=1}^{p} x_{ik}^{2}+\sum_{k=1}^{p} x_{jk}^{2}-2\sum_{k=1}^{p} x_{ik}x_{jk}$.

Therefore, squared-Euclidian distances can be directly computed from additive relationship matrices. These distances can then be used into a Gaussian kernel for semi-parametric regression.

**Bandwidth parameters**

If the additive relationship matrix was standardized (e.g., if it had, by construction, an average diagonal value of one) that standardization carries over the distances. Using distances computed from [6] with a bandwidth parameter equal to one leads to a kernel that has off-diagonal covariances similar to that of the original additive relationship matrix (Figure 1). Using higher values of the bandwidth parameter will lead to more a ‘local kernel’ which may be more in line with the covariance structures induce by epistatic terms.

Multi-kernel models (aka, “Kernel Averaging”, e.g., [2]) use kernels that represent different degrees of smoothness with respect to genotype differences. One possibility is to derive distances according to [6] and then derive three kernels, one with h=0.5 (this gives higher covariances than the additive relationship matrices), one with h=1 (this gives a kernel with covariances similar to that of the additive kernel, Figure 8) and one with h=2, which gives a kernel that is more local than the one representing additive relationship matrices. This is illustrated in the figure below. We use these three kernels in the study for our RKHS regression.

**Figure 1**: Entries of a Gaussian Kernel versus Additive Relationships, by the value of the bandwidth parameter.

**References**

1. Jarquín D, Crossa J, Lacaze X, Du Cheyron P, Daucourt J, Lorgeou J, et al. A reaction norm model for genomic selection using high-dimensional genomic and environmental data. Theor Appl Genet. 2014;127:595–607.

2. de los Campos G, Gianola D, Rosa GJM, Weigel KA, Crossa J. Semi-parametric genomic-enabled prediction of genetic values using reproducing kernel Hilbert spaces methods. Genet Res. 2010;92:295–308.
