# Supplementary material for: Bayesian analysis and prediction of hybrid performance
Source: Plant Methods. 2019 Feb 7;15:14. doi: 10.1186/s13007-019-0388-x (PMC6366084; doi:10.1186/s13007-019-0388-x)
Supplement: Supplementary file 2 — Additional file 2 . R-Scripts: In this additional file we show (summary of the) R scripts used to generate the results presented in the study. [file 13007_2019_388_MOESM2_ESM.html]

Scripts Used for Analyses


# Scripts Used for Analyses

#### *Filipe Couto Alves*

#### *10/03/2018*

## LIBRARIES

```
  library(BGLR)
```

## DATA

The dataset includes:

1. `y`:data frame containing the hybrids adjusted phenotypes
2. `Ga`, `Gd`,`Gaa`, and `Gad`: additive, dominance, additive-additive, and additive-dominance relationship matrices, respectively (computed based on the equations showed on the section “Parametric kernels for additive and non-additive effects”)

```
 load('Dataset.RData')
 head(y)
```

### Computing relationship matrices for semi-parametric models (presented on Additional File 1: Supplementary Method 2)

The following code shows how to derive genetic distances (`D`) based on the additive relationship matrix and how to estimate Gaussian kernels based on these distances.

```
 # Computin g distances
  n<-nrow(Ga)
  D<-matrix(0,ncol=ncol(Ga),nrow=nrow(Ga))
  for(i in 1:(n-1)){
    for(j in 1:n){
      D[i,j]<-Ga[i,i]+Ga[j,j]-2*Ga[i,j]
      D[j,i]<-D[i,j]
    }
  }

 # Kernels using h= (2,1,0.5)
 colnames(D)<-rownames(D)<-rownames(Ga)
 K1<-exp(-2*D)
 K2<-exp(-D)
 K3<-exp(-0.5*D)
```

## Decomposing the kernels into principal components

There are several ways to fit multi-kernel models in `BGLR` (see, examples here and here); we use principal components. All principal components matrices (`PCa`, `PCd`, `PCaa`, `PCad`) were derived as showed below:

```
 # Sorting matrices to match phenotypes
  ord<-match(unique(y$GID),rownames(Ga))
  Ga<-Ga[ord,ord]

 # Computing PCs
  EVDa=eigen(as.matrix(Ga),symmetric=T)
  PCa=EVDa$vectors[,EVDa$values>1e-5]    

 # Scaling
  for(i in 1:ncol(PCa)){ PCa[,i]=PCa[,i]*sqrt(EVDa$values[i])}
```

### Parameters for `BGLR` analyses

```
 nIter<-10000
 burnin<-1000
 thin=5
 Method<-"BRR"
 traits<-"GY"
 envs<-c("AN.IN") # Environments
 Comb<-expand.grid(envs,Method,traits)
 p=0.25 # Percentage of predicted individuals
 nRep<-1 #Sampling number (number of TRN-TST sets) - will reflect the seed

 name<-paste(traits,envs,sep="_")
 dir.create(name);setwd(name) # Creating directory to save the outputs
 
 ydat<-subset(y,ENV==envs & Trait==traits)  
 ydat<-scale(ydat$lsmean) #Phenotypes for the analyses
 
 PCa<-PCa[match(rownames(ydat),rownames(WW)),]
 PCd<-PCd[match(rownames(ydat),rownames(WW)),]
 PCaa<-PCaa[match(rownames(ydat),rownames(WW)),]
 PCad<-PCad[match(rownames(ydat),rownames(WW)),]
```

### Variance Components and Covariance Between Effects Estimation

In this section we show how to estimate variance components accounting for the covariance among genetic effects (more detalis in Lehermeier el al.[32]) and how to estimate the covariances between genetic effects.

## Fitting models

```
   ETAad=list(add=list(X=PCa,model=Method,saveEffects=T))
   
   ETAcomp=list(add=list(X=PCa,model=Method,saveEffects=T),
            dom=list(X=PCd,model=Method,saveEffects=T),
            aa=list(X=PCaa,model=Method,saveEffects=T),
            ad=list(X=PCad,model=Method,saveEffects=T))
   
   fmad=BGLR(y=ydat,ETA=ETAad,nIter=nIter,burnIn=burnin,verbose=F) #Fitting the additive model
   
   fmcomp=BGLR(y=ydat,ETA=ETAcomp,nIter=nIter,burnIn=burnin,verbose=F) #Fitting the multi-kernel model
```

## Estimating the genetic variance components and heritabilities

The following code retrieves the samples of effects saved by BGLR and computes, for each of the samples saved, variance components and hertiability. For a simplified example of how to compute variance components that account for LD follow this link. More details of the method is presented in Lehermeier et al. (2017).

```
# Estimating the genetic parameters from the multi-kernel model

   ## Estimating the genetic effects for each sample (Add, Dom, AdAd,AdDom) 
  
   Ba<-readBinMat('ETA_add_b.bin')
   Ad<-apply(Ba,1,function(x){ua<-PCa%*%x; return(UA=ua)}) #Additive effects
  
   Baa<-readBinMat('ETA_aa_b.bin')
   AdAd<-apply(Baa,1,function(x){ua<-PCaa%*%x;return(UA=ua)})#Additive by additive epistasis effects
  
   Bd<-readBinMat('ETA_dom_b.bin')
   Dom<-apply(Bd,1,function(x){ ud<-PCd%*%x; return(UD=ud) })#Dominance effects
    
   Bad<-readBinMat('ETA_ad_b.bin')
   AdDom<-apply(Bad,1,function(x){ud<-PCad%*%x;return(UD=ud)})#Additive by dominance epistasis effects
    
    ## Estimating Variances parameters for each Gibbs Sampler
    
    var_D<-apply(Dom,2,var) # Dominance variance
    var_A<-apply(Ad,2,var) # Additive variance
    var_AA<-apply(AdAd,2,var) # Additive by additive epistasis variance
    var_AD<-apply(AdDom,2,var) #Additive by dominance epistasis variance
    
    ## Total genomic variance explained by the model
    
    u=Ad+Dom+AdAd+AdDom #Total genetic value of hybrids for each sample
    var_U=apply(u,2,var)#Total genetic variance explained by the model for each sample
    
    ## Estimating the error variance  
    YY<-matrix(rep(ydat,times=(nIter-burnin)/thin),ncol=(nIter-burnin)/thin,byrow=F)
    Error<-apply(YY-u,2,function(x){
                          Mat<-matrix(x,ncol=1,byrow=F);
                          varErr<-apply(Mat,2,var,na.rm=T);
                          return(varErr)}
                 )
    
    # Broad-sense genomic heritability
    H2<-var_U/(var_U+Error)   
  
# Estimating the General and Specific Combining Abilities variances (GCA and SCA, respectively) 
  
   ## Estimating the GCA (total genetic variance explained by the additive model)
   
   BGCA<-readBinMat('Add_ETA_add_b.bin')
   GCA<-apply(Ba,1,function(x){ua<-PCa%*%x; return(UA=ua)}) #Additive effects
   var_GCA<-apply(GCA,2,var)
  
   ## Estimating the SCA (total genetic variance explained by multi-kernel model (var_U) minus the var_GCA)  
   
   var_SCA<-var_U-var_GCA

# Estimating the posterior means and standard deviations of the genetic variances components and parameters
   
   Components<-cbind(var_GCA,var_SCA,var_U,var_A,var_D,var_AA,var_AD,Error,H2)   
   MeanVarComp<-colMeans(Components)
   SDVarComp<-apply(Components,2,sd)
```

## Estimating the covariance between the genetic effects

The following code retrieves the samples of effects saved by BGLR and computes, for each of the samples saved, the covariances between the modeled genetic effects. To estimate the covariances one must use the genetic effects (`Ad`,`Dom`,`AdAd`, and `AdDom`) and the genetic variances (`var_A`,`var_D`,`var_AA`, and `var_AD`) estimated in the last section. The covariances were estimated assuming:\(COV(a,b)=\frac{Var(a+b)-Var(a)-Var(b)}{2}\)

```
    # Pairwise combination of all genetic effects   
    
    uAD=Ad+Dom
    uAAA<-Ad+AdAd
    uAAD<-Ad+AdDom
    uDAA<-Dom+AdAd
    uDAD<-Dom+AdDom
    
    # Variance associated to each pair of genetic effects
    
    var_UAD=apply(uAD,2,var)
    var_UAAA=apply(uAAA,2,var)
    var_UAAD=apply(uAAD,2,var)
    var_DAA=apply(uDAA,2,var)
    var_DAD=apply(uDAD,2,var)

    
    # Covariance between effects 
    
    COVAd_Dom<-(var_UAD-var_D-var_A)/2
    COVAd_AdAd<-(var_UAAA-var_AA-var_A)/2
    COVAd_AdDom<-(var_UAAD-var_AD-var_A)/2
    COVDom_AdAd<-(var_DAA-var_AA-var_D)/2
    COVDom_AdDom<-(var_DAD-var_AD-var_D)/2
    
    # Generating the covariance matrix (ncol=5, nrow= number of samples)
    
    Covs<-cbind(COVAd_Dom,COVAd_AdAd,COVAd_AdDom,COVDom_AdAd,COVDom_AdDom)
```

### Evaluation of prediction accuracy in training-testing partitions

```
  COR<-NULL #Prediction accuracy
  
  for (j in 1:nRep){
    set.seed(j)
      
  # Sampling the training and testing set
    
      NAs<-sample(1:nrow(ydat),size=p*nrow(ydat))
      yNAs<-ydat
      yNAs[NAs,]<-NA
      yNAs<-as.vector(yNAs)
      save(NAs,file=paste("NAs",traits,"_",envs,"_",j,".RData",sep=""))

  ## Fitting the model
  
    ETA=list(add=list(X=PCa,model=Method,saveEffects=F),
           dom=list(X=PCd,model=Method,saveEffects=F),
           aa=list(X=PCaa,model=Method,saveEffects=F),
           ad=list(X=PCad,model=Method,saveEffects=F))

  fm=BGLR(y=yNAs,ETA=ETA,nIter=nIter,burnIn=burnin,verbose=F)
  save(fm,file=paste(traits,"_",envs,"_",j,".RData",sep=""))

  ### Prediction Accuracy
        
        yHat<-fm$yHat[NAs]
        tst<-ydat[NAs]
        COR[j]<-cor(yHat,tst,use="complete.obs")
        
    cat("REP=",j,"\n")
  }
```

### Accuracy of pre-screening

Here we estimate the proportion of the top-5% hybrids (according to phenotypic rank) that is captured by pre-screening based on (cross-validation) genomic prediction at a different intensity of selection (PERC)

To run the scrip below one must use as working directory the directory containing the saved testing set and its predicted values for each cross-validation.

```
# Determining a intensity of selection sequency using genomic selection (q1)
Combna<-expand.grid(q1=seq(0.05,0.3,0.01),q2=0.05)
PERC<-list() # List containing proportion from the gradient of q1 
for(i in 1:nrow(Combna)){
  q1<-Combna[i,1] #Percentage selected by Genomic Selection
  q2=Combna[i,2] #Percentage selected by phenotypic selection
  Perc=NULL
  for (j in 1:length(which(grepl("NAs",dir())==T))){
    # Estimate the proportion of of coincidence by cross-validation (i.e. 100 sets) 
    load(dir()[grepl(paste(traits,"_",envs,"_",j,".RData",sep=""),dir())][1])
    load(dir()[grepl(paste("NAs",traits,"_",envs,"_",j,".RData",sep=""),dir())])
    yHat<-fm$yHat
    yHat[which(is.na(ydat))]<-NA
    isTopPred=na.omit(yHat[NAs])>quantile(na.omit(yHat[NAs]),p=1-q1,na.rm=T)
    isTopObserved=na.omit(ydat[NAs])>quantile(na.omit(ydat[NAs]),p=1-q2,na.rm=T)
    Perc[j]<-sum(isTopObserved[isTopPred]) /sum(isTopObserved) ## Coincidence
  }
  
  PERC[[i]]<-Perc  
}

PERC<-do.call(rbind,lapply(1:length(PERC),function(x) {meanss<-mean(PERC[[x]])
return(meanss)})) 
rownames(PERC)<-Combna[,1]
```
