# Supplementary figures and images for: Bayesian analysis and prediction of hybrid performance
Source: Plant Methods. 2019 Feb 7;15:14. doi: 10.1186/s13007-019-0388-x (PMC6366084; doi:10.1186/s13007-019-0388-x)

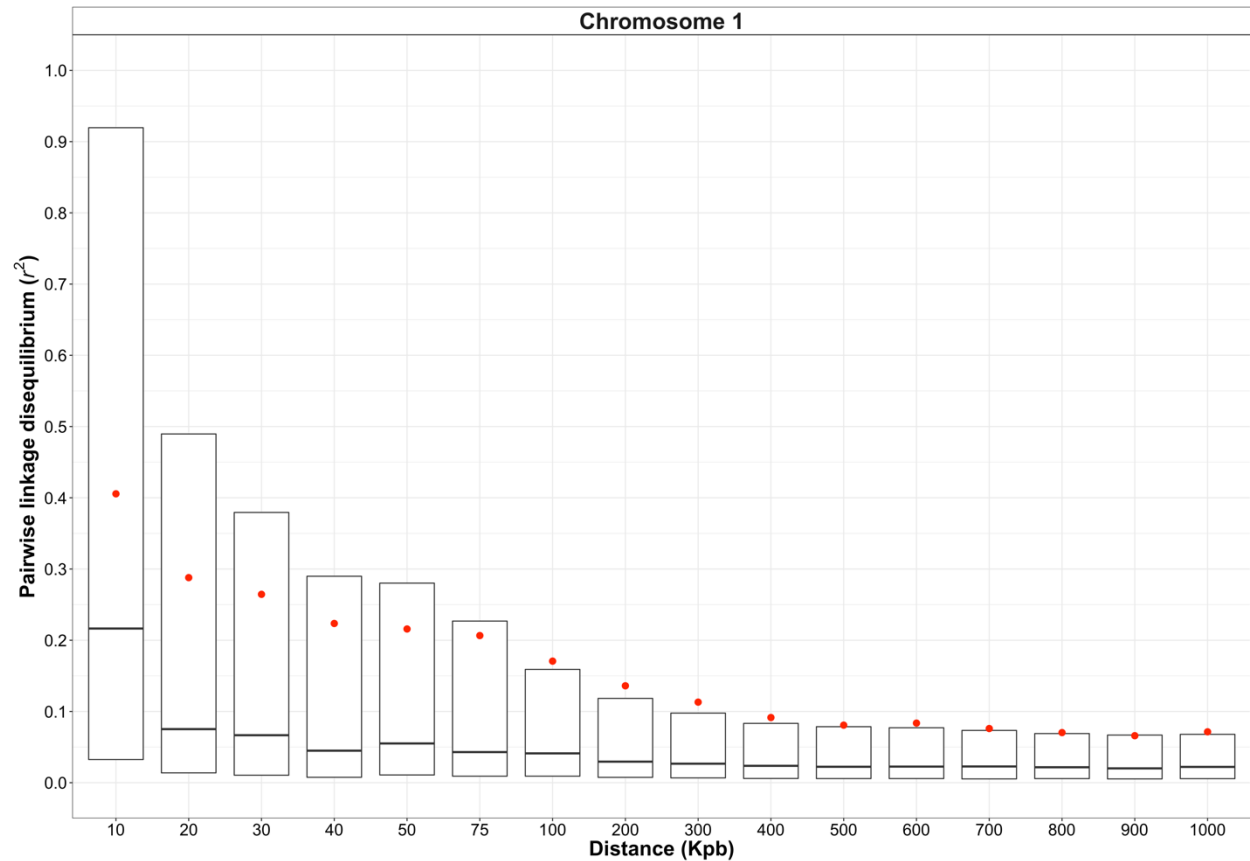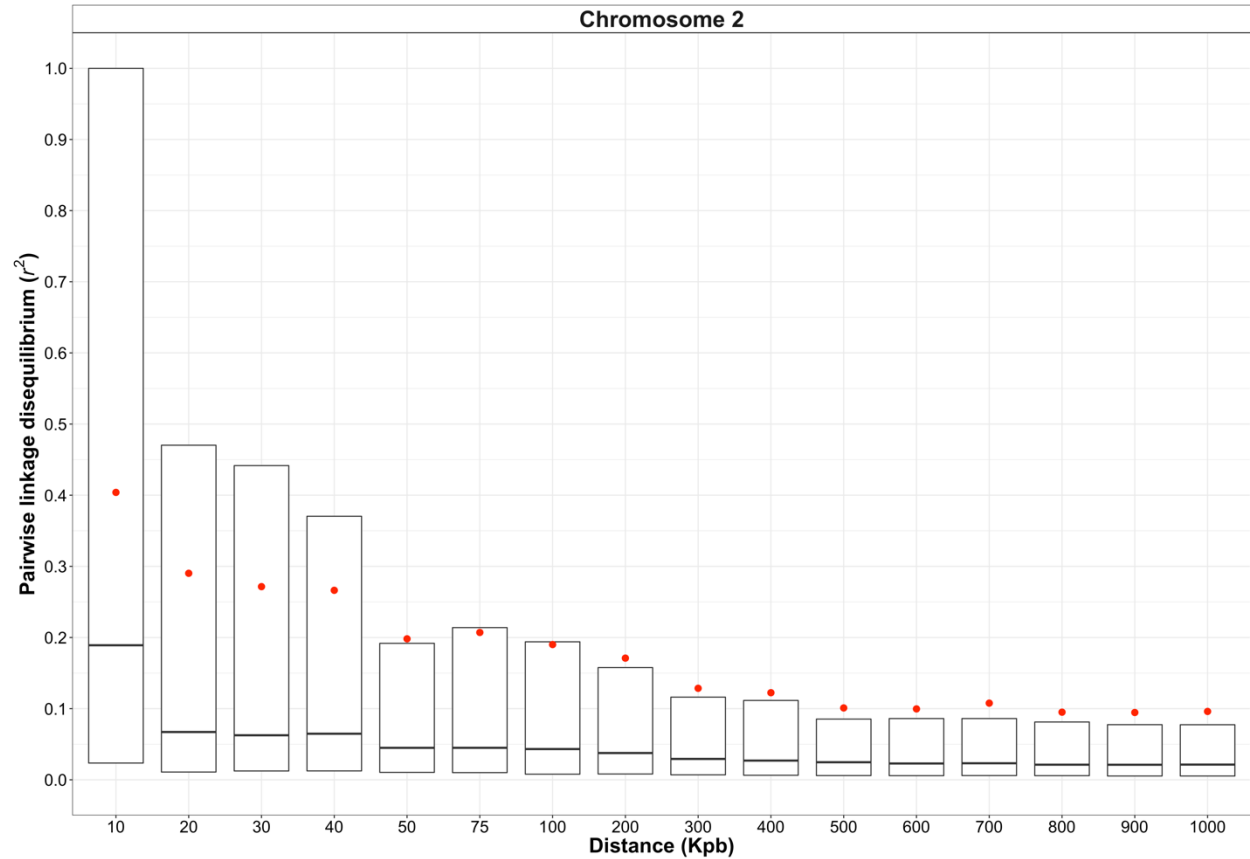

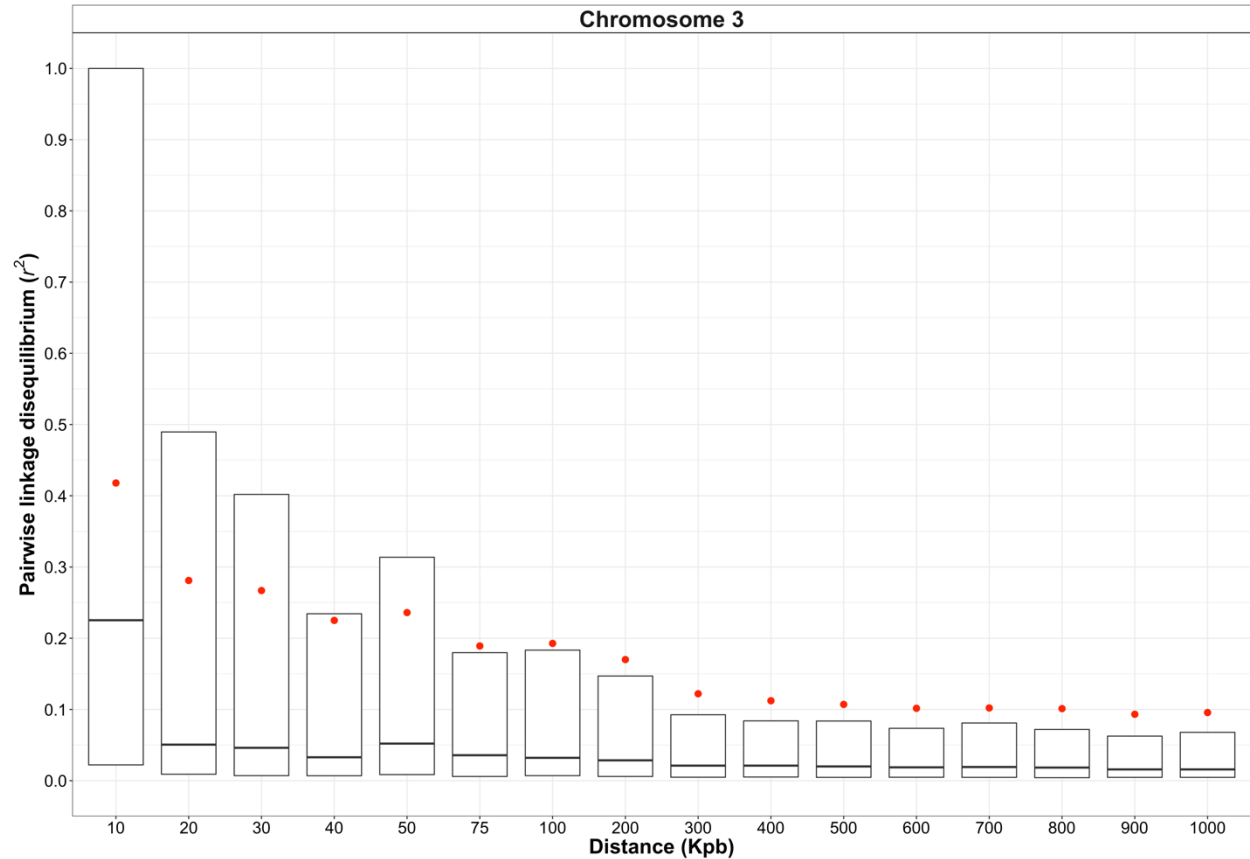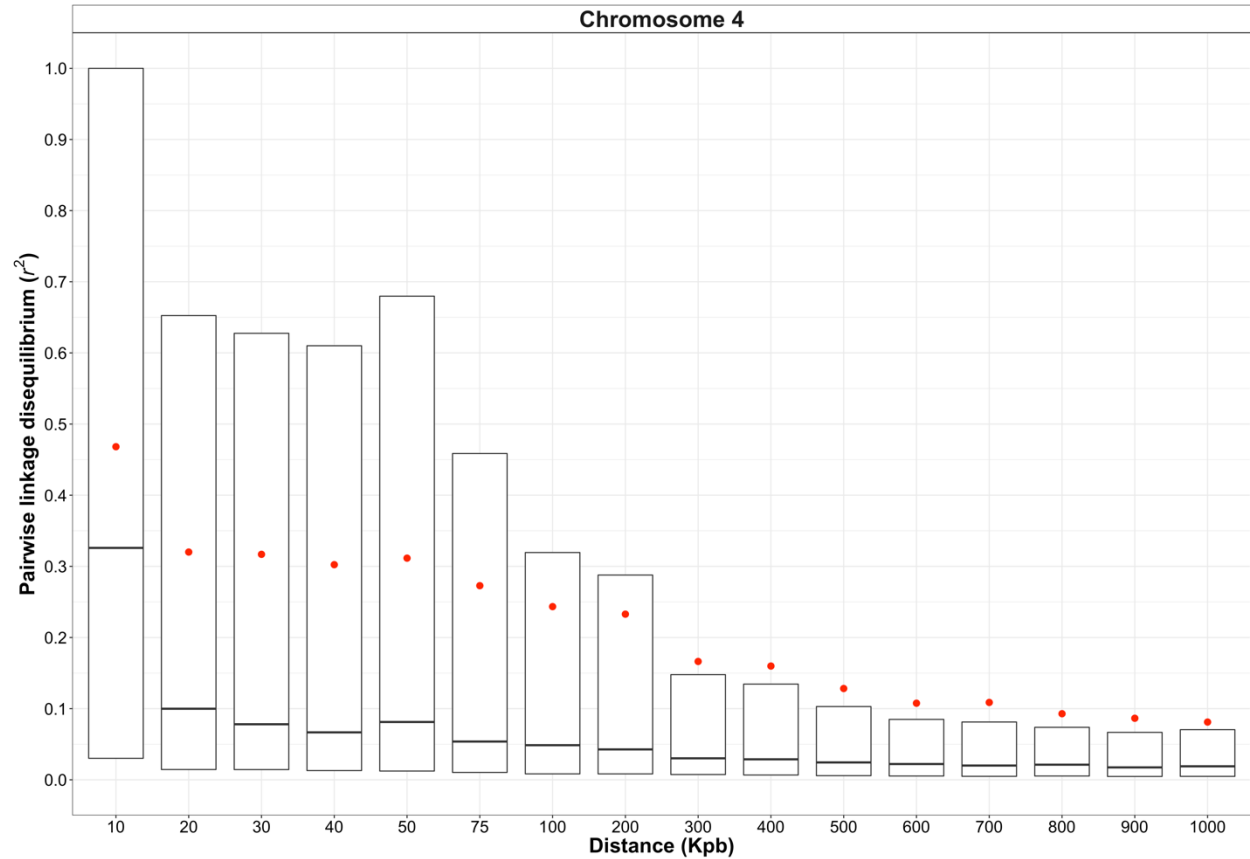

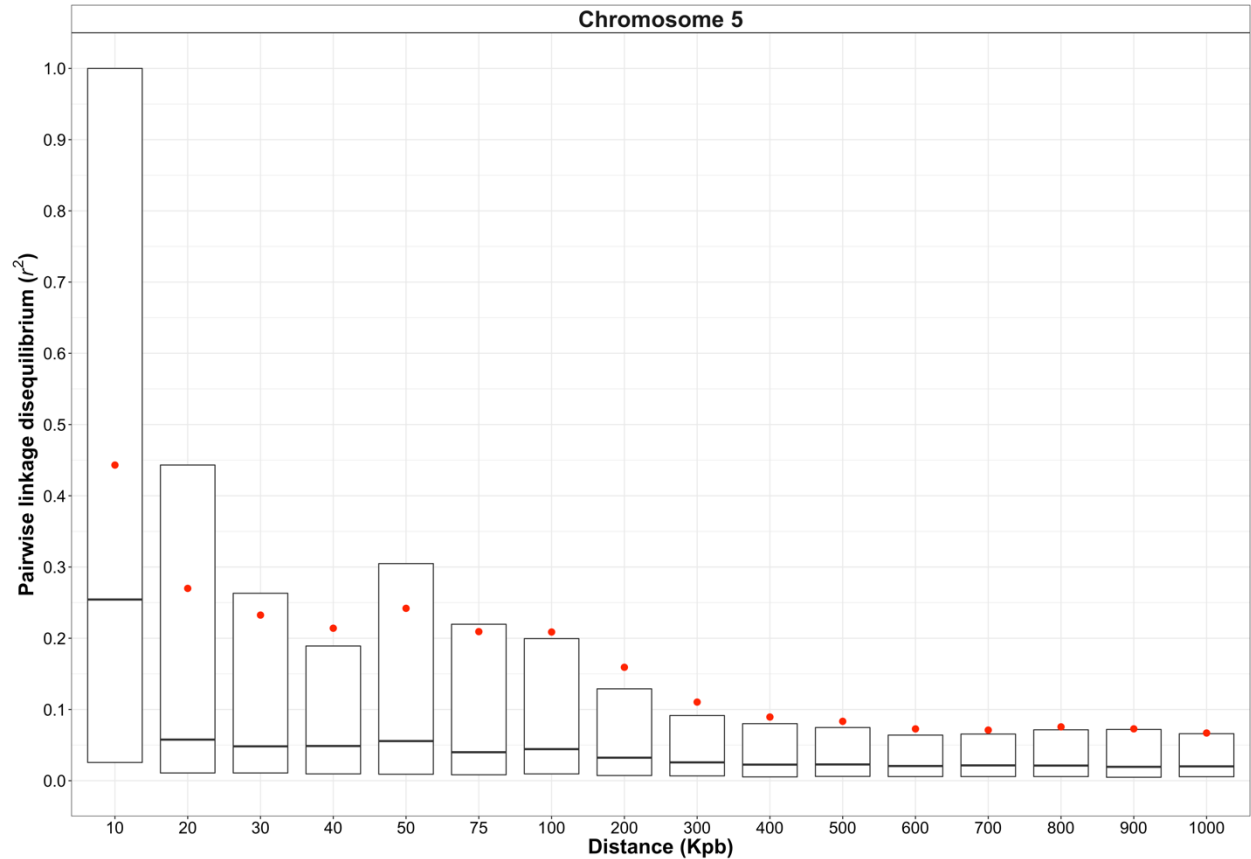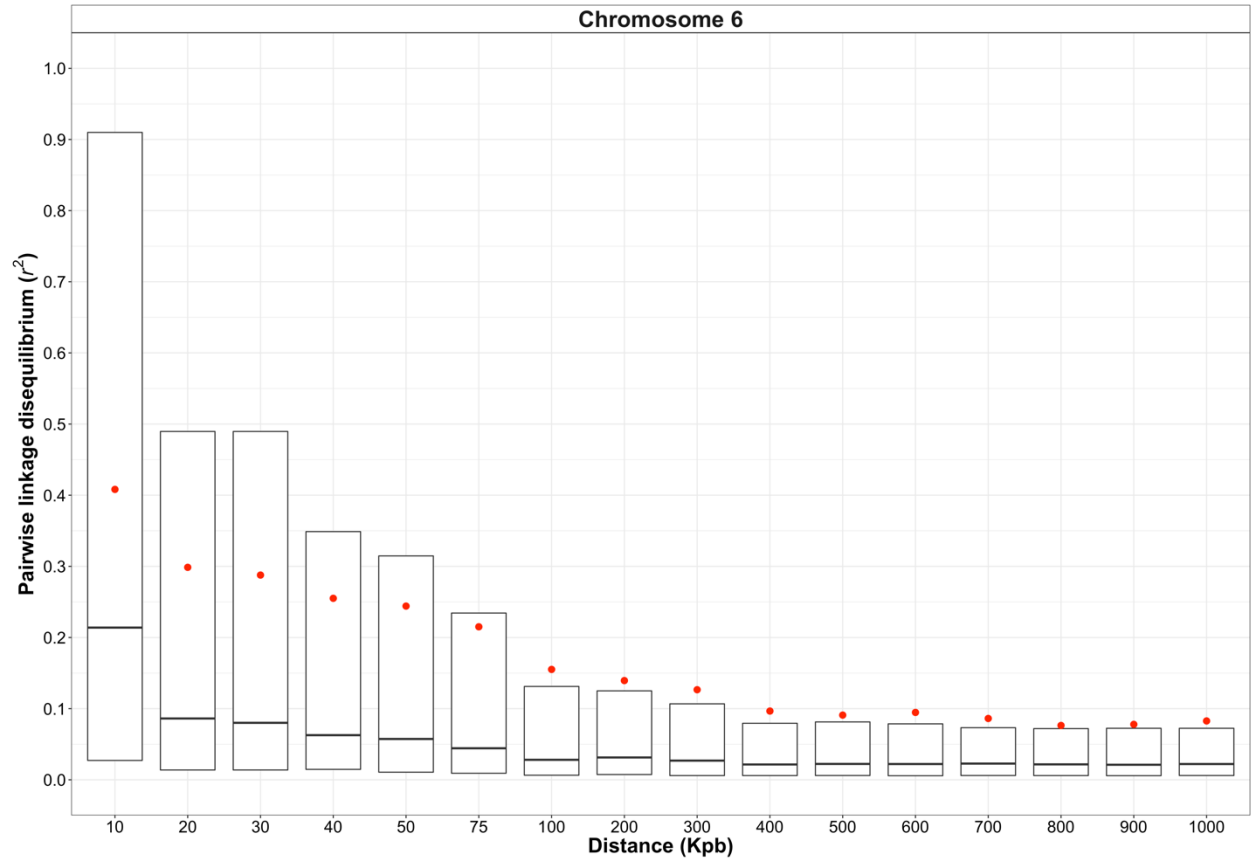

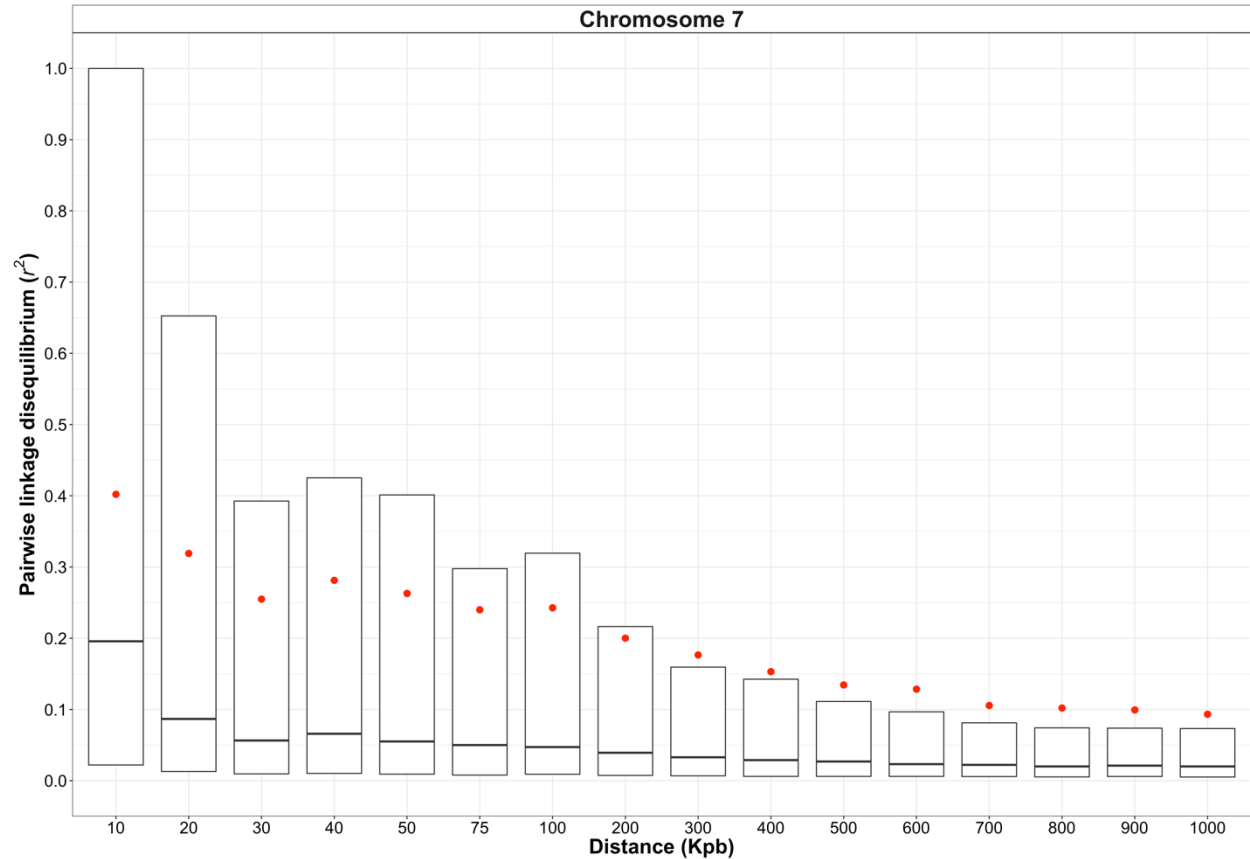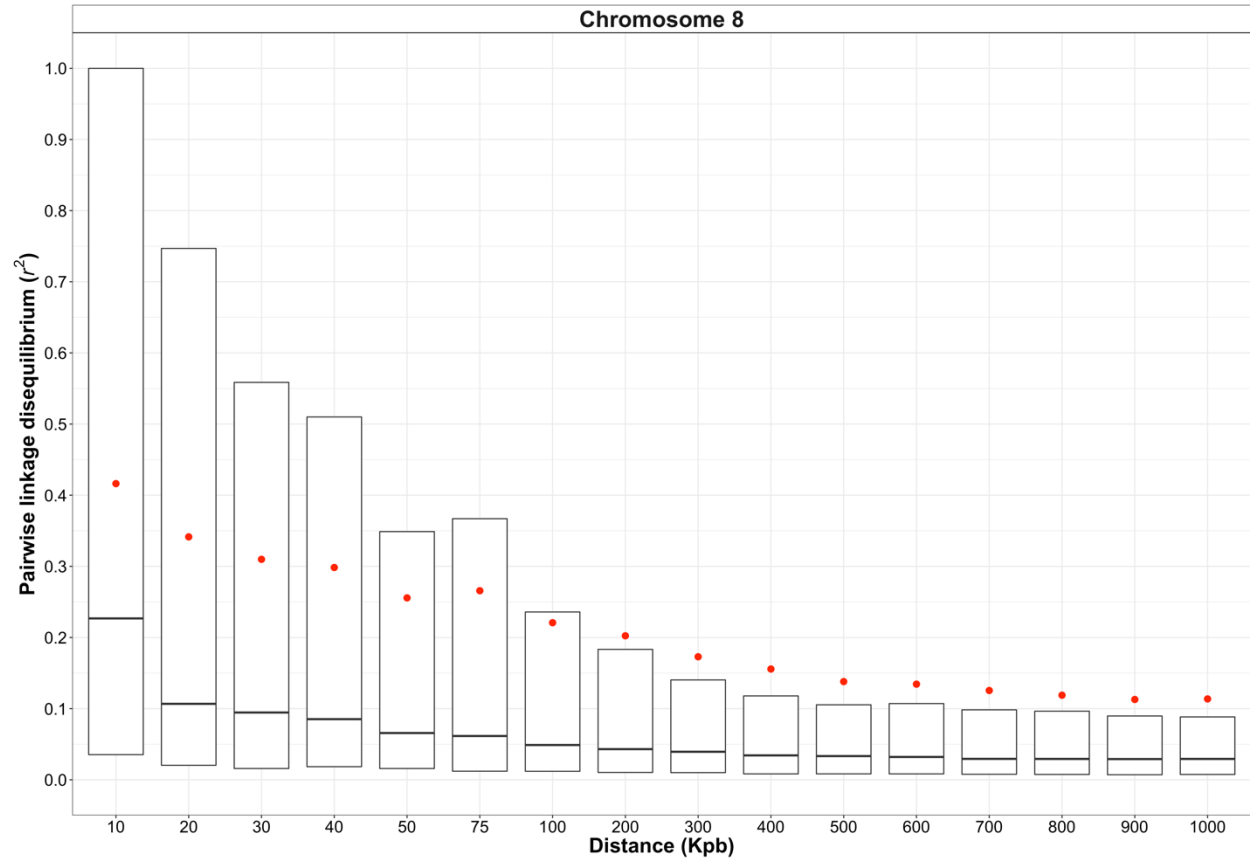

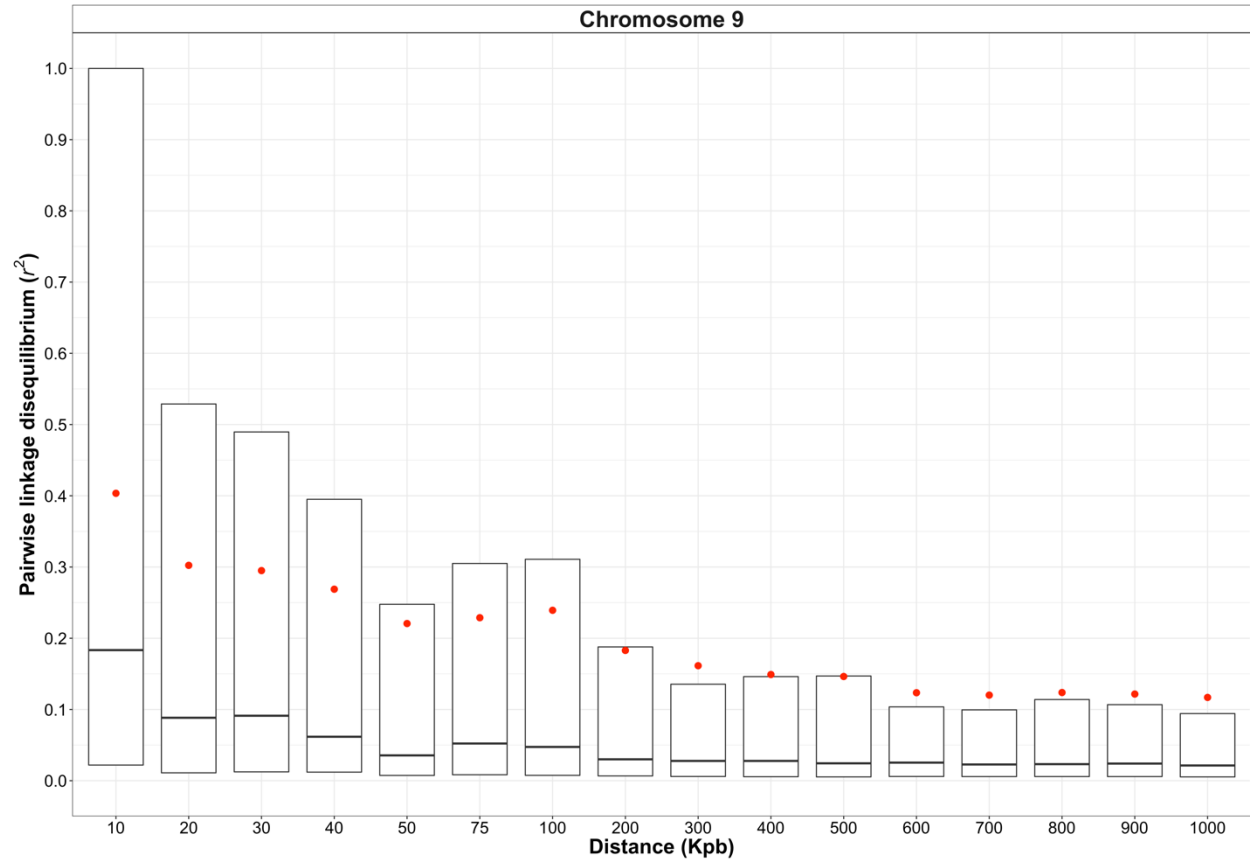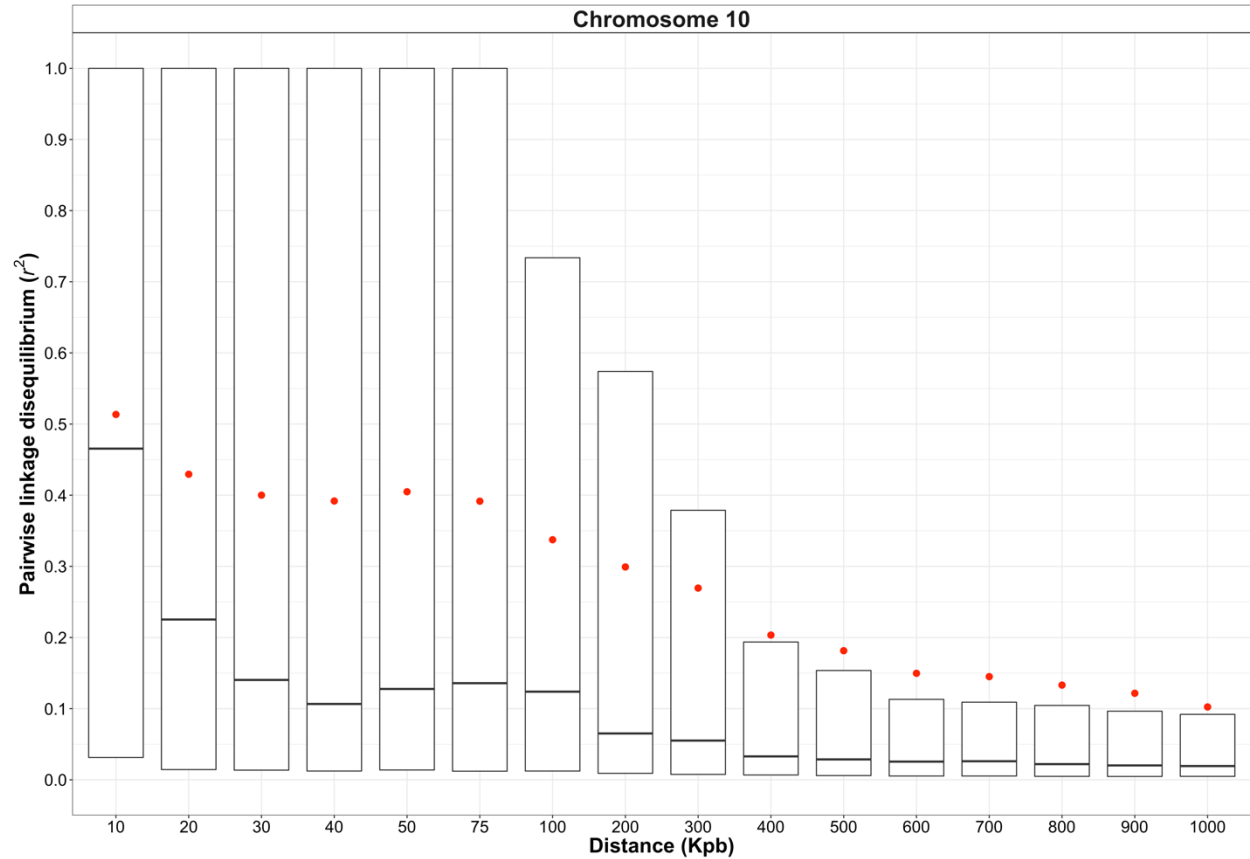

Supplement: Supplementary file 4 — Additional file 4. Fig. S2: Boxplots of the pairwise linkage disequilibrium (r2) by distance (Kbp) for chromosomes 1 to 10 in 49 inbred lines of tropical maize. Red dots and black traces represent means and medians, respectively. Each box represents the r2 estimates inside the first and third quartiles (25 and 75% percentiles, respectively). [file 13007_2019_388_MOESM4_ESM.pdf]

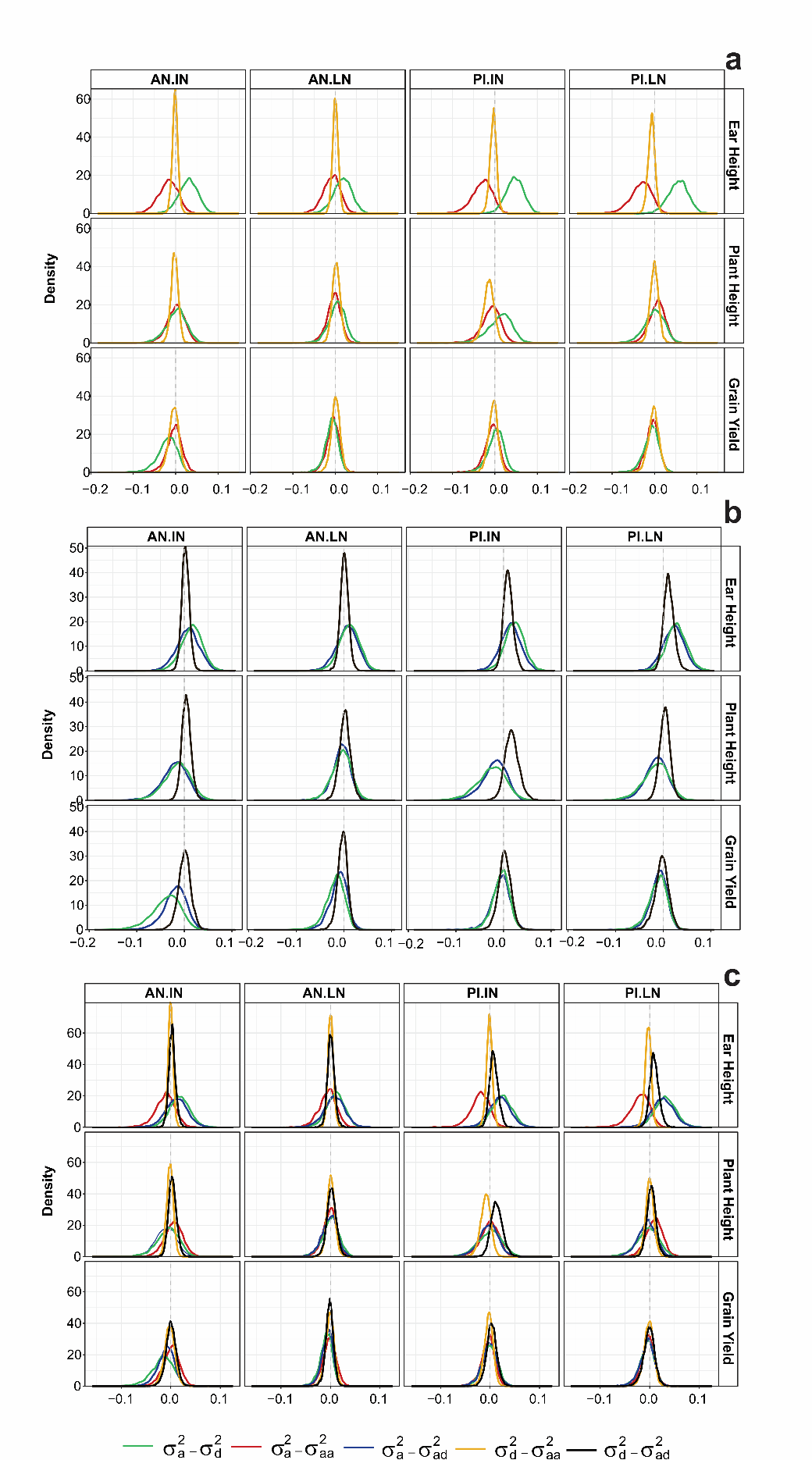

Supplement: Supplementary file 6 — Additional file 6. Fig. S3: Posterior density of the covariance between the additive and nonadditive genetic components by environment and traits. a A + D+AA model. b A + D+AD model. c A + D+AA + AD model. Covariance between A and D effects is represented in green ($\upsigma_{\text{a}}^{2}$σa2–$\upsigma_{\text{d}}^{2}$σd2); Covariance between A and AA is represented in red ($\upsigma_{\text{a}}^{2}$σa2–$\upsigma_{\text{aa}}^{2}$σaa2); Covariance between A and AD is represented in blue ($\upsigma_{\text{a}}^{2}$σa2–$\upsigma_{\text{ad}}^{2}$ [file 13007_2019_388_MOESM6_ESM.docx]

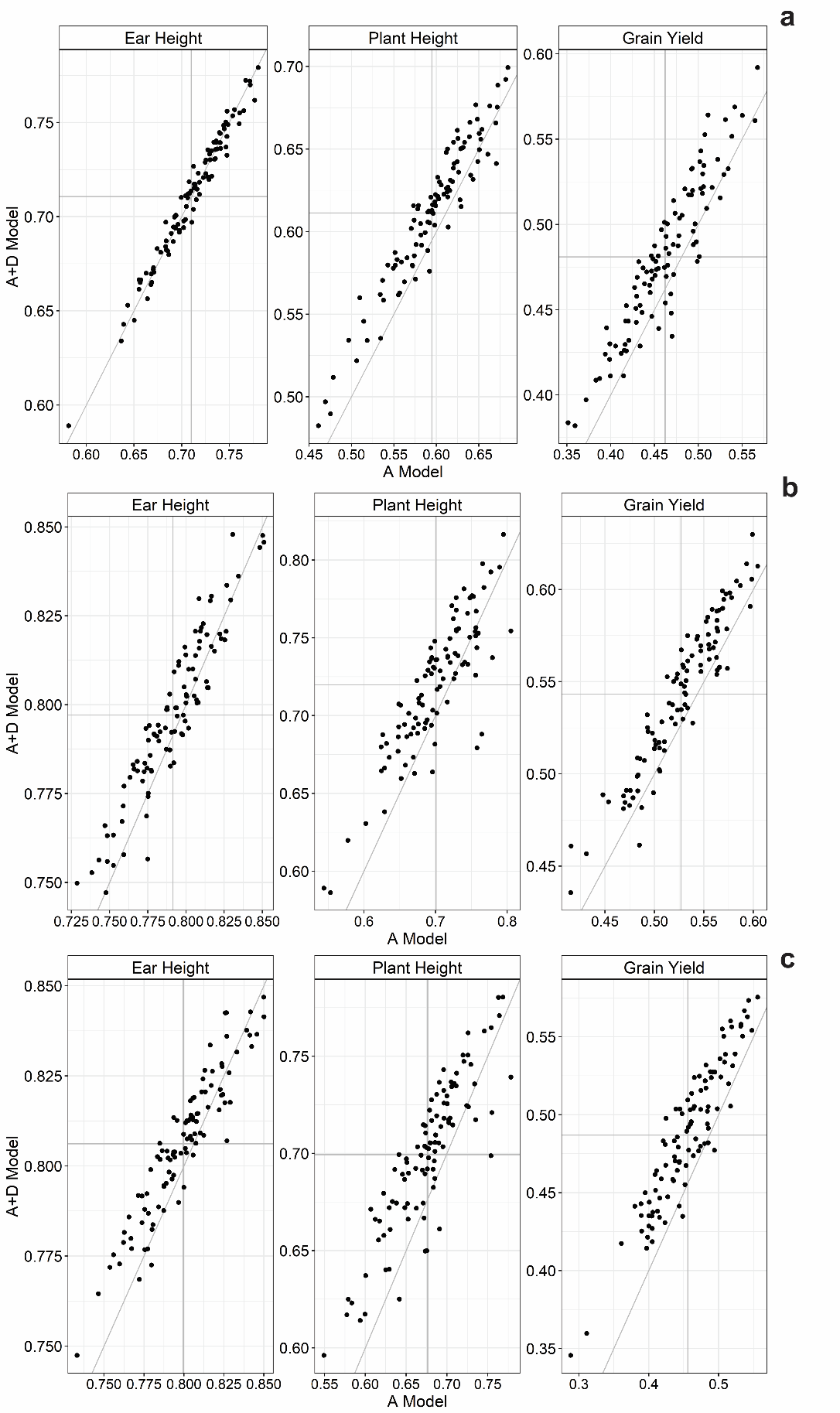

Supplement: Supplementary file 8 — Additional file 8. Fig. S4: Scatter plots of the prediction accuracies obtained by the A + D model (Additive-dominance) and A (Additive) model by trait. a: AN.LN: Anhembi low nitrogen availability; b: PI.IN: Piracicaba ideal nitrogen availability; c: PI.LN: Piracicaba low nitrogen availability. Each point represents one TRN-TST partition. The same population partitions across models were considered. [file 13007_2019_388_MOESM8_ESM.docx]

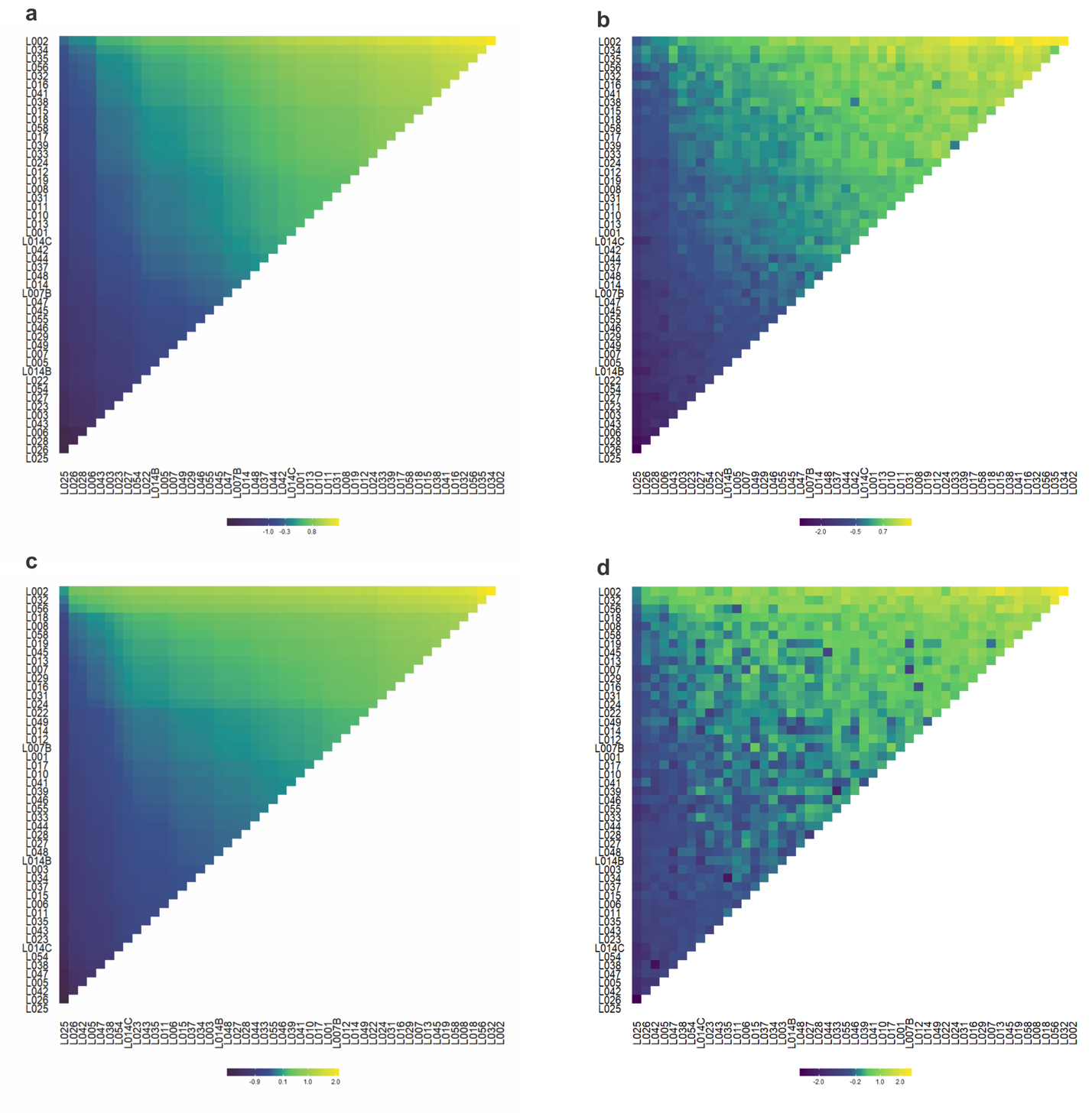

Supplement: Supplementary file 9 — Additional file 9. Fig. S5: Heatmaps of predicted values of all possible single-crosses hybrids at Piracicaba with low nitrogen availability (AN.LN). a, b Ear height predicted using the additive and additive-dominance models; c, d Grain yield predicted using the additive and additive-dominance models. Lines and columns of each plot were sorted by the mean performance of parental inbred lines at all crosses considering the predicted values predicted in the Additive model. [file 13007_2019_388_MOESM9_ESM.docx]

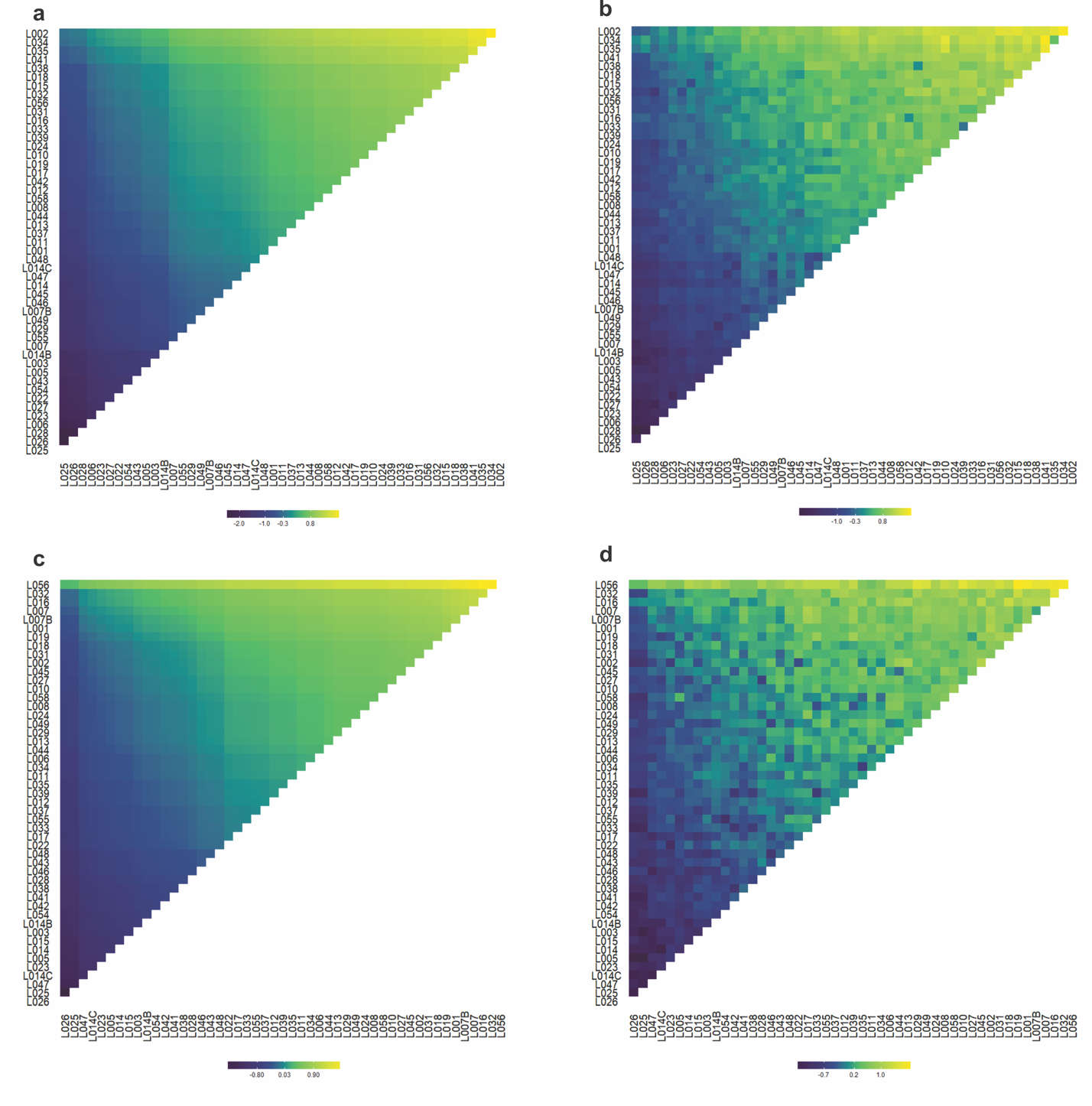

Supplement: Supplementary file 10 — Additional file 10. Fig. S6: Heatmaps of predicted values of all possible single-crosses hybrids at Piracicaba with ideal nitrogen availability (PI.IN). a, b Ear height predicted using the additive and additive-dominance models; c, d Grain yield predicted using the additive and additive-dominance models. Lines and columns of each plot were sorted by the mean performance of parental inbred lines at all crosses considering the predicted values predicted in the Additive model. [file 13007_2019_388_MOESM10_ESM.docx]

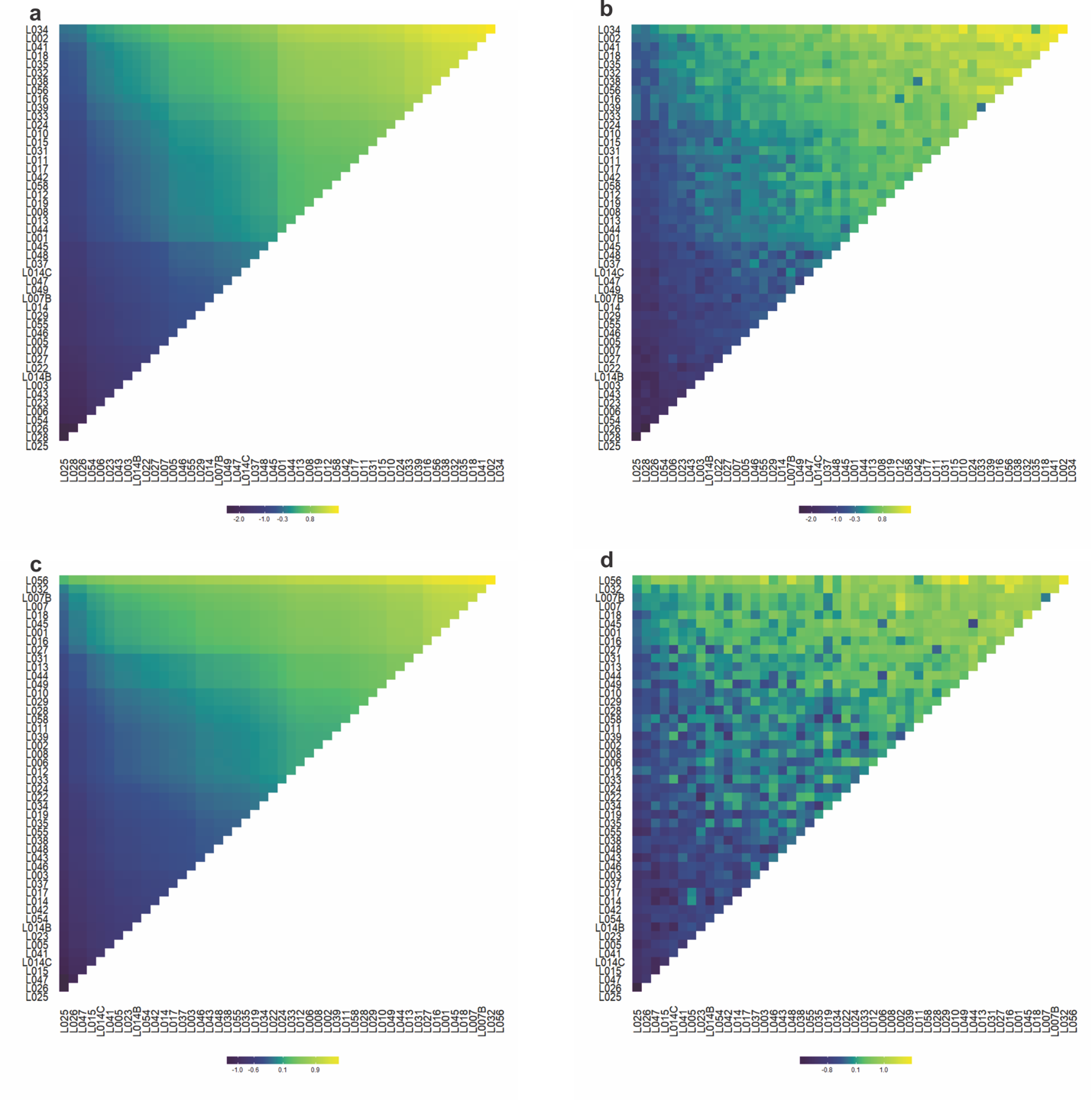

Supplement: Supplementary file 11 — Additional file 11. Fig. S7: Heatmaps of predicted values of all possible single-crosses hybrids at Piracicaba with low nitrogen availability PI.LN. a, b Ear height predicted using the additive and additive-dominance models; c, d Grain yield predicted using the additive and additive-dominance models. Lines and columns of each plot were sorted by the mean performance of parental inbred lines at all crosses considering the predicted values predicted in the Additive model. [file 13007_2019_388_MOESM11_ESM.docx]

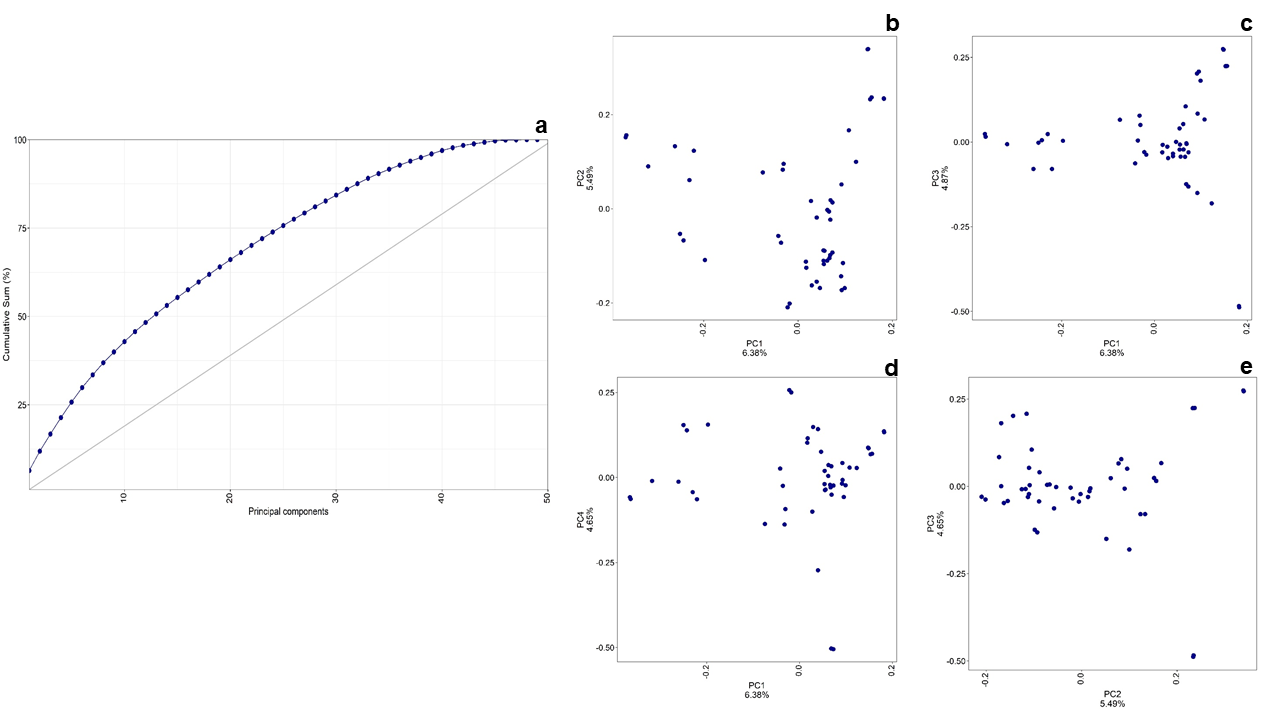

Supplement: Supplementary file 12 — Additional file 12. Fig. S8: Population structure of 49 inbred lines in maize. a Cumulative proportion of variance explained by the principal components (PC). b PC2 versus PC1. c PC3 versus PC1. d PC4 versus PC1. e PC3 versus PC2. [file 13007_2019_388_MOESM12_ESM.docx]

**
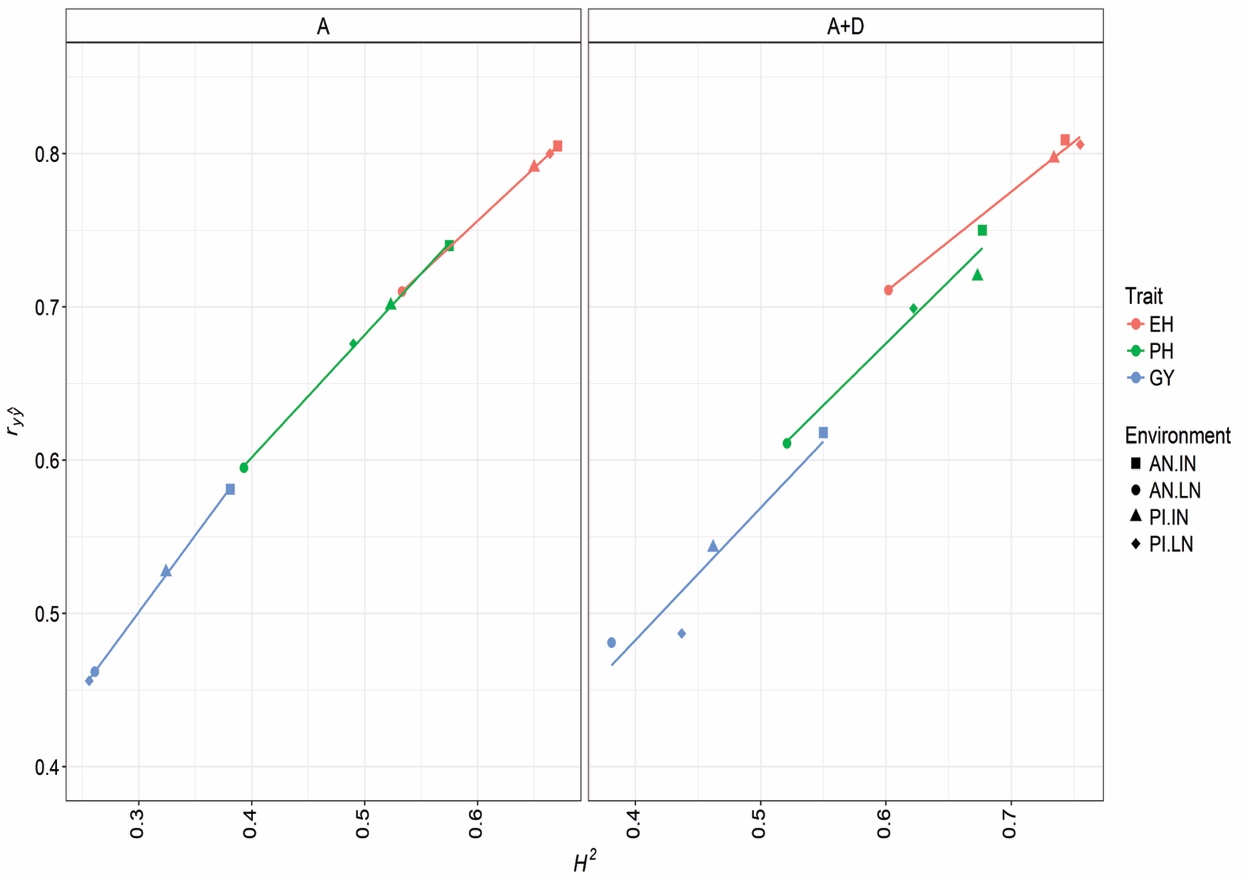
**

Supplement: Supplementary file 13 — Additional file 13. Fig. S9: Linearity among prediction accuracy ($r_{{y\hat{y}}}$ryy^) and broad-sense genomic heritability ($H^{2}$H2) by trait/environment within prediction model. EH: Ear height, PH: Plant height, and GY: Grain yield. AN: Anhembi, PI: Piracicaba, IN: Ideal nitrogen, and LN: Low nitrogen. A: Additive effect, and D: Dominance effects [file 13007_2019_388_MOESM13_ESM.docx]

**
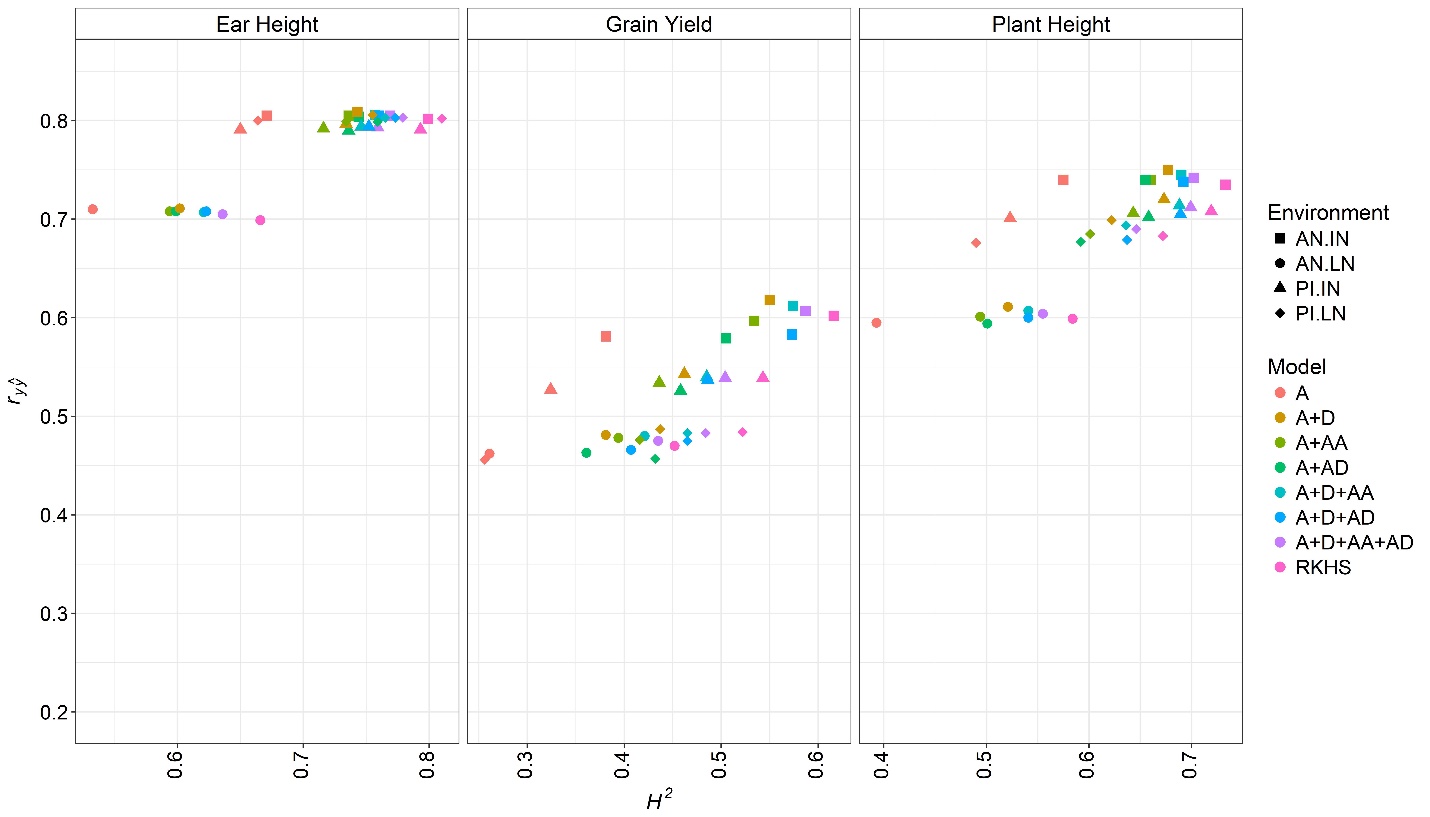
**

Supplement: Supplementary file 14 — Additional file 14. Fig. S10: Cross-validation prediction accuracy ($r_{yy}^{{}}$ryy) versus broad-sense genomic heritability ($H^{2}$H2) by trait, environment, and model. A: Additive, D: Dominance, AA: Additive x additive, and AD: Additive x dominance effects. RKHS: Reproducing Kernel Hilbert Spaces model). AN: Anhembi, PI: Piracicaba, LN: Low nitrogen, IN: Ideal nitrogen. [file 13007_2019_388_MOESM14_ESM.docx]
