# Supplementary material for: Bayesian analysis and prediction of hybrid performance
Source: Plant Methods. 2019 Feb 7;15:14. doi: 10.1186/s13007-019-0388-x (PMC6366084; doi:10.1186/s13007-019-0388-x)
Supplement: Supplementary file 4 [file 13007_2019_388_MOESM16_ESM.docx]

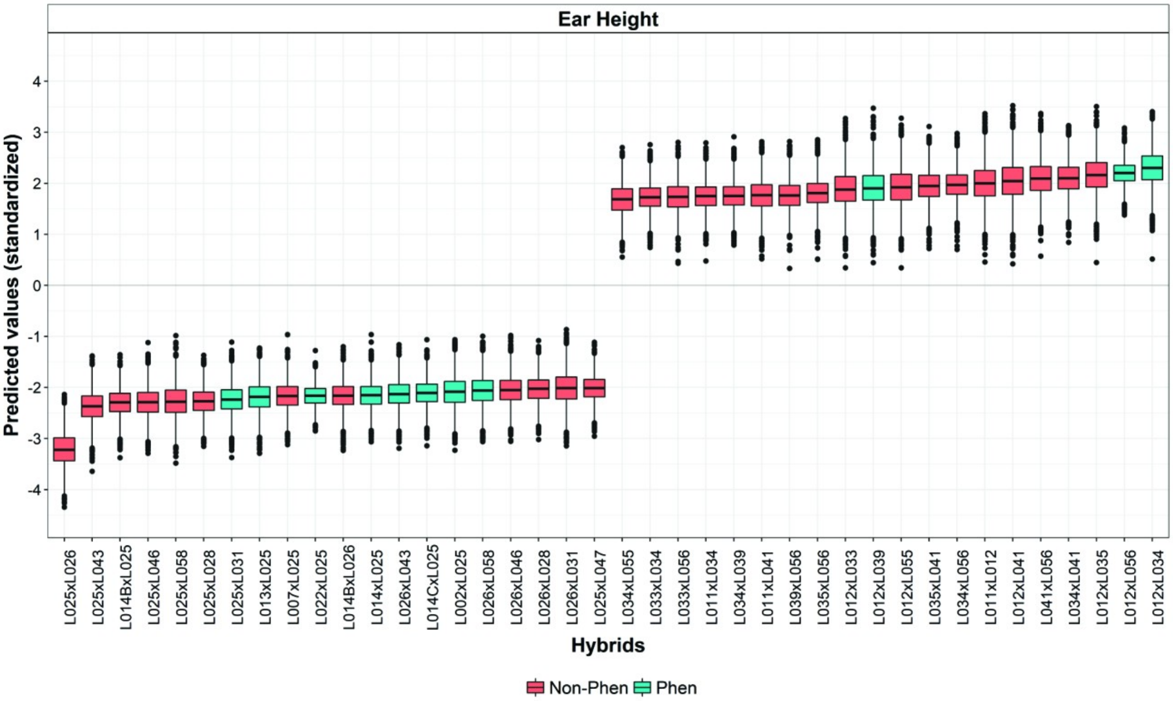
Ear Height
Predicted values (standardized)
4
3
2
1
0
-1
-2
-3
-4
L025xL026
L025xL043
L014BxL025
L025xL046
L025xL058
L025xL028
L025xL031
L013xL025
L007xL025
L022xL025
L014BxL026
L014xL025
L026xL043
L014CxL025
L002xL025
L026xL058
L026xL046
L026xL028
L026xL031
L025xL047
L034xL055
L033xL034
L033xL056
L011xL034
L034xL039
L011xL041
L039xL056
L035xL056
L012xL033
L012xL039
L012xL055
L035xL041
L034xL056
L011xL012
L012xL041
L041xL056
L034xL041
L012xL035
L012xL056
L012xL034
Hybrids
Non-Phen
Phen

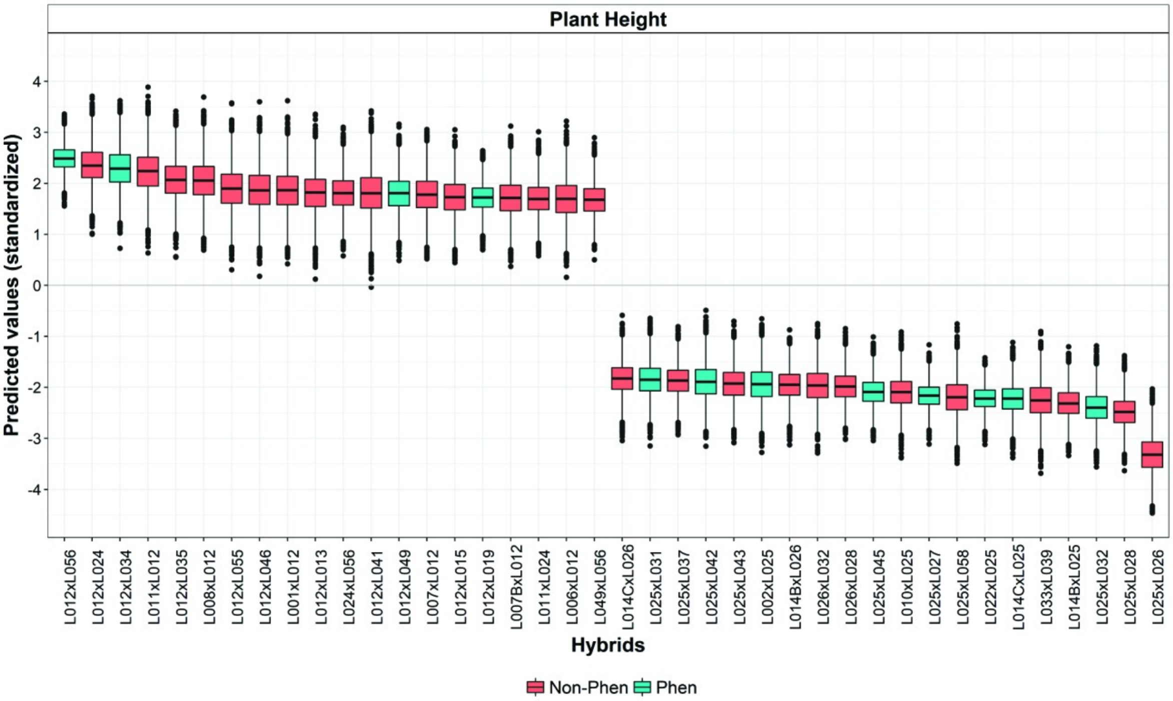
Plant Height
Predicted values (standardized)
4
3
2
1
0
-1
-2
-3
-4
L012xL056
L012xL024
L012xL034
L011xL012
L012xL035
L008xL012
L012xL055
L012xL046
L001xL012
L012xL013
L024xL056
L012xL041
L012xL049
L007xL012
L012xL015
L012xL019
L007BxL012
L011xL024
L006xL012
L049xL056
L014CxL026
L025xL031
L025xL037
L025xL042
L025xL043
L002xL025
L014BxL026
L026xL032
L026xL028
L025xL045
L010xL025
L025xL027
L025xL058
L022xL025
L014CxL025
L033xL039
L014BxL025
L025xL032
L025xL028
L025xL026
Hybrids
Non-Phen
Phen
