# Supplementary material for: Bayesian analysis and prediction of hybrid performance
Source: Plant Methods. 2019 Feb 7;15:14. doi: 10.1186/s13007-019-0388-x (PMC6366084; doi:10.1186/s13007-019-0388-x)
Supplement: Supplementary file 5 [file 13007_2019_388_MOESM17_ESM.docx]

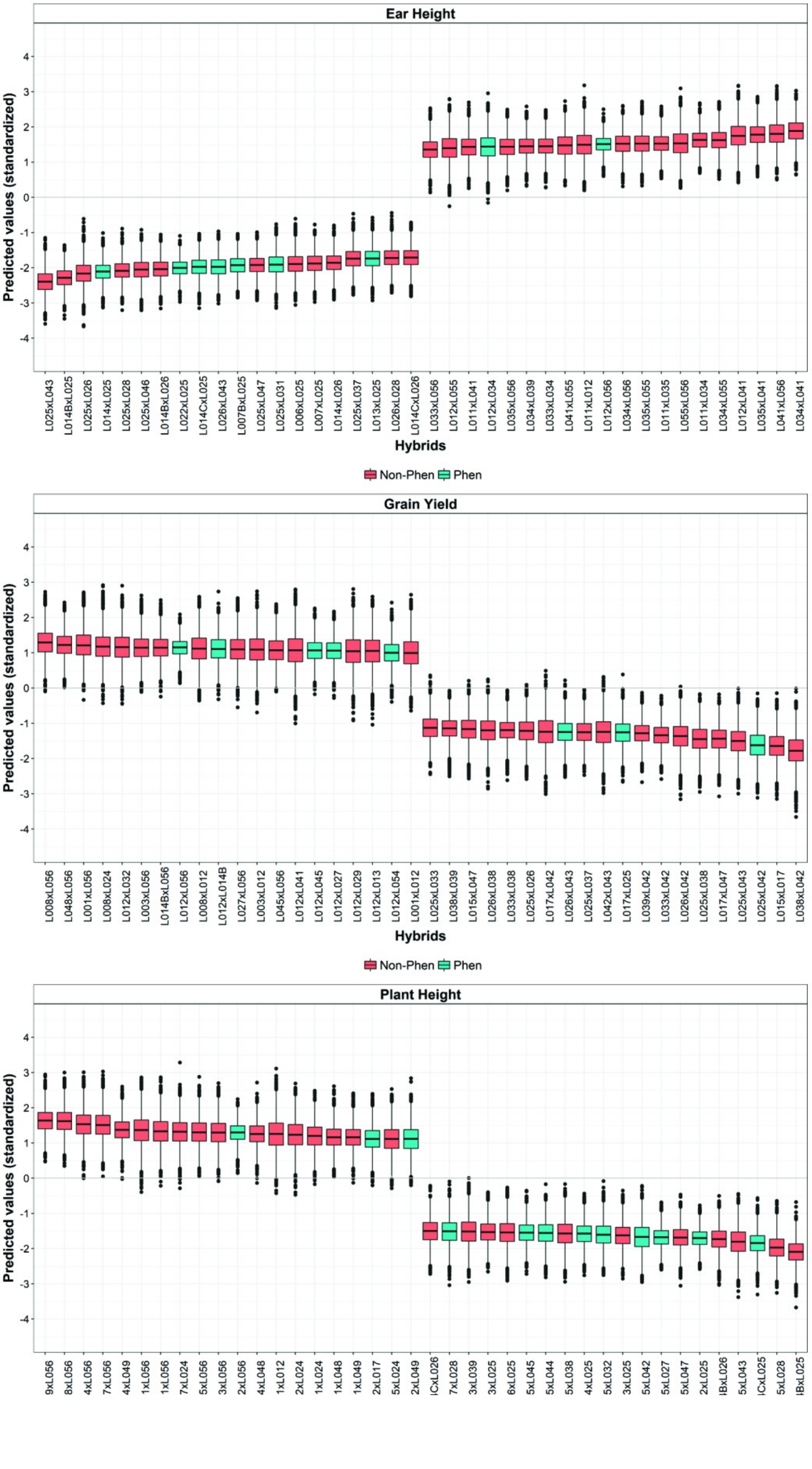

Ear Height
Predicted values (standardized)
4
3
2
1
0
-1
-2
-3
-4
L025xL043
L014BxL025
L025xL026
L014xL025
L025xL028
L025xL046
L014BxL026
L022xL025
L014CxL025
L026xL043
L007BxL025
L025xL047
L025xL031
L006xL025
L007xL025
L014xL026
L025xL037
L013xL025
L026xL028
L014CxL026
L033xL056
L012xL055
L011xL041
L012xL034
L035xL056
L034xL039
L033xL034
L041xL055
L011xL012
L012xL056
L034xL056
L035xL055
L011xL035
L055xL056
L011xL034
L034xL055
L012xL041
L035xL041
L041xL056
L034xL041
Hybrids
Non-Phen
Phen
Grain Yield
Predicted values (standardized)
L008xL056
L048xL056
L001xL056
L008xL024
L012xL032
L003xL056
L014BxL056
L012xL056
L008xL012
L012xL014B
L027xL056
L003xL012
L045xL056
L012xL041
L012xL045
L012xL027
L012xL029
L012xL013
L012xL054
L001xL012
L025xL033
L038xL039
L015xL047
L026xL038
L033xL038
L025xL026
L017xL042
L026xL043
L025xL037
L042xL043
L017xL025
L039xL042
L033xL042
L026xL042
L025xL038
L017xL047
L025xL043
L025xL042
L015xL017
L038xL042
Hybrids
Non-Phen
Phen
Plant Height
Predicted values (standardized)
9xL056
8xL056
4xL056
7xL056
4xL049
1xL056
1xL056
7xL024
5xL056
3xL056
2xL056
4xL048
1xL012
2xL024
1xL024
1xL048
1xL049
2xL017
5xL024
2xL049
ICxL026
7xL028
3xL039
3xL025
6xL025
5xL045
5xL044
5xL038
4xL025
5xL032
3xL025
5xL042
5xL027
5xL047
2xL025
IBxL026
5xL043
ICxL025
5xL028
IBxL025
