# Supplementary material for: Bayesian analysis and prediction of hybrid performance
Source: Plant Methods. 2019 Feb 7;15:14. doi: 10.1186/s13007-019-0388-x (PMC6366084; doi:10.1186/s13007-019-0388-x)
Supplement: Supplementary file 6 [file 13007_2019_388_MOESM18_ESM.docx]

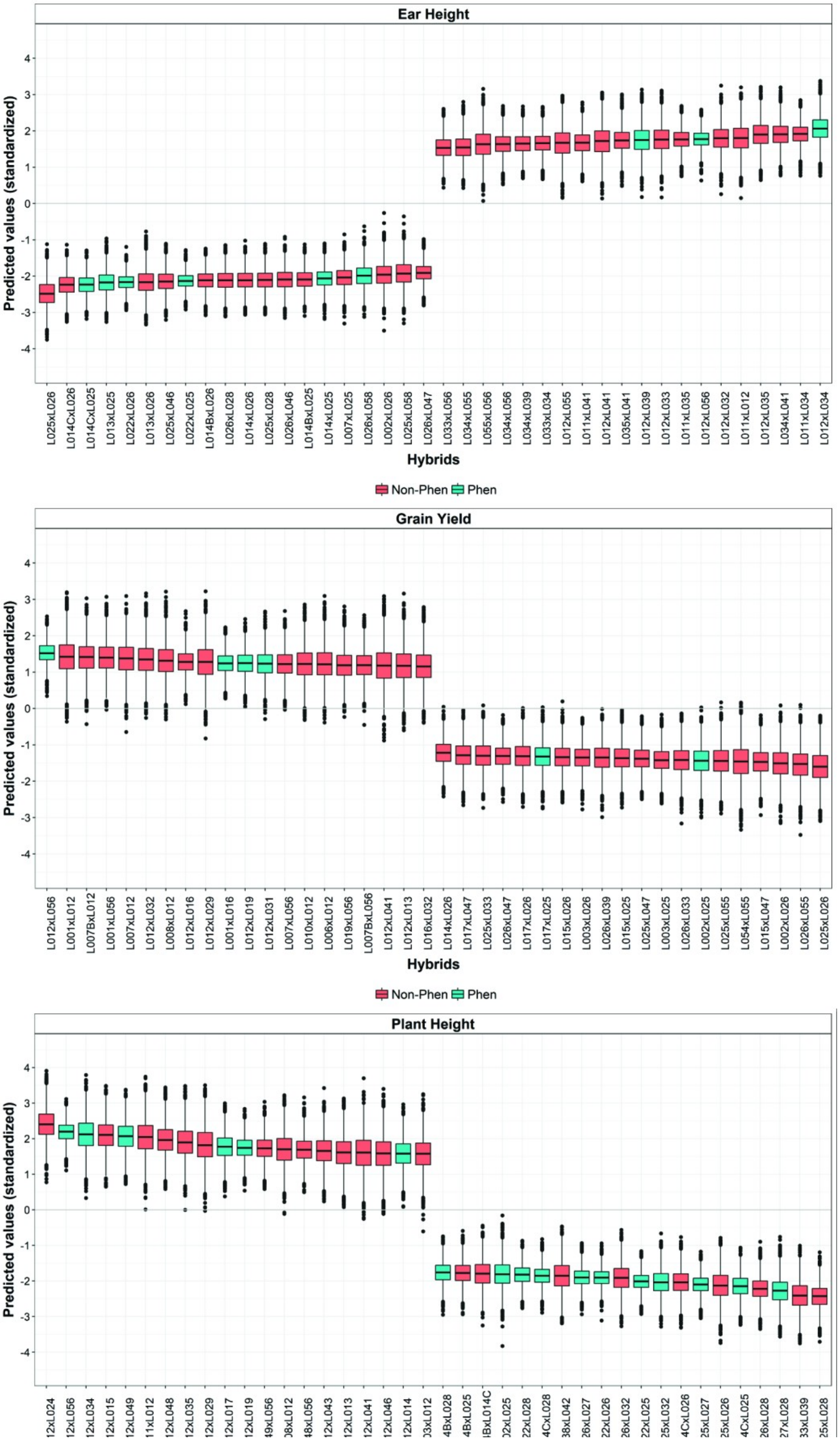

Ear Height
Predicted values (standardized)
4
3
2
1
0
-1
-2
-3
-4
L025xL026
L014CxL026
L014CxL025
L013xL025
L022xL026
L013xL026
L025xL046
L022xL025
L014BxL026
L026xL028
L014xL026
L025xL028
L026xL046
L014BxL025
L014xL025
L007xL025
L026xL058
L002xL026
L025xL058
L026xL047
L033xL056
L034xL055
L055xL056
L034xL056
L034xL039
L033xL034
L012xL055
L011xL041
L012xL041
L035xL041
L012xL039
L012xL033
L011xL035
L012xL056
L012xL032
L011xL012
L012xL035
L034xL041
L011xL034
L012xL034
Hybrids
Non-Phen
Phen
Grain Yield
Predicted values (standardized)
4
3
2
1
0
-1
-2
-3
-4
L012xL056
L001xL012
L007BxL012
L001xL056
L007xL012
L012xL032
L008xL012
L012xL016
L012xL029
L001xL016
L012xL019
L012xL031
L007xL056
L010xL012
L006xL012
L019xL056
L007BxL056
L012xL041
L012xL013
L016xL032
L014xL026
L017xL047
L025xL033
L026xL047
L017xL026
L017xL025
L015xL026
L003xL026
L026xL039
L015xL025
L025xL047
L003xL025
L026xL033
L002xL025
L025xL055
L054xL055
L015xL047
L002xL026
L026xL055
L025xL026
Hybrids
Non-Phen
Phen
Plant Height
Predicted values (standardized)
4
3
2
1
0
-1
-2
-3
-4
12xL024
12xL056
12xL034
12xL015
12xL049
11xL012
12xL048
12xL035
12xL029
12xL017
12xL019
49xL056
08xL012
48xL056
12xL043
12xL013
12xL041
12xL046
12xL014
33xL012
4BxL028
4BxL025
BxL014C
02xL025
22xL028
4CxL028
38xL042
26xL027
22xL026
26xL032
22xL025
25xL032
4CxL026
25xL027
25xL026
4CxL025
26xL028
27xL028
33xL039
25xL028
