# Supplementary material for: Bayesian analysis and prediction of hybrid performance
Source: Plant Methods. 2019 Feb 7;15:14. doi: 10.1186/s13007-019-0388-x (PMC6366084; doi:10.1186/s13007-019-0388-x)
Supplement: Supplementary file 7 [file 13007_2019_388_MOESM19_ESM.docx]

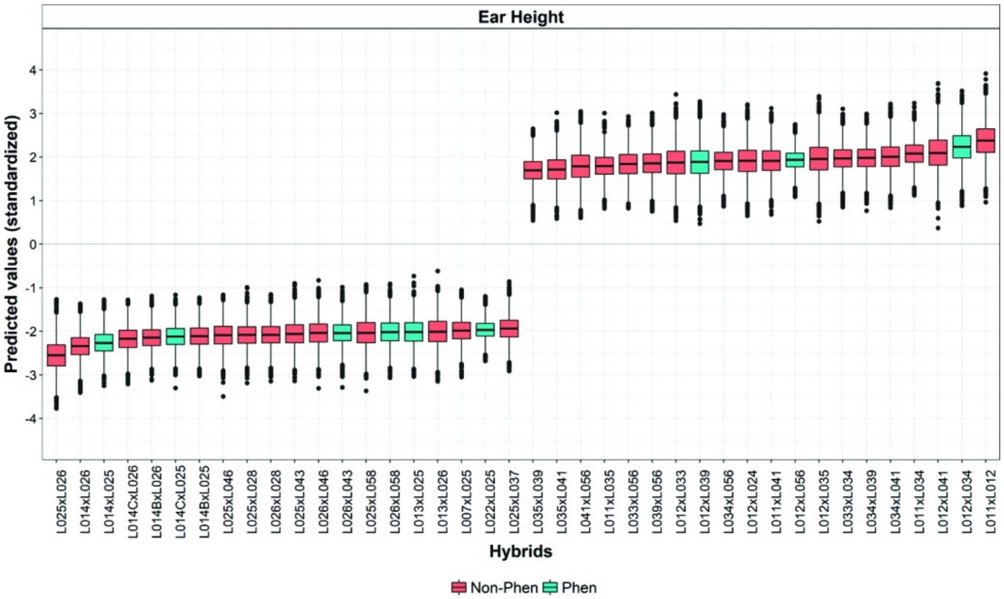
Ear Height
Predicted values (standardized)
4
3
2
1
0
-1
-2
-3
-4
L025xL026 L014xL026 L014xL025 L014CxL026 L014BxL026 L014CxL025 L014BxL025 L025xL046 L025xL028 L026xL028 L025xL043 L026xL046 L026xL043 L025xL058 L026xL058 L013xL025 L013xL026 L007xL025 L022xL025 L025xL037 L035xL039 L035xL041 L041xL056 L011xL035 L033xL056 L039xL056 L012xL033 L012xL039 L034xL056 L012xL024 L011xL041 L012xL056 L012xL035 L033xL034 L034xL039 L034xL041 L011xL034 L012xL041 L012xL034 L011xL012
Hybrids
Non-Phen
Phen

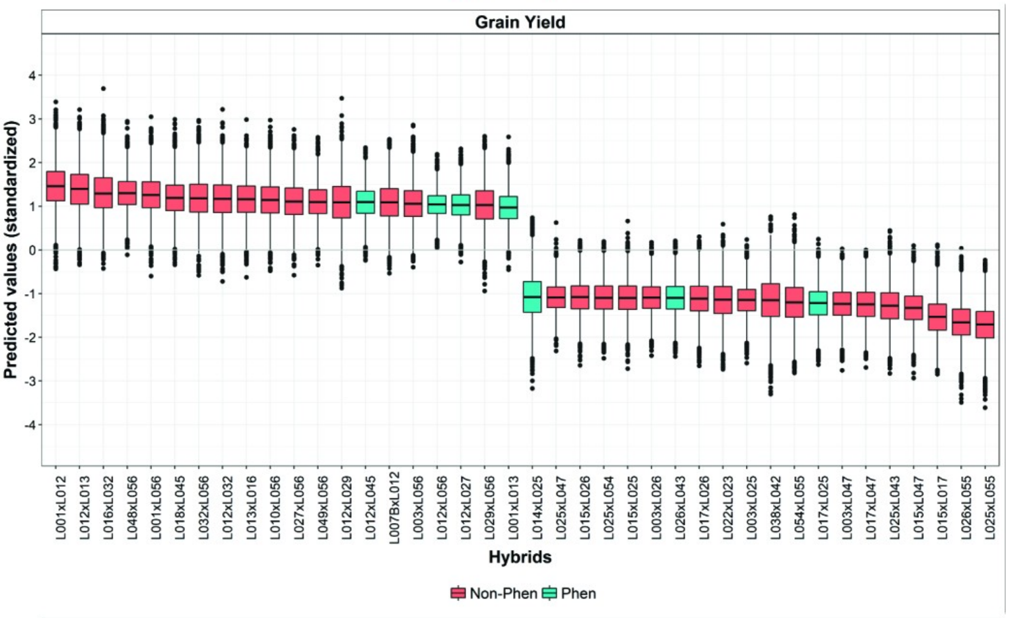
Grain Yield
Predicted values (standardized)
4
3
2
1
0
-1
-2
-3
-4
L001xL012 L012xL013 L016xL032 L048xL056 L001xL056 L018xL045 L032xL056 L012xL032 L013xL016 L010xL056 L027xL056 L049xL056 L012xL029 L012xL045 L007BxL012 L003xL056 L012xL056 L012xL027 L029xL056 L001xL013 L014xL025 L025xL047 L015xL026 L025xL054 L015xL025 L003xL026 L026xL043 L017xL026 L022xL023 L003xL025 L038xL042 L054xL055 L017xL025 L003xL047 L017xL047 L025xL043 L015xL047 L015xL017 L026xL055 L025xL055
Hybrids
Non-Phen
Phen

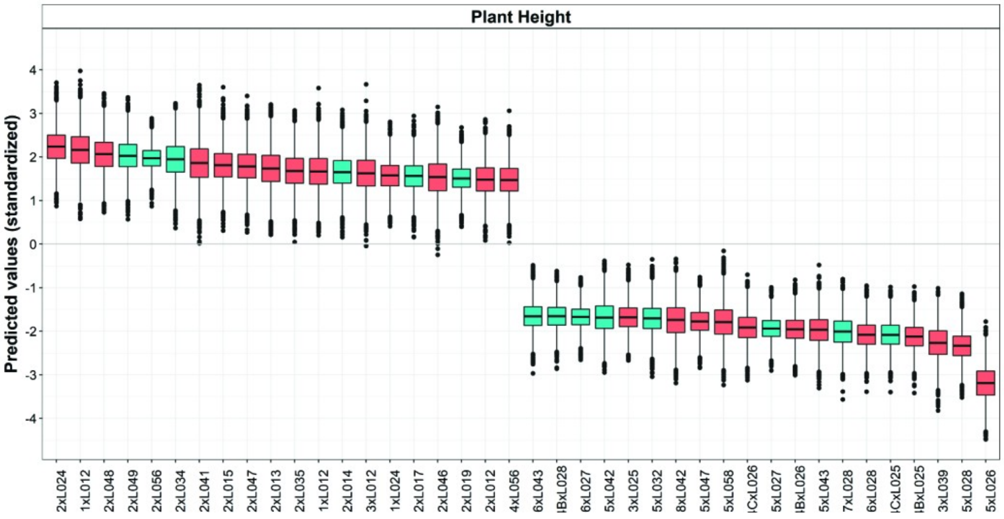
Plant Height
Predicted values (standardized)
4
3
2
1
0
-1
-2
-3
-4
2xL024 1xL012 2xL048 2xL049 2xL056 2xL034 2xL041 2xL015 2xL047 2xL013 2xL035 1xL012 2xL014 3xL012 1xL024 2xL017 2xL046 2xL019 2xL012 4xL056 6xL043 IBxL028 6xL027 5xL042 3xL025 5xL032 8xL042 5xL047 5xL058 ICxL026 5xL027 IBxL026 5xL043 7xL028 6xL028 ICxL025 IBxL025 3xL039 5xL028 5xL026
