# Supplementary material for: Bayesian analysis and prediction of hybrid performance
Source: Plant Methods. 2019 Feb 7;15:14. doi: 10.1186/s13007-019-0388-x (PMC6366084; doi:10.1186/s13007-019-0388-x)
Supplement: Supplementary file 8 [file 13007_2019_388_MOESM3_ESM.docx]

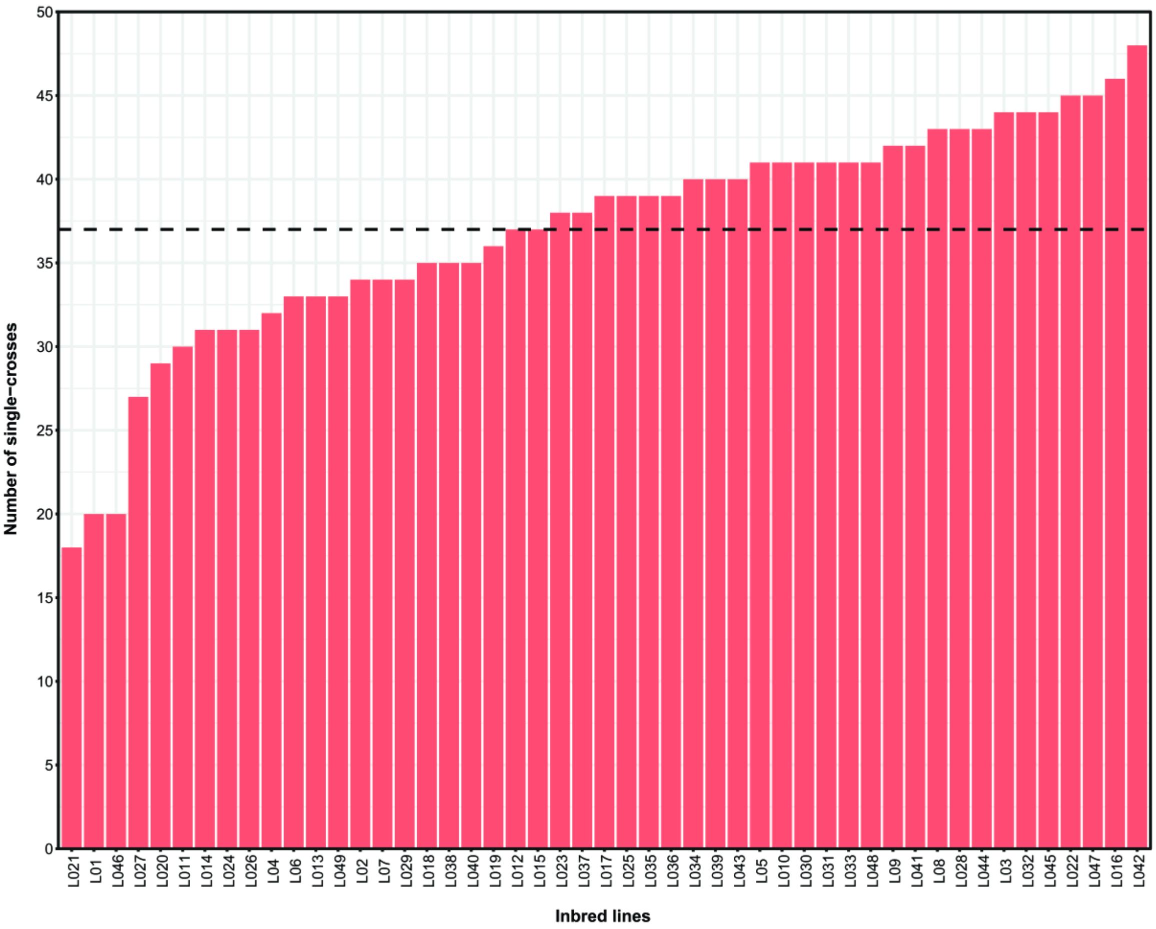
Number of single-crosses
50
45
40
35
30
25
20
15
10
5
0
L021
L01
L046
L027
L020
L011
L014
L024
L026
L04
L06
L013
L049
L02
L07
L029
L018
L038
L040
L019
L012
L015
L023
L037
L017
L025
L035
L036
L034
L039
L043
L05
L010
L030
L031
L033
L048
L09
L041
L08
L028
L044
L03
L032
L045
L022
L047
L016
L042
Inbred lines
